# The velvet spiders: an atlas of the Eresidae (Arachnida, Araneae)

**DOI:** 10.3897/zookeys.195.2342

**Published:** 2012-05-17

**Authors:** Jeremy A. Miller, Charles E. Griswold, Nikolaj Scharff, Milan Řezáč, Tamás Szűts, Mohammad Marhabaie

**Affiliations:** 1 Department of Terrestrial Zoology, Netherlands Centre for Biodiversity Naturalis, Postbus 9517 2300RA Leiden, The Netherlands; 2 Department of Entomology, California Academy of Sciences, 55 Music Concourse Drive, Golden Gate Park, San Francisco, California 94118, USA; 3 Department of Entomology, Zoological Museum, University of Copenhagen, Universitetsparken 15, 2100, Copenhagen, Denmark; 4 Center for Macroecology, Evolution and Climate, Department of Biology, University of Copenhagen, Universitetsparken 15, 2100 Copenhagen, Denmark; 5 Department of Entomology, Crop Research Institute, Drnovská 507, CZ-161 06 Prague 6 - Ruzyně, Czechia; 6 Department of Biology and Integrated Bioscience Program, University of Akron, Akron, Ohio 44325-3908, USA

**Keywords:** ladybird spiders, molecular phylogeny, spinneret spigot morphology, taxonomy

## Abstract

The family Eresidae C. L. Koch, 1850 is reviewed at the genus level. The family comprises nine genera including one new genus. They are: *Adonea* Simon, 1873, *Dorceus* C. L. Koch, 1846, *Dresserus* Simon, 1876, *Eresus* Walckenaer, 1805, *Gandanameno* Lehtinen, 1967, *Loureedia* **gen. n.**, *Paradonea*Lawrence, 1968, *Seothyra* Purcell, 1903, and *Stegodyphus* Simon, 1873. A key to all genera and major lineages is provided along with corresponding diagnoses, as well as descriptions of selected species. These are documented with collections of photographs, scanning electron micrographs, and illustrations. A new phylogeny of Eresidae based on molecular sequence data expands on a previously published analysis. A species of the genus *Paradonea* Lawrence, 1968 is sequenced and placed phylogenetically for the first time. New sequences from twenty *Gandanameno* Lehtinen, 1967 specimens were added to investigate species limits within the genus. The genus *Loureedia* **gen. n.** is proposed to accommodate *Eresus annulipes* Lucas, 1857. Two species, *Eresus semicanus* Simon, 1908 and *Eresus jerbae* El-Hennawy, 2005, are synonymized with *Loureedia annulipes* **comb. n.** One new species, *Paradonea presleyi* **sp. n.** is described. *Eresus algericus* El-Hennawy, 2004 is transferred to *Adonea* Simon, 1873. The female of *Dorceus fastuosus* C. L. Koch, 1846 is described for the first time. The first figures depicting *Paradonea splendens* (Lawrence, 1936) are presented.

## Introduction

The Eresidae, commonly known as velvet spiders (Dippenaar-Schoeman and Jocqué 1997; Dippenaar-Schoeman and Van den Berg 1988), comprise nearly 100 known species organized into nine genera, one of them newly described. Most genera are found principally in arid areas of Africa and Eurasia although some species are found in rainforests of the Afrotropical and Neotropical regions (Platnick 2011). Many eresids are cryptic sit-and-wait predators in deserts (Dippenaar-Schoeman 1990; El-Hennawy 2002). Members of the genus *Stegodyphus* Simon, 1873 typically build silken nests in vegetation (Fig. 4J–L) while other eresids typically live in silk tubes under objects (e.g., bark, stones) or underground (Fig. 4A–I). *Stegodyphus* exhibit varying degrees of solitary and subsocial behavior, and at least three species have independently evolved quasisocial behavior (Johannesen et al. 2007; Kraus and Kraus 1988; Seibt and Wickler 1988). Quasisocial means that these spiders live in groups throughout their lives and are non-territorial within the colony (Avilés 1997). Quasisocial *Stegodyphus* colonies may contain hundreds of closely related individuals that participate in dramatic mass attacks on prey (Fig. 3E–F) and form biotic islands with a characteristic fauna of kleptoparasites, parasites and inquilines (Fig. 3E, H; Griswold and Meikle-Griswold 1987; Griswold and Meikle 1990; Henschel et al. 1996). The bright red and black males of European species of *Eresus* Walckenaer, 1805, colloquially known as ladybird spiders, are among the most beautiful spiders in Europe, if not the world (Fig. 2B, D). In spite of their superficial resemblance to jumping spiders (Salticidae) and palp-footed spiders (Palpimanidae), or perhaps because of it, the phylogenetic placement of eresids has long been problematic. Although some genera have been revised in recent decades (Dippenaar-Schoeman 1990; El-Hennawy 2002; Kraus and Kraus 1988), the limits and distinguishing features of most genera are not well understood. In this paper we review the taxonomic and phylogenetic history of eresids, briefly summarize the biology of the family, redescribe the known genera and describe one new genus, provide diagnoses for these, provide the first key to the genera of Eresidae since Simon (1892), and present a new phylogenetic hypothesis based on molecular sequence data for all genera.

**Figure 1.**
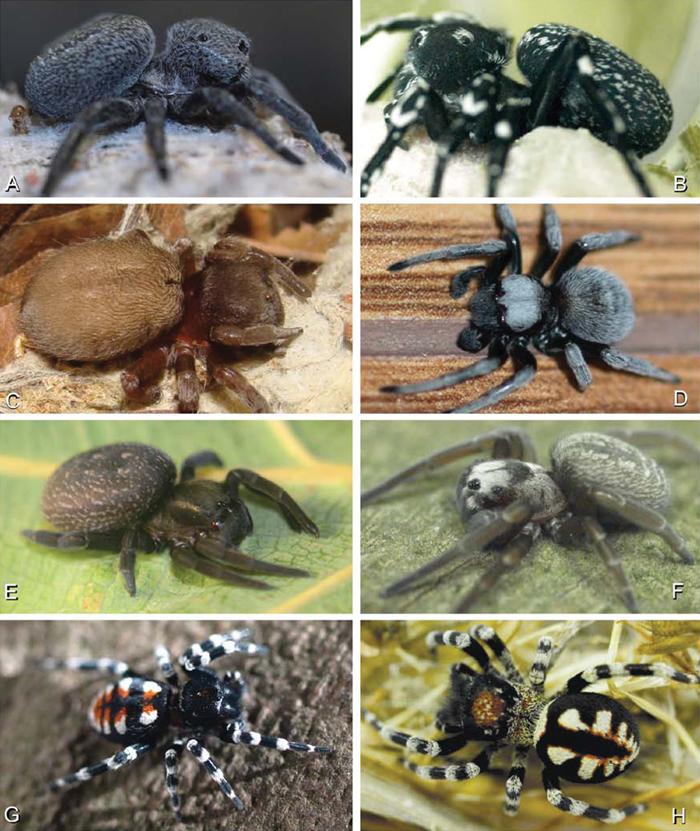
**A–H** Habitus of living Eresidae, photographs. **A, B** *Adonea fimbriata* **A** juvenile female (photo by Martin Forman) **B** adult male from Israel (photo by Martin Forman) **C** *Dresserus kannemeyeri*, adult female from Ndumo Game Reserve, South Africa (photo Stanislav Macík) **D** *Dresserus* sp., adult malefrom Namibia, between the towns Aus and Helmeringhausen (26°13.049'S, 16°36.063'E; photo by Martin Forman) **E, F** *Gandanameno* sp. **E** subadult female from Cape Town, South Africa (Stanislav Macík) **F** adult male from Anysberg Nature Reserve, Western Cape Province, South Africa (photo Martin Forman) **G, H** adult male *Loureedia annulipes*; **G** from Tel Krayot, Israel (photo by Martin Forman) **H** from Arad, Israel (photo by Martin Forman).

**Figure 2.**
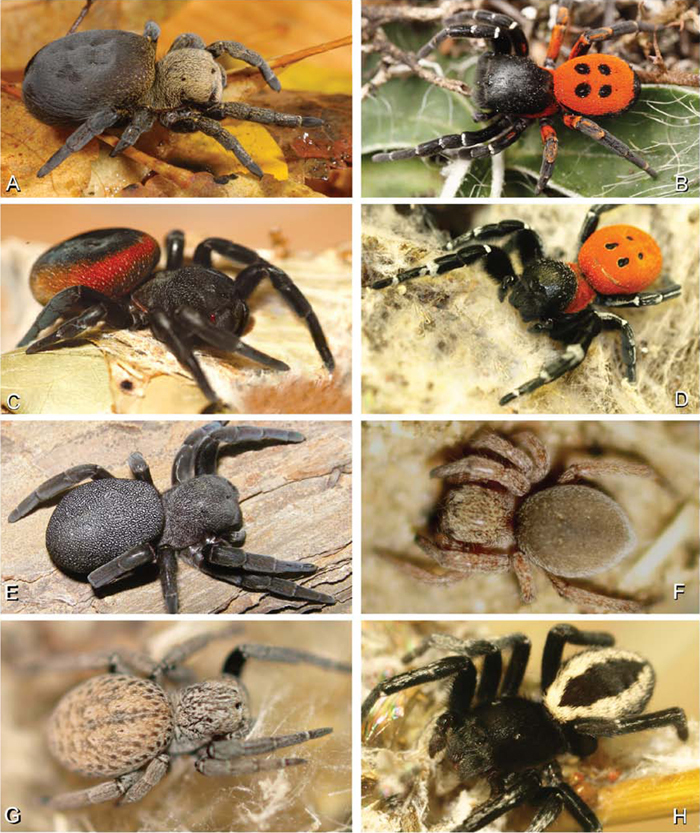
**A–H** Habitus of living Eresidae, photographs. **A, B** *Eresus kollari* **A** adult female from Hungary (photo by Tamás Szűts) **B** adult male from Kadaň, Czechia, (photo by Pavel Krásenský) **C, E** *Eresus walckenaeri*; **C** adult female from Greece (photo by Sergio Henriques) **D** adult male from Mihas, Greece (photo by Martin Forman) **E** juvenile female **F** *Seothyra* sp., juvenile female, from Brandberg, Namibia (photo by Martin Forman) **G, H** *Paradonea variegata* (photos by Martin Forman) **G** juvenile female from Betta, Namibia **H** adult male from Homeb, Namibia.

**Figure 3.**
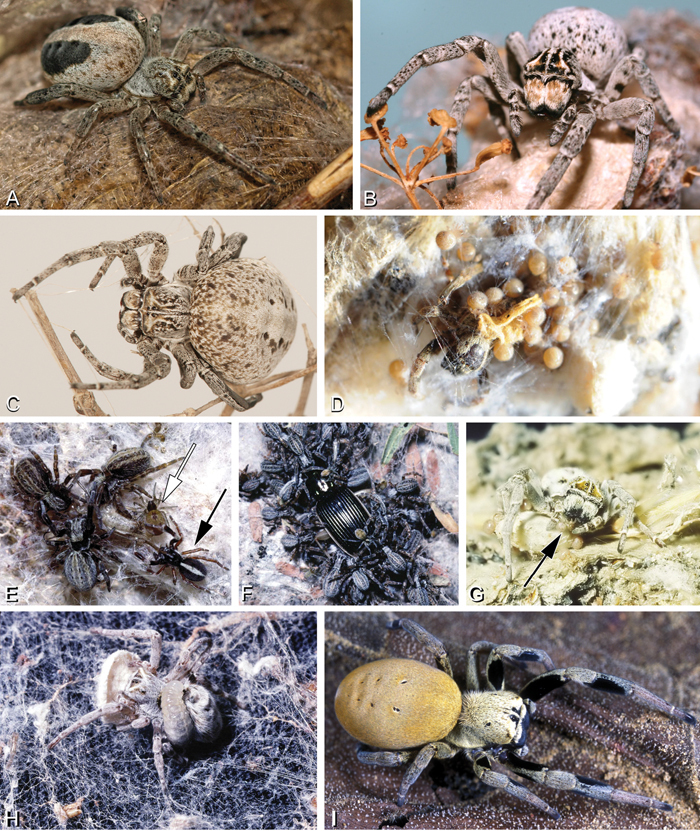
**A–I** Habitus of living Stegodyphus, photographs. **A–C** *Stegodyphus lineatus* **A** adult female from Hurghada, Egypt **B** adult female from Negev desert, Israel (photo by Rudolf Macek) **C** adult female from Shoam, Israel (photo by Amir Weinstein) **D** juvenile *Stegodyphus tibialis* feeding on their mother, Dali, China (photo by Yang Zi-Zhong) **E** *Stegodyphus mimosarum*, male (black arrow), females and a kleptoparasite Archeodictyna (white arrow) **F** *Stegodyphus mimosarum*, mass attack on a carabid **G** a female *Stegodyphus dumicola* feeding her offsprings **H** a pompilid wasp larva feeds on a female *Stegodyphus dumicola* (photos E–H by Teresa Meikle) **I** *Stegodyphus* sp. female from ShweSettaw Wildlife Reservation, Myanmar (photo by Dong Lin).

**Figure 4.**
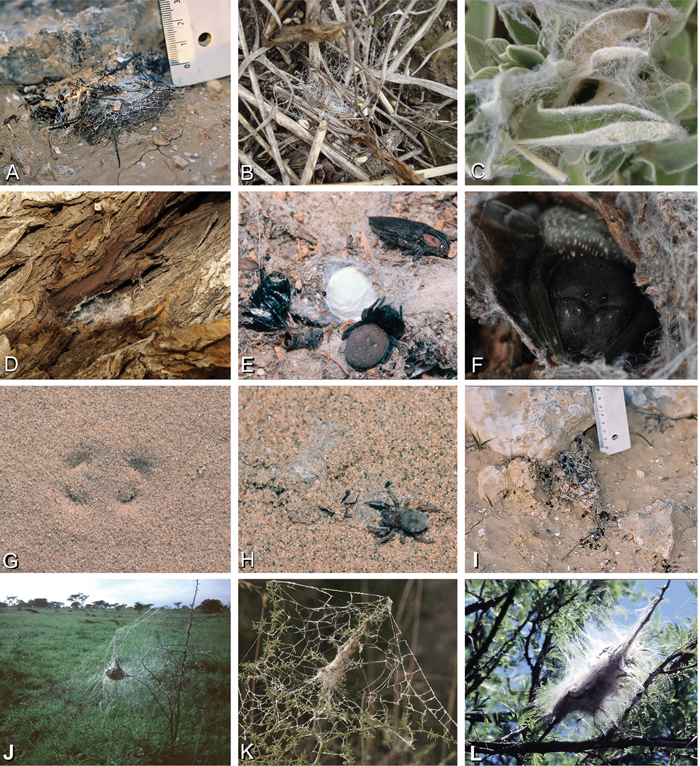
**A–L** Retreats, webs, and habitus of living Eresidae, photographs. **A** *Adonea fimbriata* retreat on the ground from Sede Boqer, Israel (photo by Efrat Gavish-Regev) **B** *Dresserus* sp. retreat in the grass (photo by Charles Haddad) **C** *Eresus walckenaeri* retreat of juvenile from Ioannina, Greece (photo by Siegfried Huber) **D–E** *Gandanameno* sp. from west of Helmeringhausen Namibia (photos by Martin Forman) **D** Retreat on *Acacia* **E** female, with egg sac and various prey remnants **F** *Gandanameno* sp. femalefrom Amanzi Game Reserve, South Africa (photo by Tamás Szűts) **G, H** *Seothyra* sp. from Namibia **G** retreat under sand, showing the antelope track pattern **H** specimen and the exposed retreat (photos **E–H, J, L** by Teresa Meikle) **I** *Loureedia annulipes*, burrow from Sede Boqer, Israel (photo by Efrat Gavish-Regev) **J–L** *Stegodyphus* retreats **J** *Stegodyphus dumicola* from Spioenkop, South Africa **K** *Stegodyphus lineatus* from Tel-Hadid, Israel (photo by Amir Weinstein) **L** *Stegodyphus mimosarum* from Spioenkop, South Africa.

### Taxonomic and phylogenetic history of Eresidae

Taxonomic research on Eresidae goes back to 1778, when Martini and Goeze described a male from Bavaria, Germany, and named it *Aranea sandaliata* (Lister 1778). In the following decade, the spectacular *Eresus* males were described four times under different names without reference to the already described species (Olivier 1789; Petagna 1792; Rossi 1790; Villers 1789). Hahn (1821), Brullé (1832) and Koch (1836) were the first to distinguish more than one *Eresus* species. However, they used coloration as the only character for discrimination and elevated some infraspecific varieties to the specific level (e.g., Koch 1836; 1846). Due to the remarkable sexual dimorphism, males and females of the same species were given different names (Brullé 1832; Koch 1846). Koch (1850) even established a new genus, *Erythrophorus*, for *Eresus* males. Unfortunately, insufficient descriptions and lost type material during this early phase of taxonomical study brought about considerable confusion in the nomenclature of eresids. Řezáč et al. (2008) believe that *Eresus kollari* Rossi, 1846 is the earliest identifiable name for the widespread species of this genus. Over the next century additional eresid genera were discovered: *Dorceus* C. L. Koch, 1846, *Adonea* Simon, 1873, *Stegodyphus* Simon, 1873, *Dresserus* Simon, 1876, *Penestomus* Simon, 1902, *Seothyra* Purcell, 1903, *Magunia* Lehtinen, 1967, *Wajane* Lehtinen, 1967 and *Paradonea* Lawrence, 1968.

Eresidae as a family-level taxon was established by Koch, who called it Eresides (Koch 1850: 70). Suffixes of family group names (e.g., -idae) were not standardized until the publication of the Règles Internationales de la Nomenclature Zoologique (International Commission on Zoological Nomenclature 1905), although it is recommended at least as far back as the non-binding Strickland Code (Strickland et al. 1843). There is some confusion over the correct date of the publication establishing Eresidae. Bonnet (1945, 1956) gives the date of this publication as 1851, in contradiction with the date on the frontispiece. No supporting evidence is presented to justify the later date. By contrast, Roewer (1942, 1954) accepted the date of Koch’s publication as 1850 in his catalog. Determining the correct publication date of certain classic works in zoology can be problematic. In a paper investigating the publication dates of some works in arachnology, Brignoli (1985) argued that the evidence for the 1850 date was stronger than the alternative.

Eresidae was historically divided into two subfamilies: Eresinae and Penestominae (Simon 1903). Penestomines have a controversial history (see Miller et al. 2010a; Miller et al. 2010b). Based on the results of a molecular phylogenetic analysis, Miller et al. (2010a) removed Penestominae from Eresidae. As currently circumscribed, Eresidae are three-clawed, cribellate spiders characterized by a subrectangular carapace, median eyes grouped together and subtended by a clypeal hood, ALE placed near the anterior lateral corners of the carapace, and a strongly recurved PER (a key to anatomical abbreviations is given in the Methods section, below). The male palp has a conductor that interacts with the embolus, but lacks a median apophysis and retrolateral tibial apophysis.

Nearly all eresids are distributed in Europe, Africa, and Asia, but records from Brazil also exist. In the original description of *Eresus annulipes* Lucas, 1857 (transferred herein to a new genus), the author gives “Rio-Janeiro” Brazil as the locality. However, the vial with the type specimen (examined by MR) includes a label indicating that the locality is unknown (“patria ignota”). Subsequent collections indicate that this species comes from arid parts of the Mediterranean. A second Brazilian eresid, *Stegodyphus manaus* Kraus and Kraus, 1992 appears to be a legitimate eresid not known from any other part of the world (Kraus and Kraus 1992).

### Phylogenetic placement and arrangement of Eresidae

Presumably because of the broad carapace and ocular area and widely dispersed eyes, Eresidae was traditionally associated with families such as Palpimanidae and Salticidae. Koch (1837) placed *Eresus* in his Attides, and later selected this as the type genus of the family level group Eresides (Koch 1850). Koch (1850) included the genus *Palpimanus*, along with *Dorceus*, *Eresus*, and *Erythrophorus* (representing *Eresus* males), in his original circumscription of the family. Placement continued to vary: Doleschall (1852) and Blackwall (1861) placed *Eresus* in Salticidae and O. Pickard-Cambridge (1872) placed the genus in Dictynides. Eresinae was used as a subfamily by Simon (1903) and this usage as a valid family has remained stable since.

In a landmark paper in spider systematics, Lehtinen (1967) examined all known genera of eresids, described two new genera (*Gandanameno* and *Magunia*, the latter synonymized with *Stegodyphus* by Kraus and Kraus 1988), produced a comparative table for a wide variety of somatic and genitalic characters (tables 46 and 47), and offered a branching diagram depicting his hypothesis of phylogenetic relationships among eresid genera (Fig. 7A; Lehtinen 1967: fig. 13). Notable were his association of *Dresserus* with *Gandanameno*, genera with modified PMS, and of *Dorceus* with *Seothyra*, sand dwelling genera with telescoping ALS. Lehtinen placed the Eresidae in his higher group Zodariides, along with the Thomisoidea and Salticoidea and the diverse Zodarioidea, this latter group including taxa traditionally associated with eresids such as Palpimanidae and Zodariidae. Lehtinen (1967: 385) admitted that “limitation of this group remains rather vague” and that “it might include several polyphyletic groups.” Lehtinen’s Zodariides did not gain widespread acceptance, and subsequent phylogenetic analyses corroborated Lehtinen’s initial misgivings about the naturalness of this group. In contrast, Levi (1982) placed Eresidae in the Eresoidea, including Eresidae, Hersiliidae and Oecobiidae, a suggestion followed by Coddington and Levi (1991) and corroborated by the analyses of Griswold et al. (1999) and Griswold et al. (2005). Wunderlich (2004) proposed a considerably different concept of Eresoidea excluding Hersiliidae and Oecobiidae and including Archaeidae (including Mecysmaucheniidae), Huttoniidae, and Palpimanidae (including Stenochilidae), plus the extinct Lagonomegopidae and Spatiatoridae. Although Wunderlich did not present a matrix-based phylogenetic analysis, he did exhibit tree-based thinking in the organization of his character evidence. Wunderlich (2004: 761) specified the following characters as apomorphies of his Eresoidea: a large raised cephalic region, rugose and heavily sclerotized prosoma, strong front legs, wide eye field, widely spaced median and lateral eyes, loss of dorsal and lateral leg bristles, reduced cheliceral teeth, and small male and female palpi. He also noted that the conformation of the palpal bulb is basically similar in Eresidae, Palpimanidae, and Spatiatoridae, characterized by a protruding (sub)tegulum, an embolus and conductor typically originating in a distal position and directed to the tip of the cymbium, and the absence of a median apophysis. For Eresidae (including Penestomidae), Wunderlich (2004: 761) specified the following apomorphic characters: entelegyne female genitalia with only one pair of spermathecae, a short clypeus, median cheliceral keel, maternal feeding of offspring, molting of adults at least in females, and (excluding Penestomidae) sexual size dimorphism.

**Figure 5.**
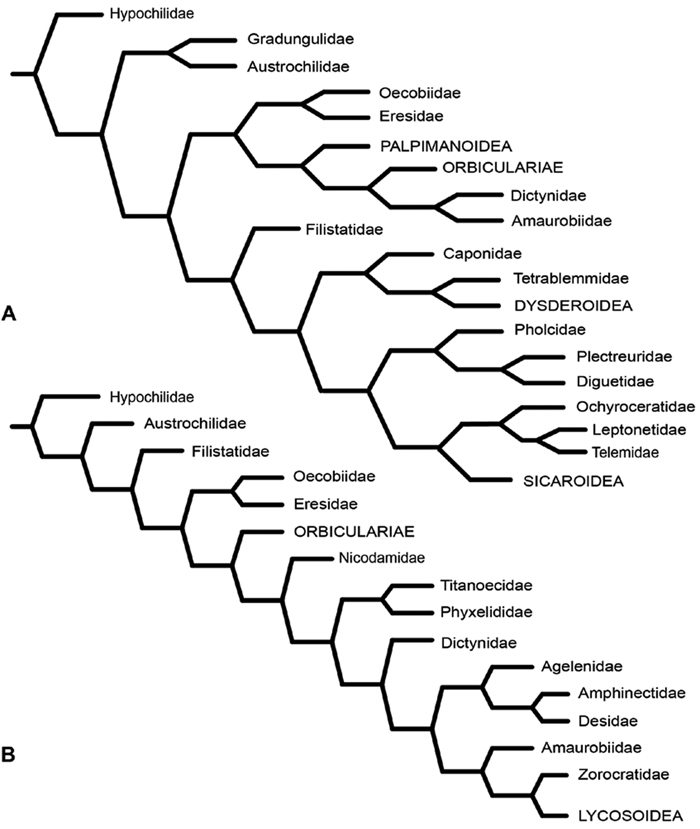
**A–B** Historical hypotheses of the phylogenetic position of Eresidae. **A** simplified cladogram from Platnick et al. (1991: 68, fig. 311) **B** simplified cladogram from Griswold et al. (1999: 58, fig. 1). Terminals merged into family level (normal type) or higher level (all capital type) taxa.

**Figure 6.**
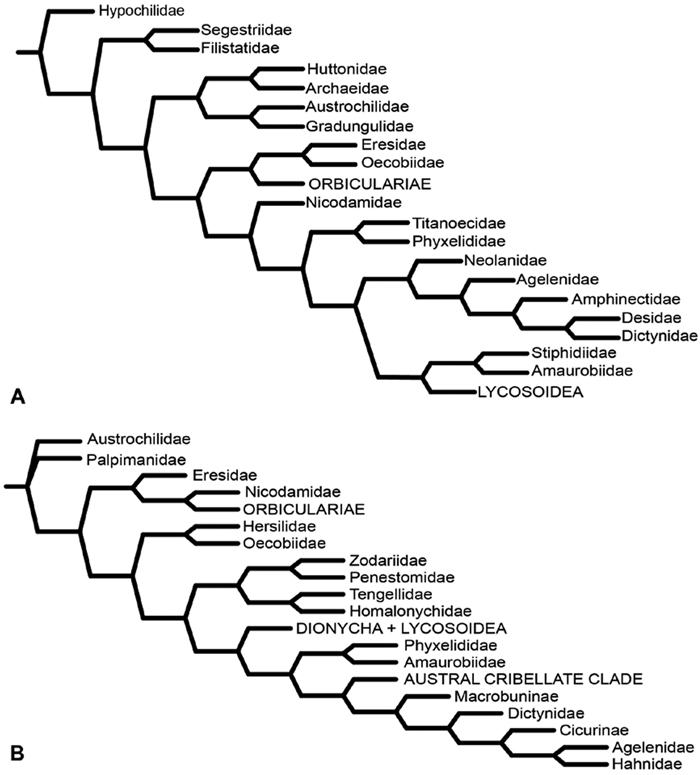
**A–B** Historical hypotheses of the phylogenetic position of Eresidae. **A** simplified implied weights cladogram (K=6, fit=115.93, L=488) from Griswold et al. (2005: 316, fig. 219) **B** simplified Bayesian tree from Miller et al. (2010a: 792, fig. 3). Terminals merged into family level (normal type) or higher level (all capital type) taxa.

After the widespread adoption of cladistic reasoning in Arachnology and advent of matrix based comparative data and computer assisted analyses, eresid placement and circumscription continued to evolve. Coddington (1990a; see also Coddington 1990b) became the first to include an eresid exemplar in a quantitative analysis. This analysis, designed primarily to test the hypothesis of orb-weaver monophyly, included the eresid *Stegodyphus*. This genus fell as outgroup to a clade comprising the Orbiculariae plus RTA clade, i.e., an amaurobiid, dictynid, psechrid and titanoecid. *Oecobius*, also included in the analysis, was not sister to *Stegodyphus*, in effect challenging Levi’s Eresoidea. In contrast, Platnick et al. (1991) corroborated Levi’s hypothesis: this study was the first to associate Eresidae (*Stegodyphus*) and Oecobiidae (*Oecobius*) in a quantitative cladistic analysis (Fig. 5A); synapomorphies were loss of paracribellum, MAP spigots dispersed with the PI field, and transverse ridges on the hood of the trichobothrial base. Griswold et al. (1999), using an expanded exemplar set of eresids and potential sister groups, associated Eresidae (*Stegodyphus* and *Eresus*) and Oecobiidae (*Oecobius* and *Uroctea*) as Eresoidea (Fig. 5B); Eresoidea synapomorphies were loss of paracribellum, multiple MAP spigots dispersed with the PI field, transverse ridges on the hood of the trichobothrial base, and divided cribellum. For the first time they also suggested a suite of synapomorphies for the Eresidae: a square to rectangular carapace, clypeal hood, presence of a cheliceral boss, loss of the tapetum, recurved PER, MAP shaft cuticle papillate or scaly, and loss of the MA on the palp. In keeping with Kovoor and Lopez’ (1979) studies of eresid silk glands, the PMS were interpreted as having multiple mAP and CY spigots and lacking AC spigots; these features also optimized as eresid synapomorphies. Schütt (2002) examined the limits of the orb building spiders (Araneoidea) and their kin. Her study had a sparse, but broad, sampling of taxa, including *Eresus* as eresid exemplar. She reinterpreted eresid spinning organs and, contra Kovoor and Lopez, recognized eresids as having a brush of AC spigots. Her analysis associated Eresidae with Palpimanoidea (*Palpimanus*, *Archaea* and *Afrarchaea*); her taxon sampling did not allow testing of Eresoidea. Griswold et al. (2005) produced a new, expanded analysis of entelegyne relationships, and also reinterpreted many of the spigot and silk gland characters used in previous studies. Using ontogeny as the prime criterion for recognizing spigot types, they coded eresids as having AC, mAP and CY spigots. Analyses under equal weights and implied weights (Fig. 6A) both supported an Eresoidea comprising Eresidae (*Eresus* and *Stegodyphus*) and Oecobiidae (*Oecobius* and *Uroctea*).

**Figure 7.**
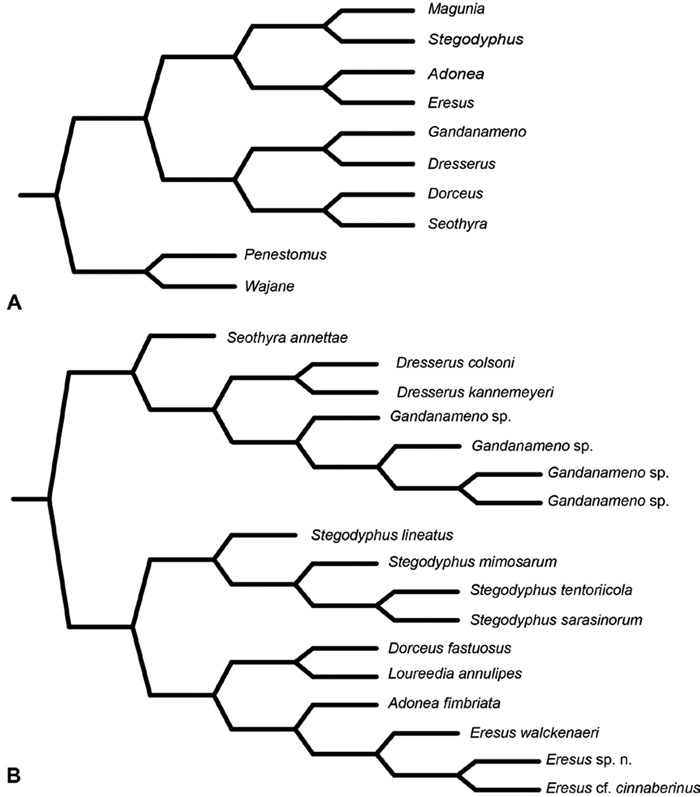
**A–B** Historical phylogenetic hypotheses of relationships among Eresidae. **A** tree from Lehtinen (1967: 387, fig. 13). *Magunia* was synonymized with *Stegodyphus* by Kraus and Kraus (1988); *Wajane* was synonymized with *Penestomus* and removed from Eresidae by Miller et al. (2010a; 2010b) **B** relationships among Eresidae based on molecular phylogenetic analysis of Miller et al. (2010a: 792, modified fig. 3). “*Stegodyphus*" *annulipes* relabeled *Loureedia annulipes* to reflect a nomenclatural change proposed in this work and *Gandanameno* species epithets removed to reflect increasing uncertainty about species limits and identity in this genus.

Miller et al. (2010a) carried out a study relevant to eresid phylogeny and placement that was novel in three ways: a dense sampling of eresid genera, a broad array of entelegyne taxa, and data from 4 molecular markers (28S rDNA, 18s rDNA, H3 and CO1). This analysis included representatives of all Eresoid families (Eresidae, Oecobiidae and Hersiliidae), seven of the eight eresid genera (*Paradonea* was unavailable), and an additional 54 exemplars from across the Entelegynae. Notable results were that: 1) Eresoidea was never corroborated: eresids grouped with the orbicularian *Zygiella* and the nicodamids *Megadictya* and *Oncodamus*, 2) Penestominae never grouped with Eresinae, but with zodariids, leading to the proposal of the new family Penestomidae (Fig. 6B), and 3) a phylogeny for all eresid genera except *Paradonea* was proposed (Fig. 7B). Association of Eresidae with Nicodamidae and Orbiculariae was anticipated and corroborated by the molecular studies of Spagna and Gillespie (2008) and of Spagna et al. (2010). Eresidae was divided into two major clades: *Seothyra*, *Dresserus*, and *Gandanameno* form a southern and eastern African clade. *Seothyra* is exclusively southern African, whereas the sister genera *Dresserus*, and *Gandanameno* occur in southern and eastern Africa. The other major clade comprises *Stegodyphus*, *Eresus*, *Adonea*, and *Dorceus*; *Eresus*, *Adonea*, and *Dorceus* form a Palearctic/Mediterranean clade; *Stegodyphus* is found in Africa, the paleotropics, and the Amazon.

### Spinneret spigot morphology

The silk glands and spinnerets of Eresidae have been discussed previously but their interpretation remains controversial. Kovoor and Lopez (1979) studied the silk glands of the eresids *Eresus kollari* (as *Eresus niger*) and *Stegodyphus dufouri*. Peters (1992b) studied the spigots of two species of *Stegodyphus* and traced the origin of the fibers that composed the cribellate strands. Eresid spinnerets have been studied with scanning electron microscopy and coded in matrices by Coddington (1990a), Platnick et al. (1991), Griswold et al. (1999), Schütt (2002) and Griswold et al. (2005). These studies relied upon the position, number, fine structure and ontogeny of the spigots for their classification, and these were named assuming the glands that they served, a departure from the gland-based studies of Kovoor and Lopez. Kovoor and Lopez (1979) identified pseudoflagelliform glands in eresids. Griswold et al. (1999) and Griswold et al. (2005) asserted homology between the pseudoflagelliform gland spigots of Deinopoidea and a unique spigot on the PLS of females, which they termed the “modified spigot” (MS). These spigots were overlooked in eresids by Griswold et al. (1999), but were recognized by Griswold et al. (2005). Eresid MS are unique in being anterobasal on the PLS, far removed from the rest of the spinning field, though the MS may or may not be accompanied by flanking spigots (Figs 30D, F, 36E, 39D, 57D, 58C, 60D, 61B–C, 66D, 67D, 74B, 75F, 77D, 78D, 87D, 88D, 95D). Recognizing eresid MS spigots is therefore unproblematic. Eresids are unique in that their ampullate gland spigot (MAP, mAP) shafts have small papillae or imbricate protrusions, rendering these easy to recognize on the ALS and PMS (Figs 61A, D, 67A, C). Kovoor and Lopez (1979) further asserted that eresids have numerous ampullate and cylindrical glands but lack aciniform glands. Previous phylogenetic studies (Griswold et al. 1999) relied upon these gland data to code eresids as having numerous MAP, mAP, and CY and lacking AC, but Schütt (2002) coded eresids as having a brush of AC spigots and Griswold et al. (2005) relied upon ontogenetic data to recognize AC spigots as present. Nevertheless, distinguishing AC and CY spigots remains problematic. Whereas in “higher” entelegynes, e.g., Orbiculariae (Griswold et al. 1998), Gnaphosoidea (Platnick 1990) and the “austral cribellates” (Griswold et al. 2005) classes of spigots are both distinct and uniform, in the “lower entelegynes,” e.g., Oecobiidae, Eresidae, individual spigots vary in size and form such that, even with ontogenetic information, distinguishing AC and CY remains difficult in most cases. The field of small spigots with stout shafts located on the posterior lobe of the PMS of females (not males) of *Dresserus* and *Gandanameno* seems made of obvious CY spigots (Figs 36C–D, 57C, 58E). The situation in other eresids is more puzzling. Examining the PMS of *Stegodyphus*, Griswold et al. (2005) found three classes of spigots in females (large, medium and small) but only the large and small in males: the large were clearly mAP, the small likely AC (occurring in males and females) and the intermediate class, found only in females, were classified as CY. This ambiguity prevails in the other genera examined here, with the exception of *Dresserus* and *Gandanameno*, and our classification of AC and CY on the posterior spinnerets must remain provisional.

## Methods

### Scanning electron microscopy

Specimens were critical point dried, then mounted on stubs or round-headed rivets using a combination of white glue, nail polish, and adhesive copper or aluminum tape. They were sputter coated with platinum-palladium and scanned with one or more of the following: a JEOL JSM-6335F field emission scanning electron microscope, a JEOL JSM-840A scanning electron microscope, and a FEI Inspect scanning electron microscope, all at the Natural History Museum of Denmark, or a LEO 1450VP at the California Academy of Sciences. Electron micrographs of internal female reproductive structures were accomplished by first dissolving soft tissue from dissected epigyna in pancreatin P1750 enzyme (Álverez-Padilla and Hormiga 2008).

### Light microscopy

Specimens were photographed in dishes of alcohol or temporary slide mounts (Coddington 1983). Where necessary, positioning specimens was aided by the use of sand or commercial Purell Hand Sanitizer (http://www.purell.com/). Photographs were made using digital cameras mounted on microscopes, either a BK+ Imaging System fromVisionary Digital (http://www.visionarydigital.com/) based on a Canon 7D digital camera body and a K2 Infinity microscope fitted with various Infinity lenses and Nikon metallurgical objectives at the Zoological Museum, Copenhagen, a Leica M205A stereoscopic microscope equipped with a Leica DFC420 camera and Leica Applications Suite software at the Zoological Museum, Copenhagen, a Nikon DS-Ri1 driven by Nikon NIS Elements software mounted on a Leica M165 C stereomicroscope at the Netherlands Centre for Biodiversity Naturalis, Leiden, or a Leica DFC500 digital camera driven by Leica Applications Suite software mounted on a Leica MZ16A stereomicroscope at the California Academy of Sciences, San Francisco. Stacks of images from multiple focal planes were combined and edited using either Helicon Focus Professional MP or Auto-Montage Pro software version 5.03, and further processed in Photoshop CS5 to adjust color, brightness, and contrast, and remove blemishes. Female reproductive structures were cleared for images of internal structures using methyl salicylate (Holm 1979). In some cases, pancreatin was also used first to digest away soft tissue. To investigate expansion of the male palp, these were removed, boiled for 2–3 minutes in a bath of hot concentrated (92%) lactic acid solution (SIGMA-ALDRICH, Inc., St. Louis, USA), then transferred to warm distilled water where expansion took place. Expanded palpi were photographed in water and positioned using a temporary slide mount (Coddington 1983).

## Measurements and conventions

PLE position is expressed as a ratio of the distance from the anterior margin of the carapace to the anterior margin of the PLE divided by total carapace length. Eye diameter measured to the outside margin (analogous to measuring the cornea rather than the iris). Carapace length measured to the straight anterior margin excluding the clypeal hood.

**Figure 8.**
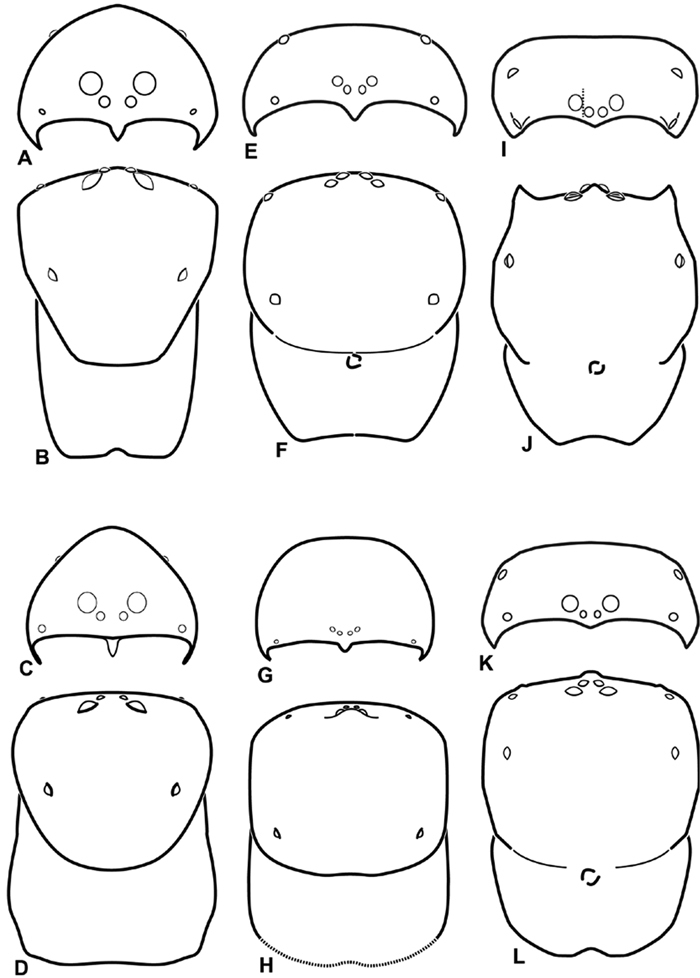
**A–L** Schematic illustrations of the carapace of assorted eresids **A–D** *Adonea fimbriata* **E–H** *Dorceus fastuosus* **I–L** *Dresserus* sp. **A–B, E–F, I–J** male **C–D, G–H, K–L** female **A, C, E, G, I, K** anterior view **B, D, F, H, J, L** dorsal view. Dashed line in **I** drawn tangential to the mesal margin of the PME does not intersect with the AME indicating median eyes separated on vertical axis. Dashed lines at posterior of carapace indicate uncertainty. Not to scale.

**Figure 9.**
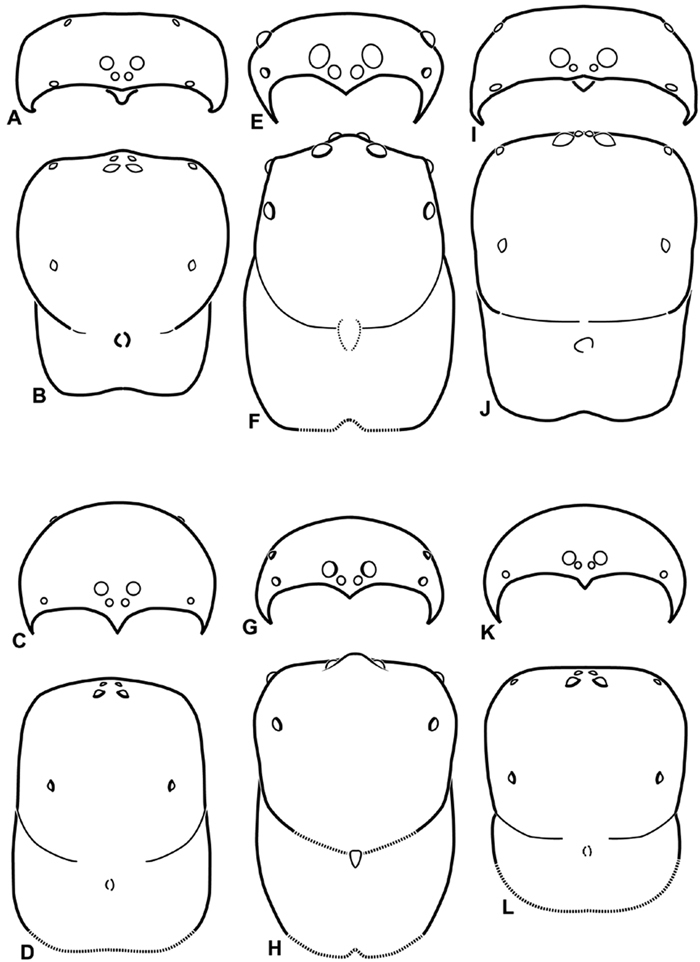
**A–L** Schematic illustrations of the carapace of assorted eresids. **A–D** *Eresus kollari* **E–H** *Gandanameno* sp. **I–L** *Loureedia annulipes* **A–B**, **E–F**, **I–J** male **C–D**, **G–H**, **K–L** female **A, C, E, G, I, K** anterior view **B, D, F, H, J, L** dorsal view. Dashed lines at posterior of carapace indicate uncertainty. Not to scale.

**Figure 10.**
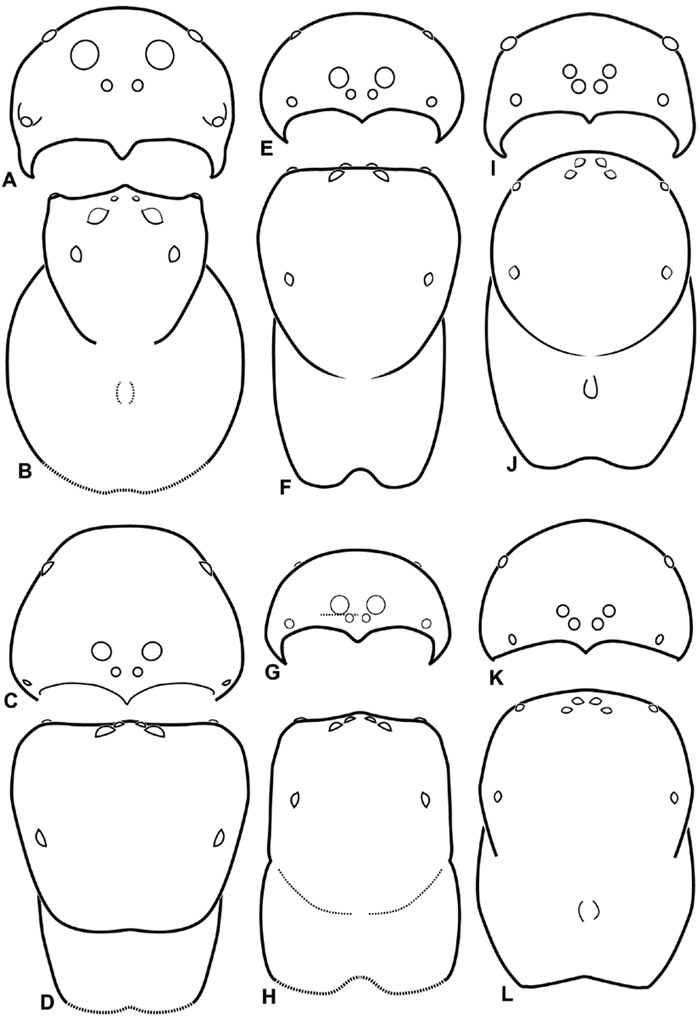
**A–L** Schematic illustrations of the carapace of assorted eresids. **A–B** *Paradonea striatipes* **C–D** *Paradonea splendens* **E–H** *Paradonea variegata* **I–L** *Seothyra henscheli* **A–D, E–F, I–J** male **G–H, K–L** female. **A, C, E, G, I, K** anterior view **B, D, F, H, J, L** dorsal view **G** illustrates example of median eyes overlapping on horizontal axis. Dashed lines at posterior of carapace indicate uncertainty. Not to scale.

**Figure 11.**
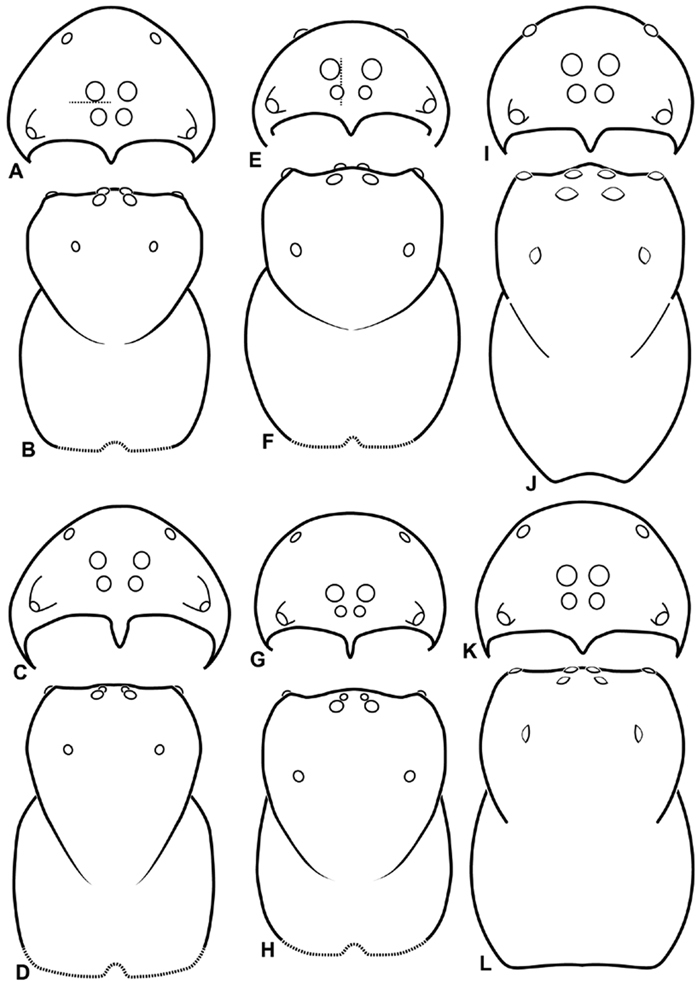
**A–L** Schematic illustrations of the carapace of assorted *Stegodyphus* species. **A–D** *Stegodyphus lineatus* **E–H** *Stegodyphus mimosarum* **I–L** *Stegodyphus sarasinorum*. **A–B, E–F, I–J** male **C–D, G–H, K–L** female **A, C, E, G, I, K** anterior view **B, D, F, H, J, L** dorsal view **A** illustrates example of median eyes separated on horizontal axis; E illustrates example of median eyes overlapping on vertical axis. Dashed lines at posterior of carapace indicate uncertainty. Not to scale.

We use the terms horizontal axis and vertical axis to describe the configuration of the median eyes. Overlapping on the horizontal axis means that if one drew a line connecting the ventral limits of the PMEs, it would pass through the AMEs (Fig. 10G); separated on the horizontal axis means that the line would not pass through the AME (Fig. 11A). Similarly, if one drew a perpendicular line tangential to the mesal limit of a PME and it passed through the corresponding AME, we call this overlapping on the vertical axis (Fig. 11E); separated on the vertical axis means that the line would not pass through the AME (Fig. 8I). Arrangement of the eyes and eye rows is depicted schematically in Figs 8–11.

Specimen collection data are given in Appendix A. Latitude-longitude coordinate pairs inferred from labels are given in square brackets; coordinates explicitly given on labels are not in square brackets. Some coordinate pairs are taken from the Iziko South African Museum (Cape Town) collections database are so indicated.

Descriptions were made mostly based on alcohol-preserved museum specimens. Some color information can be lost from such specimens.

The following anatomical abbreviations were used in the text and figures: AC: aciniform gland spigot; AER: anterior eye row; AL: anterior lobe [applied to epigynum of *Loureedia* gen. n.]; ALS: anterior lateral spinneret; ALE: anterior lateral eye; AME: anterior median eye; BH: basal haematodocha; C: conductor; E: embolus; MAP: major ampullate gland spigot; mAP: minor ampullate gland spigot; MH: median haematodocha; ML: median lobe (of epigynum); MS: modified spigot; PER: posterior eye row; PI: piriform gland spigot; PLS: posterior lateral spinneret; PLE: posterior lateral eye; PME: posterior median eye; PMS: posterior median spinneret; S: spermatheca; SH: spermathecal head; ST: subtegulum; T: tegulum. References to figures published elsewhere are listed in lowercase type (fig.); references to figures in this paper are listed with an initial capital (Fig.).

Institutional abbreviations are as follows: CAS: California Academy of Sciences (San Francisco); TMSA: Ditsong National Museum of Natural History [formerly the Transvaal Museum] (Pretoria); HUJ: Hebrew University of Jerusalem; SAM: Iziko South African Museum (Cape Town); MHNG: Musée d’Histoire Naturelle (Genève); MNHN: Muséum National d’Histoire Naturelle (Paris); ZMHB: Museum für Naturkunde der Humboldt Universität Berlin; NCA: National Collection of Arachnida, ARC-Plant Protection Research Institute (Pretoria); BMSA: National Museum Bloemfontein; NMN: National Museum of Namibia (Windhoek); BMNH: The Natural History Museum (London); NMW: Naturhistorisches Museum Wien (Vienna); RMNH: Netherlands Centre for Biodiversity Naturalis (Leiden); ZMUC: Zoological Museum, University of Copenhagen. Some specimens used for this research are deposited in the personal collection of Milan Řezáč (MR). Specimen record codes often incorporate collection information, but this is not always the case and can be misleading. For this reason, specimens are referenced by both the record code (if available) and collection abbreviation.

### Molecular methods

PCR products were generated either in the DNA Markerpoint Lab at the University of Leiden using standard methods (see Miller et al. 2010a) or the NCB Naturalis DNA Barcoding Facility. Sequencing was performed by Macrogen (http://www.macrogen.com). DNA sequence data was added to the manual alignment described in Miller et al. (2010a). GenBank accession numbers linked to online records for all new sequence data generated for this study are given in Table 1.

The data were analyzed using MrBayes version 3.1 (Huelsenbeck and Ronquist 2001; Ronquist and Huelsenbeck 2003) under the conditions described in Miller et al. (2010a, i.e., mixed model analysis with eight data partitions, gaps treated as missing) on the Cyberinfrastructure for Phylogenetic Research (CIPRES) portal (http://www.phylo.org/). Analysis proceeded until the standard deviation of split frequencies fell below 0.01 (after approximately 13,500,000 of 25,000,000 generations). The first 10% of generations was discarded as burnin based on evaluation in Tracer version 1.5 (Rambaut and Drummond 2007).

**Table 1.** Genbank accession numbers for new sequences generated for this study. Codes identify individual specimens and are kept as labels with vouchers.

**Taxon**	**Code**	**Sex**	**Brief location**	**COI**	**28S**
*Gandanameno* sp.	18-01	Female	South Africa: Western Cape: De Hoop Nat Reserve, Potberg	JQ026497	
*Gandanameno* sp.	18-04	Female	South Africa: Western Cape: Swartberg, Nat. Res. Gamkaskloof	JQ026498	
*Gandanameno* sp.	18-05	Male	Zimbabwe: Harare, 19 Walmer Drive	JQ026499	
*Gandanameno* sp.	18-06	Male	South Africa: Western Cape: Farm Tierberg, NE of Prince Albert	JQ026500	
*Gandanameno* sp.	18-08	Male	South Africa: Western Cape: Farm Tierberg, NE of Prince Albert	JQ026501	
*Gandanameno* sp.	19-03	Female	South Africa: Free State Province: Bloemfontein	JQ026502	
*Gandanameno* sp.	19-06	Female	South Africa: Eastern Cape: Willowmore, Uitspan	JQ026503	
*Gandanameno* sp.	19-07	Female	South Africa: Free State Province: Bloemfontein	JQ026504	
*Gandanameno* sp.	20-02	Female	South Africa: Western Cape: Knysna, Southern Comfort	JQ026506	
*Gandanameno* sp.	20-04	Female	South Africa: Gauteng: Rietfontein, Pretoria	JQ026496	
*Gandanameno* sp.	20-05	Male	South Africa: Eastern Cape: Uitenhage, Springs Resort	JQ026505	
*Gandanameno* sp.	RMNH.ARA.14513	Male	South Africa: Western Cape: Vanrhynsdorp	JQ026507	
*Gandanameno* sp.	RMNH.ARA.14514	Male	South Africa: Western Cape: route N7	JQ026508	
*Gandanameno* sp.	RMNH.ARA.14515	Male	South Africa: Western Cape: Anysberg Nature Reserve	JQ026509	
*Gandanameno* sp.	RMNH.ARA.14516	Male	South Africa: Western Cape: Cape Town	JQ026510	
*Gandanameno* sp.	RMNH.ARA.14517	Female	South Africa: Western Cape: route N7	JQ026511	
*Gandanameno* sp.	RMNH.ARA.14518	Male	South Africa: Western Cape: route N7	JQ026512	
*Gandanameno* sp.	RMNH.ARA.14519	Female	South Africa: Western Cape: Anysberg Nature Reserve	JQ026513	
*Gandanameno* sp.	RMNH.ARA.14520	Female	South Africa: Western Cape: Cape Town surroundings	JQ026514	
*Gandanameno* sp.	RMNH.ARA.14521	Female	Namibia: Homeb	JQ026515	
*Paradonea variegata*	RMNH.ARA.14512	Male	Namibia: app. 50 km SW Aus (on road C13)	JQ026516	JQ026518
*Paradonea variegata*	RMNH.ARA.14522	Male	Namibia: app. 50 km SW Aus (on road C13)	JQ026517	

## Data resources

We used the Pensoft IPT Data Hosting Center to expose specimen occurrence records to the Global Biodiversity Information Facility (GBIF; http://ipt.pensoft.net/ipt/resource.do?r=specimen_occurrence_data). A KML (Keyhole Markup Language) file for viewing these same specimen occurrence records interactively in Google Earth (http://earth.google.com/) plus links to species pages on the Encyclopedia of Life (http://www.eol.org/) is available as part of a Dryad data package (http://dx.doi.org/10.5061/dryad.qj8t7r0q).

The alignment of the molecular sequence data used for the phylogenetic analysis is available on Dryad (http://dx.doi.org/10.5061/dryad.qj8t7r0q). Figures showing the full phylogenetic tree (Fig. S1) and images of some specimens newly sequenced for this study (Figs S2, S3) are available as an electronic document via Dryad (http://dx.doi.org/10.5061/dryad.qj8t7r0q).

## Systematics

### Eresidae

C. L. Koch

http://species-id.net/wiki/Eresidae

Chercheuses (Erraticae) Walckenaer, 1802: 248; Walckenaer 1805: 21.
Eresidae C. L. Koch, 1850: 70; Simon 1892: 248–254; Lehtinen 1967: 385–390; Griswold et al. 2005: 24–27.

#### Nomenclatural note.

Walckenaer (1802) divided spiders into 18 “Famillies.” At this time, concepts of nomenclature were distinctly different from our modern understanding. All spider species were referred to by a binomen with “Aranea” as the genus regardless of “famillie” placement. Chercheuses (Erraticae) contained one species: *Aranea cinnaberina* (now *Eresus kollari*). Because Walckenaer’s family name was not formed from the stem name of the type genus, it was not considered valid once international codes of nomenclature were adopted starting in 1905 (International Commission on Zoological Nomenclature 1905). For an enlightening discussion of Walckenaer’s and related systems of spider classification, see Edwards (2011).

#### Diagnosis.

Distinguished from other three-clawed, eight-eyed, cribellate entelegyne spiders except Penestomidae by their subrectangular carapace and clypeal hood; distinguished from Penestomidae by the absence of an RTA on the male palpal tibia, the absence of a median apophysis arising from the palpal tegulum, the absence of a posterior lobe of the epigynum (the posterior lobe is a separate plate in Penestomidae; compare Figs 45A, 93A with Miller et al. 2010b: fig. 8A), and the absence of a tapetum in the indirect eyes. The eye arrangement in Eresidae is distinctive, with a straight anterior eye row and strongly recurved posterior eye row, with the median eyes close together, the ALE near the anterior lateral corners of the carapace, and the PLE position on the carapace at least 0.2 (Figs 8B, D, F, H, J, L, 9B, D, F, H, J, L, 10 B, D, F, H, J, L, 11B, D, F, H, J, L); by contrast, Penestomidae have the anterior eyes subequally spaced with the ALE placed about midway between the center and the corners of the carapace (Miller et al. 2010b: fig. 1C), and the PLE position on the carapace ca. 0.1.

#### Description.

*Somatic morphology*:Carapace subrectangular in dorsal view; cephalic region may be strongly raised. Eight eyes in two rows, posterior eye row strongly recurved so that the PLE are set far back from the others (Figs 8B, D, F, H, J, L, 9B, D, F, H, J, L, 10 B, D, F, H, J, L, 11B, D, F, H, J, L). Tapetum absent from eyes. The anterior-median part of carapace extended ventrally into a clypeal hood (Figs 8A, C, E, G, I, K, 9A, C, E, G, I, K, 10A, C, E, G, I, K, 11A, C, E, G, I, K). Two or more setal morphologies typically present appearing as dark and white setae in museum specimens (Figs 35B, 81D, 91B). Chelicerae robust, may be contiguous (Fig. 19G) or excavated mesally (Fig. 68F), distal anterior part with dense cluster of strong setae near fang (Fig. 28C), usually with boss (Figs 28B, 46B, 56E, but see 56C); large single keel anterior to fang, may be serrate, with series of small denticles leading towards base of fang; there is no distinct fang furrow (Figs 34F, 91F). Female palp with tarsal claw (Fig. 92F). Legs usually short with two rows of trichobothria on tibiae and one distal trichobothrium on metatarsi. Bothria have series of transverse grooves proximally (Figs 25D, 38B, 45E, 92B). Tarsal organ small, capsulate, and positioned near the distal tip (Figs 38A, 46D, 92A). Major and median claws with series of teeth (Figs 38C, 92C). Linear calamistrum occupies entire length of metatarsus IV (Figs 25E, 38D, 46E, 92D), with a dorsal patch of smaller calamistral setae (i.e., with lines of teeth, Figs 25F, 31F, 38E, 46F, 67F, 82F, 92E). In some eresids, the line of primary calamistrum setae is not clearly distinguishable from the dorsal patch (Figs 25F, 31F, 46F, 67F). Abdomen generally oblong with distinct dorsal sigilla (Figs 3I, 4H, 19A, 47I, 89E). Posterior respiratory system comprises four simple tracheal tubes (Lamy 1902; CEG, pers. obs. *Stegodyphus* and *Dresserus*).

**Figure 12.**
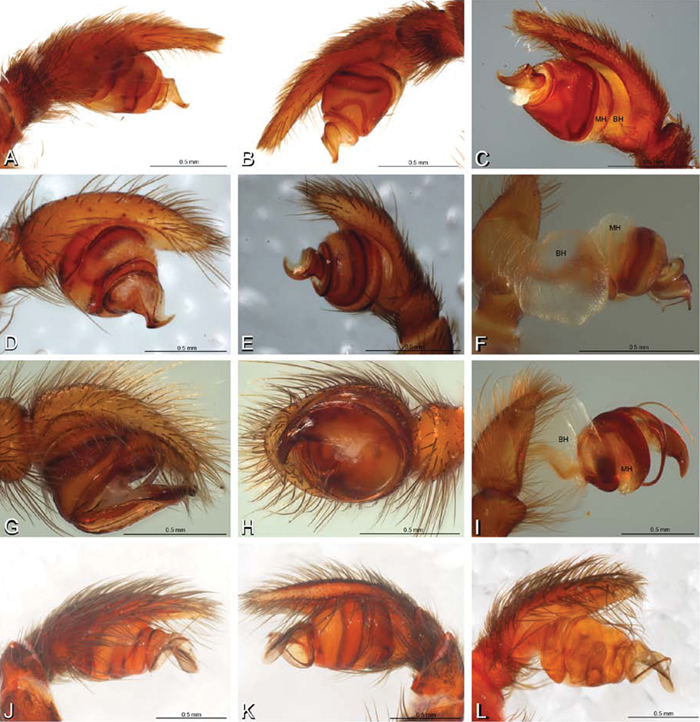
**A–L** Left male palpi of eresid species, photomicrographs. **A–C** *Adonea fimbriata* from Algeria-Morocco (MR012, MR) **D–F** *Dorceus fastuosus* from Mashabin Sand Dunes, Israel (MR006, HUJ) **G–I** *Dresserus* sp. from Manga Forest Reserve, Tanzania **J–L** *Eresus walckenaeri* from Leptokaryas, Greece (MR020, MR) **A, D, G, J** prolateral view **B, E, K** retrolateral view **H** ventral view **C, F, I, L** expanded palp. **BH** basal haematodocha **MH** median haematodocha.

**Figure 13.**
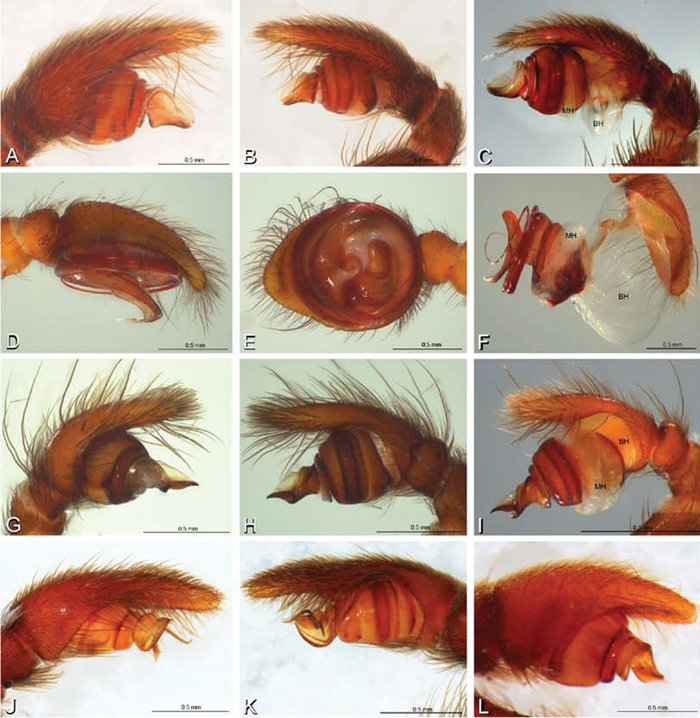
**A–L** Left male palpi of eresid species, photomicrographs. **A–C** *Eresus kollari* from res. Radotinske udoli, Czechia (MR007, MR) **D–F** *Gandanameno* sp. from Van Riebeeck Park, Western Cape, South Africa (CASENT 9023763, CAS) **G–I** *Loureedia annulipes* from Haluqim Ridge, Israel (PET03, MR) **J, K** *Paradonea striatipes* from Otjivasandu (NMN), Namibia **L** *Paradonea splendens* from Sunnyside, South Africa (C1076, SAM) **A, D, G, J, L** prolateral view **B, H, K** retrolateral view **E** ventral view **C, F, I** expanded palp. **BH** basal haematodocha **MH** median haematodocha.

**Figure 14.**
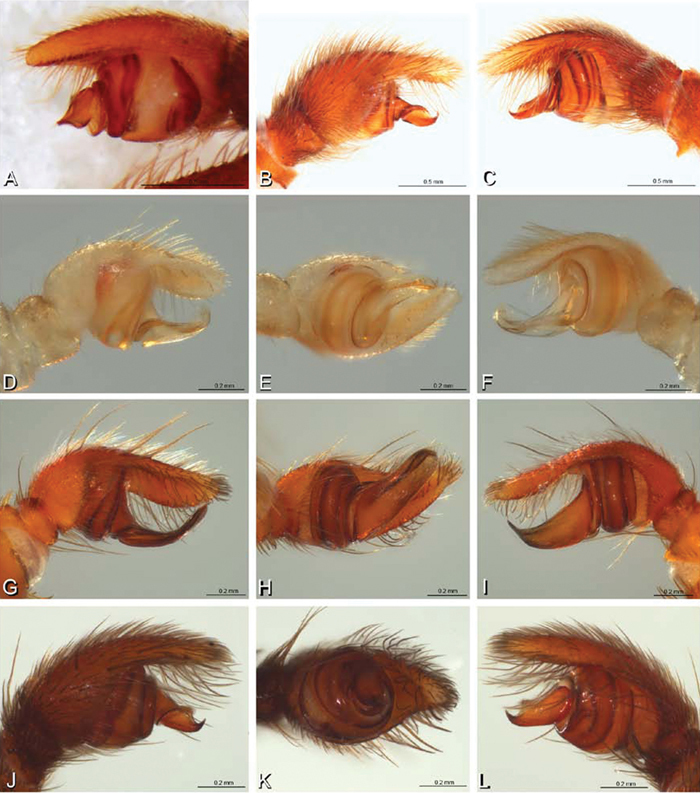
**A–L** Left male palpi of *Paradonea* species, photomicrographs. **A** *Paradonea splendens* from Sunnyside, South Africa (C1076, SAM) **B, C** *Paradonea variegata* from Breekkierie Dunes, Northern Cape, South Africa (C1062, SAM) **D–I** *Paradonea parva* **D–F** holotype from junction of Marico and Crocodile Rivers, South Africa (B3701, SAM) **G–I** from 4 km N of Hopetown, Northern Cape, South Africa (AcAT 97/988, NCA) **J–L** *Paradonea presleyi* sp. n. holotype from Falcon College, Zimbabwe (CASENT 9039236, CAS) **A, C, F, I, L** retrolateral view **B, D, G, J** prolateral view **E, H, K** ventral view.

**Figure 15.**
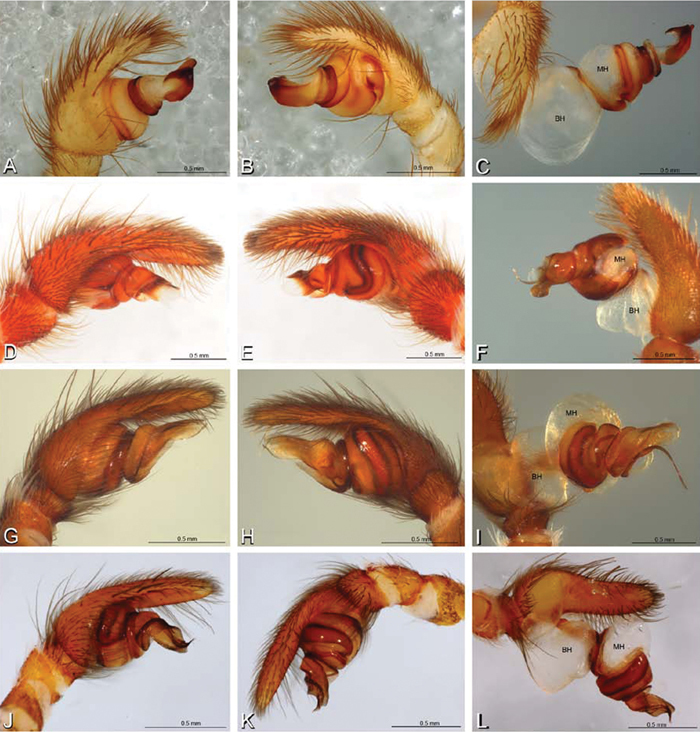
**A–L** Left male palpi of eresid species, photomicrographs. **A–C** *Seothyra henscheli* from Gobabeb Station, Namibia (SMN 40828, NMN) **D, F** *Stegodyphus lineatus* **D–E** from Negev, Israel (MR) **F** from Nengrahar, Afghanistan (MR010, MR) **G–I** *Stegodyphus mimosarum* from Forêt d'Analalava, Fianarantsoa, Madagascar (CASENT 9015950, CAS) **J–L** *Stegodyphus sarasinorum* from 7.5 km E PwintPhyu, Magway Division, Myanmar (CASENT 9019370, CAS) **A, D, G, J** prolateral view **B, E, H, K** retrolateral view **C, F, I, L** expanded palp. **BH** basal haematodocha **MH** median haematodocha.

*Male palp*: Male palpal tibia without apophysis, with two rows of trichobothria (Figs 27F, 34E, 55F). Palpal bulb with sclerotized conductor that interacts with spiral embolus (Figs 27C, 34D, 41F, 90D), expansion occurs in both the basal and median haematodochae (Figs 12F, 13C, F, 15C, L). Axis of spiral typically proximal-distal with embolus encircling distal part (Figs 12B, 13B, H, J, 14I, 15B, H, K), occasionally more or less ventral-dorsal with embolus encircling ventral part (*Dresserus* and *Gandanameno*; Figs 12G, 13D, 33I–K, 48A–F).

**Figure 16.**
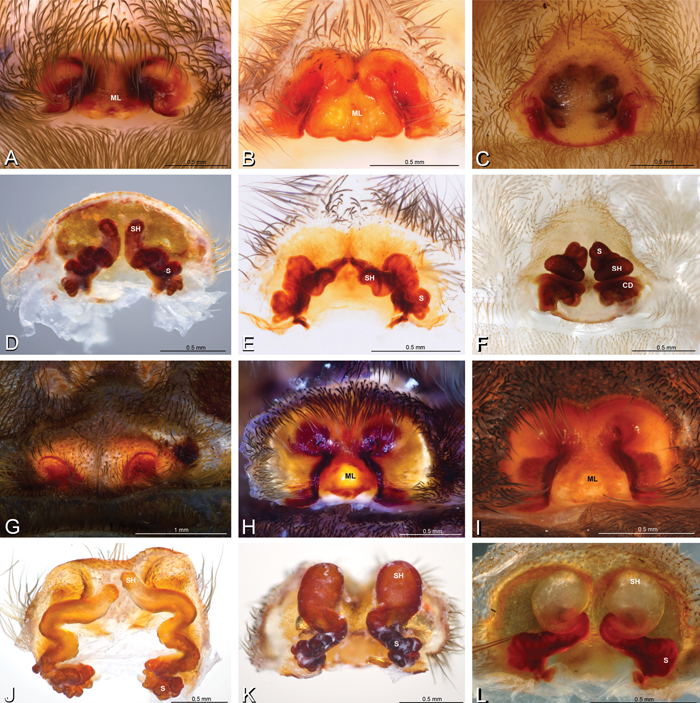
**A–L** Epigyna of eresid species, photomicrographs. **A, D** *Adonea fimbriata*; A from Mehav Am village, Israel (MR003, MR) **D** from Wadi Mashash, Israel (MR013, HUJ) **B, E** *Dorceus fastuosus* from Mashabim sand dunes, Israel (MR002, MR) **C, F** *Dresserus* sp. from Klein Kariba, South Africa (CASENT 9025745, CAS) **G, J** *Eresus walckenaeri* from 5 km south of Monemvasia, Lakonia, Greece (ZMUC 00012903, ZMUC) **H, K** *Eresus kollari* from res. Radotinske udoli, Czechia (MR016, MR) **I, L** *Eresus sandaliatus* from SE of Silkeborg, Denmark (CASENT 9039243, CAS) **A–C, G–I** ventral view**D–F, J–L** dorsal view, cleared. **CD** copulatory duct **ML** median lobe **S** spermatheca **SH** spermathecal head.

*Female genitalia*: Epigynum present with entelegyne configuration, one pair of spermathecae (typically in a posterior position except in *Dresserus* and *Gandanameno*, where they are anterior), and spermathecal heads (typically in an anterior position and far from the spermathecae except in *Dresserus* and *Gandanameno*, where they are adjacent to the spermathecae; Figs 16D–F, J–L, 17D–F, 18D–F, J–L, 22B, 29D, 37E, 42D, 45B, 59C, 65B, 76B, 82B, 86B, 93B); posterior lobe absent (compare Figs 45A, 93A with Miller et al. 2010b: fig. 8A).

**Figure 17.**
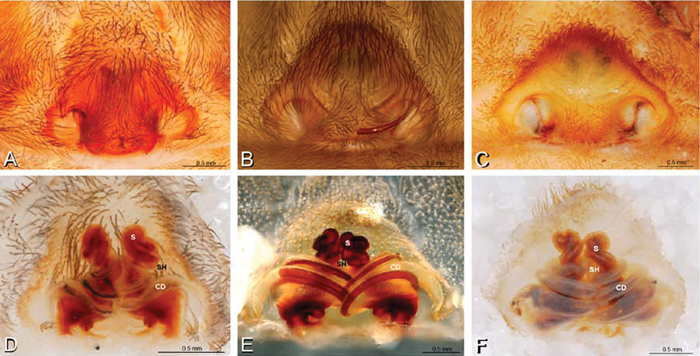
**A–F** Epigyna of *Gandanameno* sp., photomicrographs. **A, D** from Iringa, Tanzania (ZMUC 19970517, ZMUC) **B, E** from Kommetjie, Western Cape, South Africa (CASENT 9039241, CAS), note broken embolus left in female reproductive system **C, F** from Port Elizabeth, South Africa (port-3325, ZMHB) **A–C** ventral view **D–F** dorsal view, cleared. **CD** copulatory duct **S** spermatheca **SH** spermathecal head.

**Figure 18.**
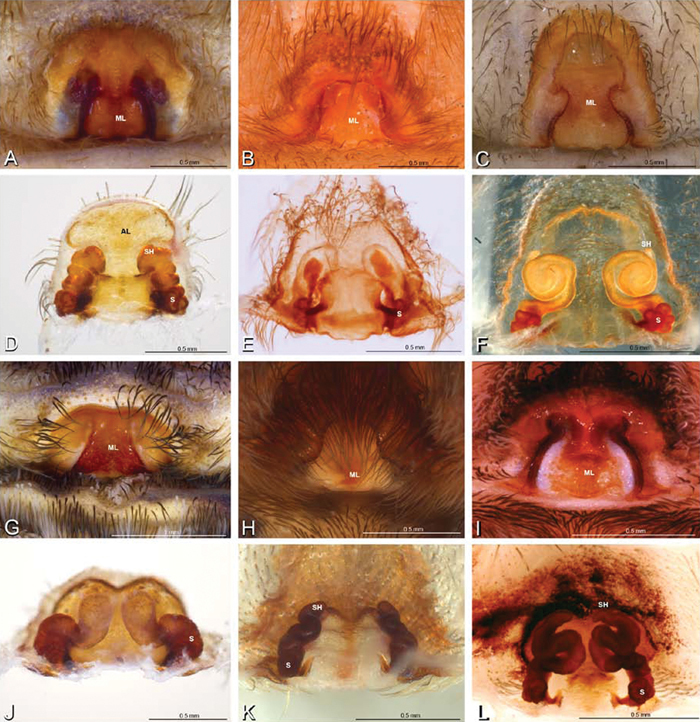
**A–L** Epigyna of eresid species, photomicrographs. **A, D** *Loureedia annulipes* from Wadi Mashash, Negev, Israel (MR019, MR) **B, E** *Paradonea variegata* from Steinkopf, Northern Cape, South Africa (ZMB 26964, ZMHB) **C, F** *Seothyra henscheli*; C from Kuiseb River, Gobabeb, Namibia (SMN 46627, NMN) **F** from Sout Rivier, Namibia (CASENT 9039242, CAS) **G, J** *Stegodyphus lineatus* from Belkis, near Birecor, Turkey (MR015, MR) **H, K** *Stegodyphus mimosarum* **H** from Forêt d'Analalava, Fianarantsoa, Madagascar (CASENT 9015950, CAS) **K** from Réserve Spéciale de Cap Sainte Marie, Toliara, Madagascar (CASENT 9012844, CAS) **I, L** *Stegodyphus sarasinorum* from 7.5 km E PwintPhyu, Magway Division, Myanmar (CASENT 9019370, CAS) **A–C, G–I** ventral view **D–F, J–L** dorsal view, cleared. **AL** anterior lobe **ML** median lobe **S** spermatheca **SH** spermathecal head.

*Spinneret spigot morphology*: ALS typically with multiple MAP (absent in *Seothyra*, Figs 77B, 78B) and a field of PI (Figs 36B, 94B). PMS with one to several mAP and a field of AC, occasionally elongated and divided into two lobes (female *Dresserus* and *Gandanameno*, Figs 36C, 57A, C), CY present (Figs 36C–D, 57C, 58E) or uncertain. PLS with field of AC, MS positioned on dorsal part adjacent to ALS far from rest of field, may be accompanied by one (*Dorceus*, Figs 30F, 32F) or two (*Eresus sandaliatus* group, *Loureedia* gen. n., *Seothyra*, *Stegodyphus*, Figs 67D, 87D, 95E) flanking AC (no MS-flanking AC in at least *Dresserus* and *Gandanameno*, Figs 36E, 57D, 58C, 61B–C). Cribellum present with median division in most genera (Figs 57E, 77E, 87A, 94A, E), each half subdivided in *Dresserus* (Fig. 36F). Multiple epiandrous gland spigots present in male (Figs 22E–F, 28D, 39F, 45F, 61E–F, 65F, 74E–F, 80E–F, 85E–F, 93F).

#### Phylogeny.

Our phylogenetic analysis is a modest expansion of Miller et al. (2010a) and the topology is congruent with the earlier study. The additions to the new analysis are two specimens of *Paradonea variegata* and twenty more specimens of *Gandanameno*. As reported previously, Eresidae is divided into two major clades: one consisting of *Seothyra*, *Dresserus*, and *Gandanameno*, the other containing the remaining genera including *Paradonea* (Figs 51, S1). In our topology, *Paradonea* sits on a long branch sister to a clade consisting of *Eresus*, *Adonea*, *Loureedia* gen. n., and *Dorceus*; *Stegodyphus* is sister to this five-genus clade. Note that our exemplar for *Paradonea* is not the type species and the monophyly of this genus is uncertain. Our focus on sequencing *Gandanameno* was designed to elucidate species limits within the genus, in combination with morphological data (Figs 50, S2, S3). These results are discussed further in the section on *Gandanameno*, below.

#### Key to genera of Eresidae

(note: females of *Paradonea striatipes* Lawrence, 1968, *Paradonea splendens* (Lawrence, 1936), *Paradonea parva* (Tucker, 1920), and *Paradonea presleyi* sp. n. are unknown)

**Table:** 

1a	Median eyes small, subequal in size, and no more than slightly overlapping on vertical axis (Figs 8E, G, 10I, K). ALS enlarged, extensible, PLS reduced (Figs 32A, 72D, H, 77A, 78A)	2
1b	PME larger than AME (Fig. 8A) or if nearly equal in size then significantly overlapping on vertical axis (Fig. 11A). ALS no more than slightly longer than PLS (Figs 36A, 57A, 94A)	3
2a	Cephalic region longer than wide (Figs 10J, 72A, E). Male leg I enlarged (Figs 72A, 74A). Palpal conductor highly variable and elaborate, usually longer than tegular division (Figs 15B, 72J, 73A, B; see also Dippenaar-Schoeman 1990). Median lobe of epigynum clearly longer than wide with a central constriction (Figs 18C, 76A)	*Seothyra*
2b	Cephalic region wider than long (Figs 8F, H, 26A, E, 29B). Male legs I and II subequal (Fig. 26A). Palpal conductor a simple spiral, shorter than tegular division (Figs 26I, 27A). Median lobe of epigynum wider than long with more or less straight, converging lateral margins (Figs 16B, 29C)	*Dorceus*
3a	Median eyes separated on horizontal axis by ca. half of one AME diameter or more (Figs 10A, 11A)	4
3b	Median eyes no more than slightly separated on horizontal axis (Figs 9A, I, 10G)	7
4a	PME position ca. 0.44, cephalic region trapezoidal, much wider anteriorly than posteriorly (Figs 10D, 68D) with straight posterior margin that overhangs thoracic region	*Paradonea splendens*
4b	PME position < 0.3, cephalic region subtriangular with round posterior margin (Figs 68A, 79A, E) that does not overhang thoracic region	5
5a	Median eyes not overlapping on vertical axis, ALE much smaller than PLE (ALE/PLE ca. 0.3; Figs 10A, 68C)	*Paradonea striatipes*
5b	Median eyes significantly overlapping on vertical axis, ALE more than half the diameter of the PLE (Fig. 11E)	6
6a	Clypeal hood long, acute (Figs 11E, 81A)	*Stegodyphus*
6b	Clypeal hood short, ca. 90° (Fig. 70I)	*Paradonea presleyi* sp. n.
7a	PLE position < 0.28. Male palp with dorsal-ventral axis with embolus encircling ventral part (Figs 12G, 13D, 33I–K, 48A–F). Epigynum with wide atria separated by hirsute cuticle (Figs 17A–C, 59A). Copulatory ducts coil at least once around fertilization ducts, spermathecal head adjacent to spermathecae (Figs 17D–F, 59C). Female PMS subdivided (Figs 36C, 57A, C)	8
7b	PLE position > 0.31. Male palp with proximal-distal axis with embolus encircling distal part (Figs 12B, 13B, H, J, 14I, 15B, H, K). Epigynum usually with slit-like atria separated by glabrous median lobe (Figs 29C, 45A, 65A, 76A, 93A), if separated by hirsute cuticle (Figs 16G, 42B), then copulatory ducts sinuous and spermathecal head separated from spermathecae (Figs 16J, 42D). Female PMS entire (Figs 66C, 94C)	9
8a	Male with ALE on pointed apophyses (Fig. 8J). Embolus encircles palp for less than 1.5 turns (Fig. 34A–D). Copulatory duct makes ca. 1 turn around fertilization ducts (Figs 16F, 37E). Cribellum divided into four parts (Fig. 36F)	*Dresserus*
8b	Male ALE not on pointed apophyses (Fig. 9F). Embolus encircles palp ca. 3 turns (Figs 48, 55A–D). Copulatory duct makes ca. 3 turns around fertilization duct (Figs 17D–F, 59C). Cribellum usually divided into two parts, occasionally signs of four part cribellum evident (Fig. 57E)	*Gandanameno*
9a	Cephalic region wider than long (Figs 9J, L, 62A, E). Palpal conductor with bifid process (Fig. 63D, E). Vulva with compact duct system and anterior lobe (Figs 18D, 65B)	*Loureedia* gen. n.
9b	Cephalic region longer than wide. Palpal conductor without bifid process. Vulva variable, without anterior lobe	10
10a	Clypeal hood ca 90° (Figs 69C, F, 70F, I)	11
10b	Clypeal hood forms clearly acute angle (Figs 8C, 9C)	12
11a	Male chelicerae strongly excavated mesally (Fig. 69C). Embolic division of male palp shorter than tegular division (Figs 14B, C, 69G, H). Epigynum as in Fig. 18B	*Paradonea variegata* (Purcell, 1904)
11b	Chelicerae only slightly excavated mesally (Fig. 70C, F). Embolic division of male palp longer than tegular division (Fig. 14D–I). Female unknown	*Paradonea parva*
12a	Male with cephalic region overhanging thoracic region posteriorly (Fig. 19D) and dorsal surface of abdomen dark gray, nearly encircled by a band of white setae, with numerous patches of white setae dorsally, especially around sigilla (Fig. 19A). Female with cephalic region strongly raised so posterior margin is nearly vertical (Figs 1A, 19H)	*Adonea*
12b	Male with cephalic region not overhanging thoracic region posteriorly and dorsal surface of abdomen usually with two pairs of large round dark patches surrounding the first and second sigilla on a field of red setae (Figs 2B, D, 40A, 43A), occasionally all black. Female with cephalic region only moderately raised (Figs 40H, 43H)	*Eresus*

### Adonea

Simon

http://species-id.net/wiki/Adonea

Adonea Simon 1873: 157. Type species *Adonea fimbriata* Simon, 1873.
Storkaniella Kratochvíl and Miller 1940: 93, figs 2, 4. Synonymy in Lehtinen 1967: 208, 265.

#### Note.

*Adonea* contains one recognized species, *Adonea fimbriata* Simon, 1873, from the Mediterranean. In addition, *Eresus algericus* El-Hennawy, 2004 is transferred to *Adonea* and may be a junior synonym of *Adonea fimbriata*. We examined syntype specimens from Algeria and Tunisia, and additional specimens from the Algeria-Morocco border and Israel.

#### Diagnosis.

**Figure 19.**
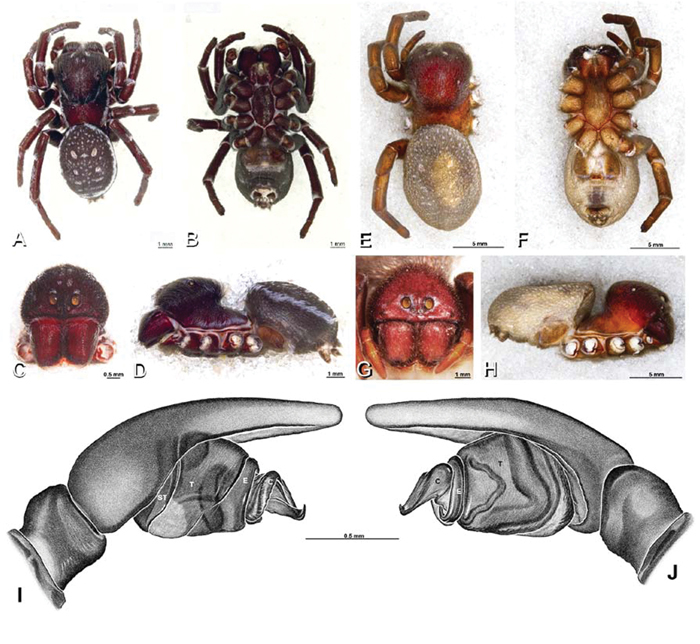
**A–J** *Adonea fimbriata*. **A–D, I–J** male from Algeria-Morocco (MR012, MR) **E–H** female from Mehav Am village, Israel (MR003, MR) **A–D** habitus of male, photomicrographs **E–H** habitus of female photomicrographs **I, J** illustrations of left male palp **A, E** dorsal view **B, F** ventral view **C, G** anterior view. **D, H** lateral view **I** prolateral view **J** retrolateral view. **C** conductor **E** embolus **ST** subtegulum **T** tegulum.

Male distinguished from other eresids except *Paradonea splendens* by the profile of the carapace, which has the posterior part of the cephalic region overhanging the anterior part of the thoracic region (Fig. 19D); distinguished from *Paradonea splendens* by several characters including the subtriangular shape of the cephalic region that is rounded posteriorly (Fig. 19A; trapezoidal in *Paradonea splendens* and straight posteriorly, Fig. 68D) and by the mesally contiguous chelicerae (Fig. 19C; mesally excavated in *Paradonea splendens*, Fig. 68F).

Female distinguished from other eresids except *Loureedia* gen. n., *Eresus walckenaeri* Brullé, 1832, and some *Paradonea* species by the relatively large PME (AME/PME ca. 0.4, Fig. 19G); distinguished from *Loureedia* gen. n. by the longer than wide cephalic region (wider than long in *Loureedia* gen. n.); from *Eresus walckenaeri* by the presence of a glabrous median lobe between the copulatory openings (Fig. 22A; hirsute cuticle between the copulatory openings in *Eresus walckenaeri*, Fig. 42B); and from *Paradonea variegata* by the nearly vertical posterior margin of the cephalic region (Figs 1A, 19H; cephalic region only moderately raised in *Paradonea variegata*); females of other *Paradonea* species are unknown. The proportions of the epigynum in *Adonea*, which is more than two times wider than long, further separates it from most eresids (Figs 16A, 22A).

#### Natural history.

Known from Loess desert habitat with low shrubs, often in wadis. They build a simple vertical or inclined burrow lined by silk, often on the edge of stones. The opening is covered by a silken flap camouflaged from above by debris. Signaling threads radiate out from the edges of this roof. Prey include various epigaeic arthropods, especially beetles from the family Tenebrionidae. Prey remnants are incorporated into the roof of the burrow. Males take approximately 2–3 years to mature, females one year longer (Martin Forman, personal observation).

### Adonea fimbriata

Simon

http://species-id.net/wiki/Adonea_fimbriata

Figs 1A, B 4A 8A–D 12A–C 16A, D 19 [Fig F20] [Fig F21] [Fig F22] [Fig F23] [Fig F24] –25

Adonea fimbriata Simon 1873: 158, pl. 3, figs 24–25. Simon 1892: 253, figs 202, 207. Kratochvíl and Miller 1940: 92, figs 1, 3. Lehtinen 1967: 208.
Adonea capitata Simon 1876: LXXXVI [86]. Synonymy in Lehtinen 1967: 208.
Storkaniella janinensis Kratochvíl and Miller 1940: 93, figs 2, 4. Synonymy in Lehtinen 1967: 208.

#### Description.

*Male* (Algeria-Morocco, MR012, MR): Carapace with band of white setae around margin of thoracic region and scattered patches elsewhere; cephalic region subtriangular, longer than wide, strongly raised with rounded posterior margin overhanging thoracic region; AME distinctly smaller than PME (AME/PME 0.48), median eyes slightly overlapping on horizontal and vertical axes, PME somewhat sunken into carapace; ALE tubercles present; PER slightly narrower than AER (PER/AER 0.88), PLE position on carapace 0.35; clypeal hood forms acute angle; fovea indistinct. Chelicerae contiguous mesally, with lateral boss. Legs with bands of white setae; with row of distal ventral macrosetae on metatarsus I–IV and scattered short ventral macrosetae on tibia, metatarsus and tarsus I–IV. Abdomen dark gray, nearly encircled by a band of white setae, with numerous patches of white setae dorsally, especially around sigilla (Figs 8A, B, 19A–D).

**Figure 20. F20:**
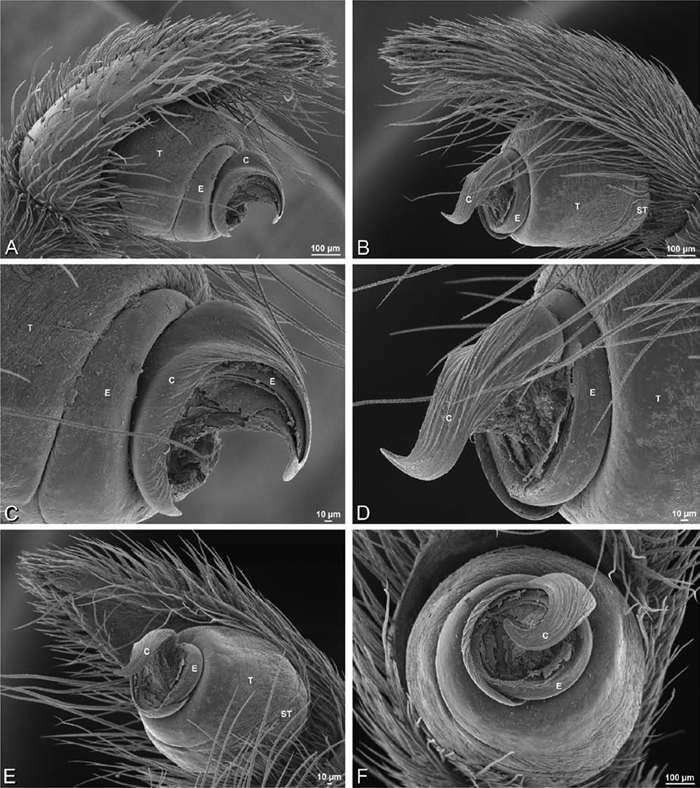
**A–F** *Adonea fimbriata* from Algeria-Morocco (MR012, MR), scanning electron micrographs of right male palp, images reversed to appear as left palp. **A** prolateral view **B** retrolateral view **C** detail of embolic division, prolateral view **D** detail of embolic division, retrolateral view **E** ventral view **F** apical view. **C** conductor **E** embolus **ST** subtegulum **T** tegulum.

Male palp with proximal-distal axis, tegulum moderately elongate, subtrapezoidal; second loop of sperm duct curves proximally away from then back to distal margin of tegulum in retrolateral view (Figs 12B, 19J); conductor and embolus together form apical complex making one helical turn; conductor tapers to point; tegular division longer than embolic division; cymbium with one retrolateral and several prolateral macrosetae (Figs 12A–C, 19I, J, 20A–F).

**Figure 21. F21:**
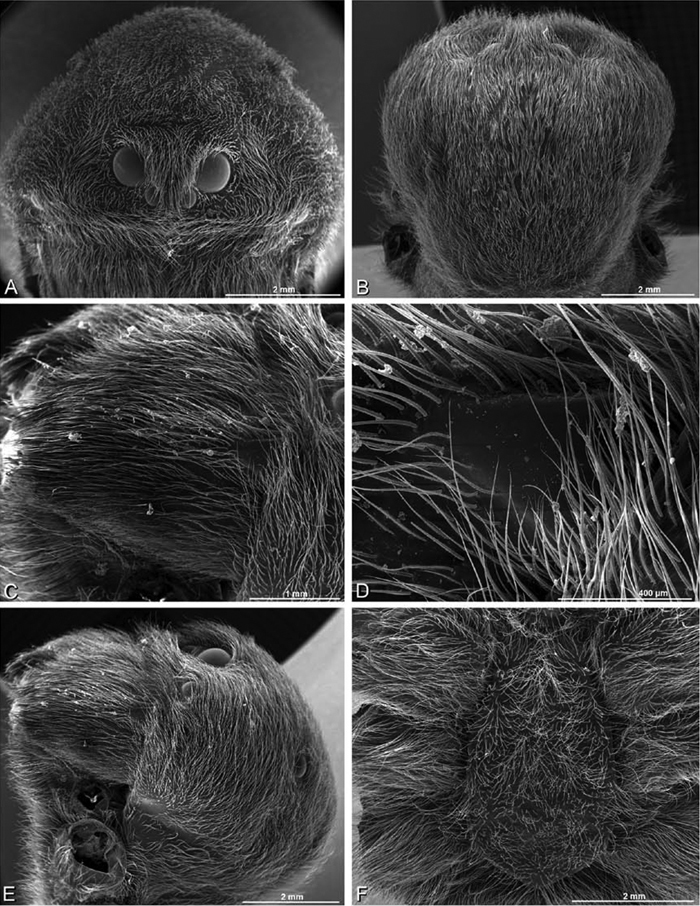
**A–F** *Adonea fimbriata* from Mehav Am village, Israel (MR003, MR), scanning electron micrographs of female prosoma. **A** anterior view **B** dorsal view **C** left chelicerae, lateral view **D** left cheliceral boss **E** lateral view **F** sternum and coxae, ventral view

*Female* (Wadi Mashash, Israel, MR013, HUJ): Carapace with scattered white setae; cephalic region subtriangular, longer than wide, so strongly raised as to be nearly vertical (Figs 1A, 19H); AME distinctly smaller than PME (AME/PME 0.37), median eyes slightly overlapping on horizontal and vertical axes; PME somewhat sunken into carapace; ALE tubercles indistinct; PER slightly narrower than AER (PER/AER 0.82), PLE position on carapace 0.39; clypeal hood forms acute angle; fovea indistinct (Figs 8C, D, 19E-H, 21A, B, E). Chelicerae contiguous mesally, boss present (Figs 19G, 21C, D). Legs with row of distal ventral macrosetae on metatarsus I–IV and scattered short ventral macrosetae on tibia, metatarsus and tarsus I–IV. Abdomen with numerous patches of white setae dorsally, especially around sigilla (Figs 1A, 19E).

**Figure 22. F22:**
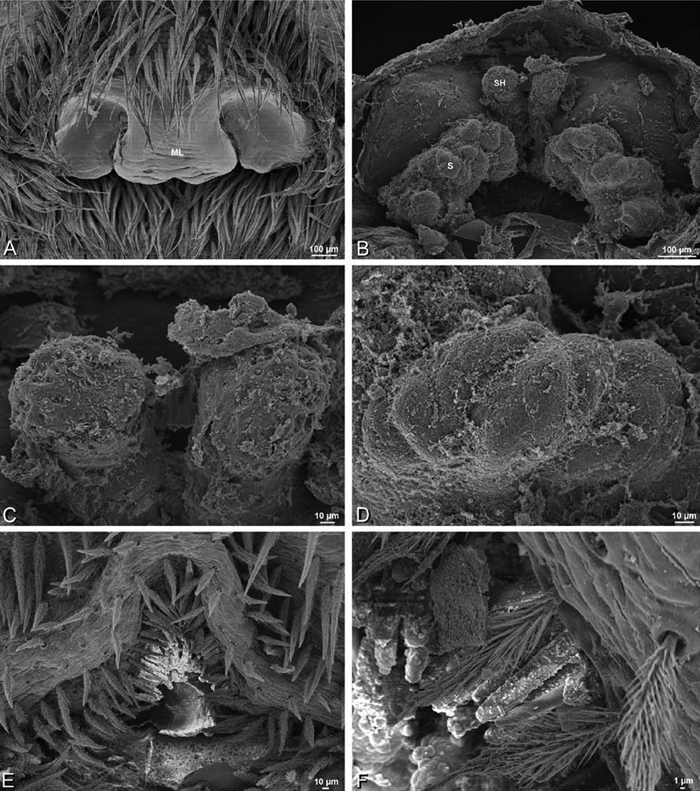
**A–F** *Adonea fimbriata*, scanning electron micrographs. **A** female from Mehav Am village, Israel (MR003, MR) **B–D** female from Wadi Mashash, Israel (MR013, HUJ) **E, F** male from Algeria-Morocco (MR012, MR) **A–D** vulva **E, F** epiandrous region **A** epigynum, ventral view **B** cleared vulva, dorsal view **C** detail, spermathecal heads **D** detail, right spermatheca **E** epiandrous region **F** detail of epiandrous gland spigots. **ML** median lobe **S** spermatheca **SH** spermathecal head.

Epigynum with slightly converging slit-like atria occupying nearly the total length, anterior-lateral margin a curved ridge (Figs 16A, 22A). Vulva with spermathecal heads set anterior-mesally on curved stalks leading to multilobed spermathecae that diverge posteriorly (Figs 16D, 22B–D).

**Figure 23. F23:**
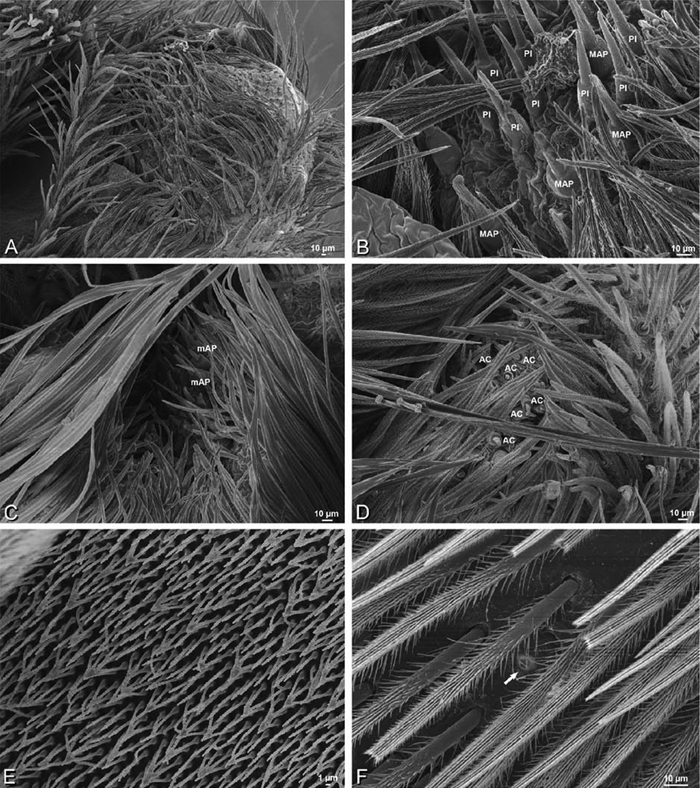
**A–F** *Adonea fimbriata*, female from Mehav Am village, Israel (MR003, MR), scanning electron micrographs of spinnerets. **A** right ALS **B** detail of spigots on right ALS **C** PMS **D** right PLS **E** cribellar spigots **F** arrow indicating tarsal organ, left leg I. Unlabeled spigots in **C** thought to be a mixture of aciniform gland spigots and cylindrical gland spigots. **AC** aciniform gland spigot **MAP** major ampullate gland spigot **mAP** minor ampullate gland spigot **PI** piriform gland spigot.

**Figure 24. F24:**
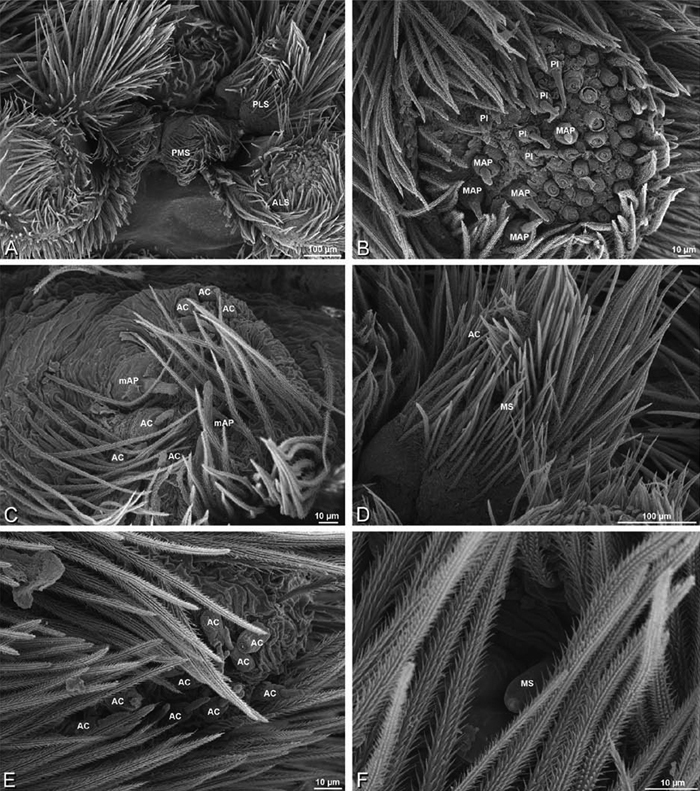
**A–F** *Adonea fimbriata*, male from Algeria-Morocco (MR012, MR), scanning electron micrographs of spinnerets. **A** overview **B** right ALS **C** right PMS **D** right PLS **E** aciniform field on right PLS **F** modified spigot on right PLS. **AC** aciniform gland spigot **ALS** anterior lateral spinneret **MAP** major ampullate gland spigot **mAP** minor ampullate gland spigot **MS** modified spigot **PI** piriform gland spigot **PLS** posterior lateral spinneret **PMS** posterior median spinneret.

**Figure 25.**
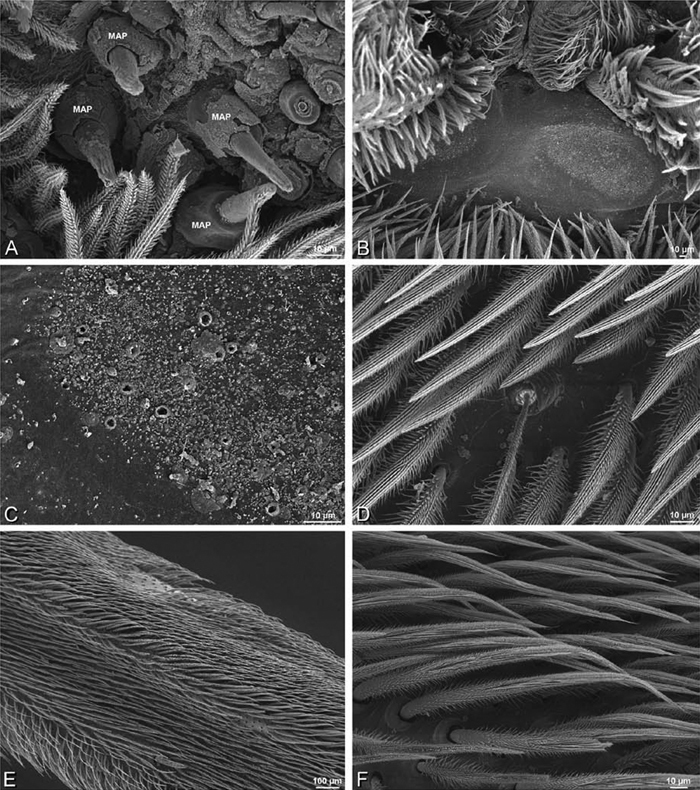
**A–F** *Adonea fimbriata*, scanning electron micrographs. **A–C** male from Algeria-Morocco (MR012, MR) **D–F** female from Mehav Am village, Israel (MR003, MR) **A–C** spinnerets and vestigial cribellum. **D–F** legs of female **A** detail of spigots on right male ALS **B** vestigial cribellum **C** detail of vestigial cribellum **D** trichobothrium, left metatarsus I **E** calamistrum, right metatarsus IV **F** detail, calamistrum seta, right metatarsus IV. **MAP** major ampullate gland spigot.

#### Spinneret spigot morphology

(Mehav Am village, Israel, MR003, MR, and Algeria-Morocco, MR012, MR). Female ALS with at least 5 MAP, at least 1 within and at least 4 on mesal edge of spinning field, with at least 40 PI (Fig. 23A, B); male with fewer PI (Fig. 24B). Female PMS with 2 anterior mAP, posterior field of 21 spigots that vary in size and shape (Fig. 23C); male with only 7 posterior spigots, suggesting that female may have both AC and CY spigots (Fig. 24C). Male PLS with basal MS apparently unaccompanied by flanking AC, distal field of 15 AC (Fig. 24D, F; our female preparation inadequate to view spigots). Male cribellar plate with no sign of spigots (Fig. 25B, C); epiandrous gland spigots present (Fig. 22E, F).

### Dorceus

C. L. Koch

http://species-id.net/wiki/Dorceus

Dorceus C. L. Koch, 1846: 15. C. L. Koch 1850: 70. Simon 1864: 300; 1892: 254; 1910: 290. Lehtinen 1967: 231. El-Hennawy 2002: 58. Type species *Dorceus fastuosus* C. L. Koch, 1846.

#### Note. 

*Dorceus* contains five recognized species previously recorded only from North Africa. The genus was revised by El-Hennawy (2002). We examined specimens of *Dorceus fastuosus* C. L. Koch, 1846 from Israel and Senegal and the Tunisian holotype of *Dorceus viberti* Simon, 1910, which is a junior synonym of this species. The holotype of *Dorceus fastuosus* is a dry pinned specimen and was not examined. But we examined material that El-Hennawy examined for his 2002 revision (9126, AR5404, NMHN and 1237, AR 5405, NMNH) and compared to the holotype.

#### Diagnosis.

**Figure 26.**
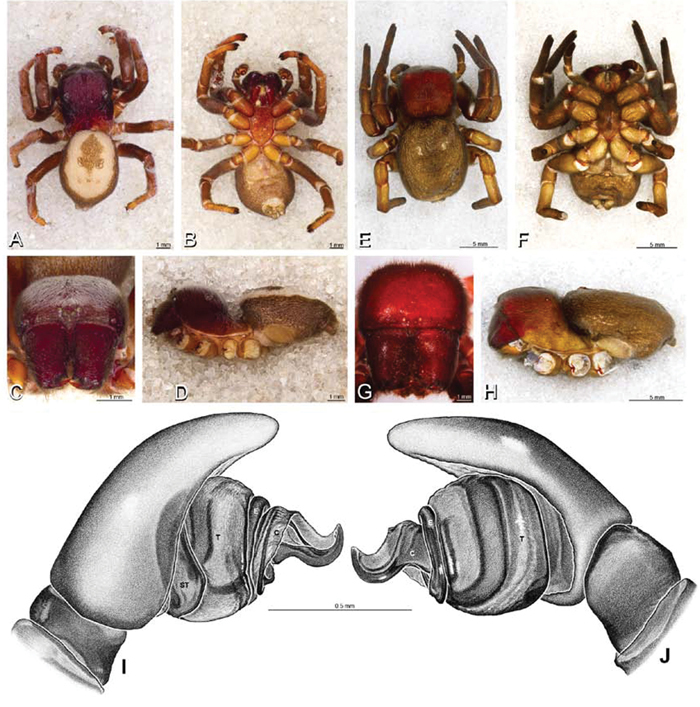
**A–J** *Dorceus fastuosus*. **A–D, I–J** male from Mashabin Sand Dunes, Israel (MR006, HUJ) **E–H** female from Mashabim sand dunes, Israel (MR002, MR) **A–D** habitus of male, photomicrographs **E–H** habitus of female, photomicrographs **J, K** illustrations of left male palp. **A, E** dorsal view **B, F** ventral view **C, G** anterior view **D, H** lateral view. **I** prolateral view. **J** retrolateral view. **C** conductor **E** embolus **ST** subtegulum **T** tegulum.

**Figure 27. F27:**
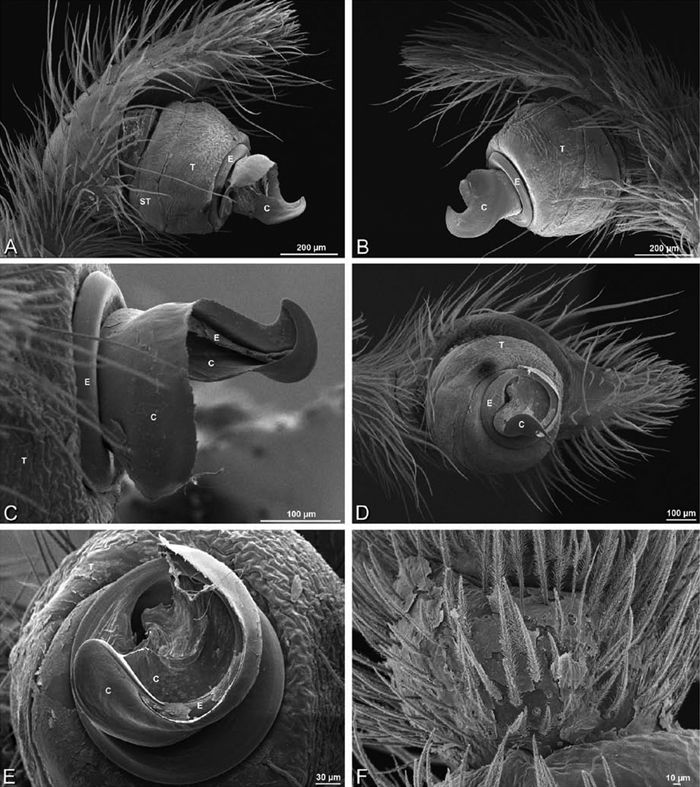
**A–F** *Dorceus fastuosus* from Mashabin Sand Dunes, Israel (MR006, HUJ), scanning electron micrographs of left male palp. **A** prolateral view **B** retrolateral view **C** detail of embolic division, prolateral view **D** ventral view **E** detail of embolic division, ventral view **F** palpal tibia, dorsal view. **C** conductor **E** embolus **ST** subtegulum **T** tegulum.

Distinguished from other eresid genera except *Seothyra* by the small median eyes subequal in size (Fig. 26C, G; AME/PME > 0.7), and the long, extensible ALS contrasting with reduced PLS (Fig. 32A; although ALS may be retracted and therefore not look so long); distinguished from other eresid genera except *Loureedia* gen. n., some *Dresserus*, and *Paradonea splendens* by the cephalic region, which is wider than long. Male distinguished from *Seothyra* by the subequal legs I and II (Fig. 26A; leg I enlarged in *Seothyra*, Figs 72A, 74A) and by the form of the conductor, which is a simple spiral or L-shaped hook shorter than the tegulum (Fig. 27A–C, El-Hennawy 2002; highly variable and elaborate in *Seothyra*, usually longer than the tegulum; see Dippenaar-Schoeman 1990); distinguished from *Loureedia* gen. n. by the unbranched conductor (Fig. 27A–C; bifid in *Loureedia* gen. n., Fig. 63D, E); distinguished from *Dresserus* by the lack of prominent tubercles bearing the ALE and the palpal conformation, which has a proximal-ventral axis with the helical embolus encircling the distal part (Figs 26I, J, 27A–E; obliquely ventral-dorsal in *Dresserus* with the embolus encircling the ventral part, Figs 33I–K, 34A–D); from *Paradonea splendens* by the subrectangular shape of the cephalic region, which does not overhang the thoracic region posteriorly and the mesally contiguous chelicerae (Figs 8F, 26A, C; subtrapezoidal, slightly overhanging the thoracic region, chelicerae mesally excavated in *Paradonea splendens*, Fig. 68D, F). Female distinguished from *Seothyra* by the median lobe of the epigynum, which is as wide as long or wider with more or less straight, converging lateral margins (Figs 16B, 29C; clearly longer than wide with a central constriction in *Seothyra*; see Dippenaar-Schoeman 1990); from *Loureedia* gen. n. by the small eyes subequal in size (Fig. 26C, G) and details of the female genitalia.

#### Distinguishing species.

*Dorceus* was revised by El-Hennawy in 2002. However, the quantity and quality of specimens available to him for several species was limited.

#### Phylogenetic affinities.

Past morphological studies have placed *Dorceus* and *Seothyra* as close relatives (Fig. 8A; Lehtinen 1967). A recent molecular study contradicted this hypothesis (Fig. 7B; Miller et al. 2010a). Although some of us were involved in that molecular study, we do not consider the question resolved. Morphological similarities, including features of the eyes and spinnerets, remain compelling. On the other hand, Peters (1992a) pointed out morphological characteristics shared (through parallel evolution) by *Seothyra* and the distantly related sparasid *Leucorchestris arenicola* exclusive of the eresid *Stegodyphus*, particularly the large ALS, which are extensible and retractable. This morphology is apparently linked to burrowing in loose sand, which *Dorceus* does as well. Whether these attributes ultimately prove to be the result of shared ancestry or convergence in Eresidae remains fertile ground for future study.

#### Natural history.

Known from sand dunes in deserts with very sparse shrub, grass, and annual herb patches. Juveniles feed on their mother’s corpse before dispersing (El-Hennawy 1998; cf. Fig. 3D). Males take approximately 3 years to mature, females one year longer (Martin Forman, personal observation).

### Dorceus fastuosus

C. L. Koch

http://species-id.net/wiki/Dorceus_fastuosus

Figs 8E–H 12D–F 16B, E 26 [Fig F27] [Fig F28] [Fig F29] [Fig F30] [Fig F31] –32

Dorceus fastuosus C. L. Koch, 1846: 15, fig. 1088; Simon 1886: 366; 1892: 254, fig. 205; Lehtinen 1967: 231; El-Hennawy 2002: 61, figs 1, 3–4, 11, 15–20.
Erythrophora fastuosus (C. L. Koch, 1846). Simon 1864: 300.
Dorceus caniceps Simon 1910: 291. Synonymy in El-Hennawy 2002: 61.
Dorceus viberti Simon 1910: 292. Synonymy in Lehtinen 1967: 231.
Dorceus canicipiti Simon 1910: 294 (nomen nudum); Roewer 1954: 1291. Synonymy in El-Hennawy 2002: 62.

#### Description.

**Figure 28. F28:**
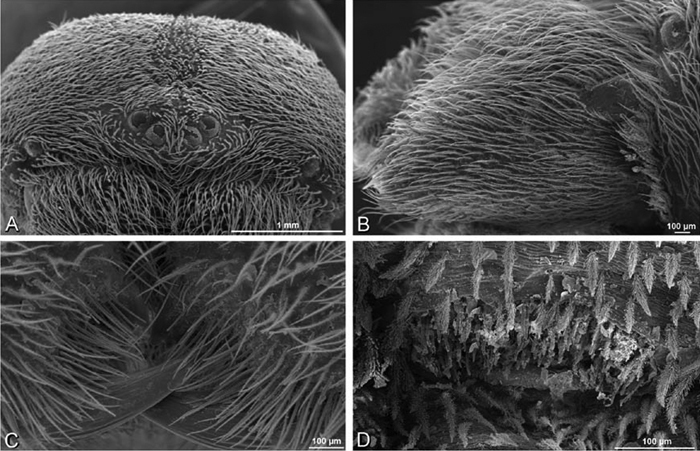
**A–D** *Dorceus fastuosus*, male from Mashabin sand dunes, Israel (MR006, HUJ), scanning electron micrographs. **A** prosoma, anterior view **B** left chelicerae, lateral view **C** chelicerae, anterior distal view showing fangs and teeth **D** epiandrous region.

*Male* (Mashabin Sand Dunes, Israel, MR006, HUJ): Carapace with few white setae; cephalic region subrectangular, wider than long, strongly raised, with silvery patches around some eyes; AME slightly smaller than PME (AME/PME 0.95), median eyes adjacent on horizontal axis, slightly overlapping on vertical axis; ALE tubercles absent; PER as wide as AER (PER/AER 0.99), PLE position on carapace 0.45; clypeal hood forms a nearly 90° angle; fovea moderately deep (Figs 8E, F, 26A–D, 28A). Chelicerae slightly excavated mesally, with lateral boss (Figs 26C, 28B). Legs with bands of white setae; with row of distal ventral macrosetae on metatarsus I–IV, one subdistal ventral macroseta on tibia IV, and scattered ventral macrosetae on metatarsus and tarsus I–IV, strongest and most numerous on metatarsus and tarsus IV. Abdomen gray, white dorsally with large dark heart mark (Fig. 26A).

Male palp with proximal-distal axis; tegulum bulbous; conductor and embolus together form apical complex making one helical turn (Fig. 27C, E); conductor tapers to point; tegular division longer than embolic division; cymbium with several prolateral macrosetae (Figs 12D–F, 26I, J, 27A–E).

**Figure 29. F29:**
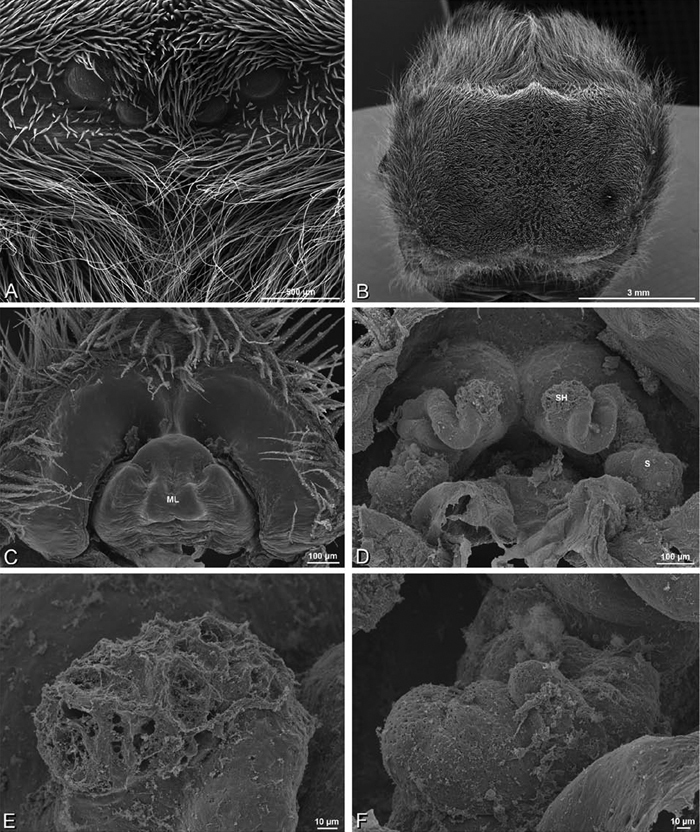
**A–F** *Dorceus fastuosus*, female from Mashabim sand dunes, Israel (MR002, MR), scanning electron micrographs. **A** median eye group **B** prosoma, dorsal **C** epigynum, ventral view **D** cleared vulva, dorsal view **E** detail, left spermathecal head **F** detail, right spermatheca. **ML** median lobe **S** spermatheca **SH** spermathecal head.

*Female* (Mashabim Reserve, Israel, MR): Carapace with scattered white setae; cephalic region subrectangular, wider than long, strongly raised (Fig. 26H); AME slightly smaller than PME (AME/PME 0.83), median eyes slightly overlapping on horizontal axis, separated on vertical axis; ALE tubercles absent; PER as wide as AER (PER/AER 0.97), PLE position on carapace 0.47; clypeal hood forms a nearly 90° angle; fovea moderately deep (Figs 8G, H, 26E–H, 29A, B). Chelicerae contiguous mesally, with lateral boss (Fig. 26G). Legs with row of distal ventral macrosetae on metatarsus III–IV and numerous macrosetae on metatarsus and tarsus III–IV. Abdomen without conspicuous white setae (Fig. 26E, H).

Epigynum with curved, converging slit-like atria occupying ca. the posterior half, anterior-lateral margin a curved ridge with median septum leading to subtrapezoidal median lobe (Figs 16B, 29C). Vulva with spermathecal heads set anterior-mesally on sinuous stalks leading to multilobed spermathecae that diverge posteriorly (Figs 16E, 29D–F).

**Figure 30. F30:**
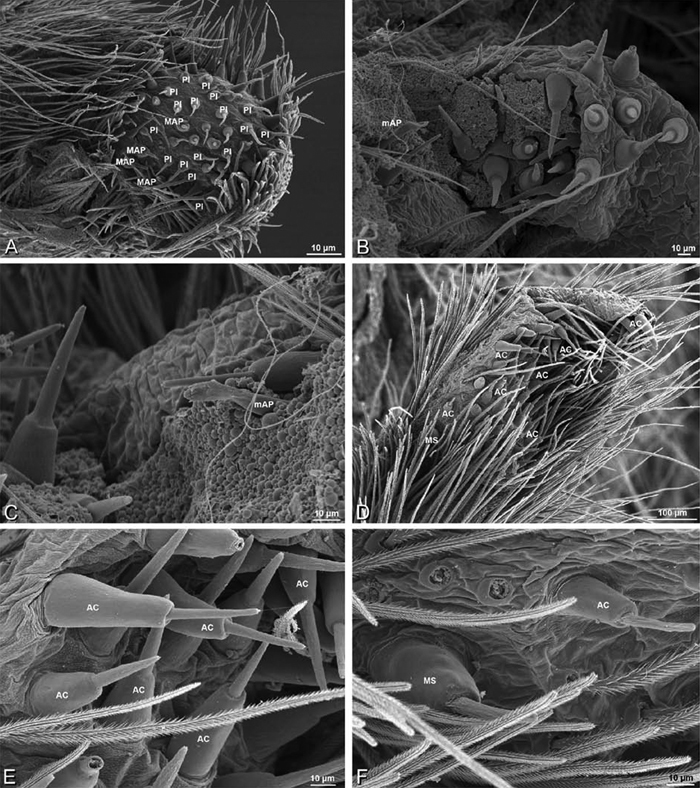
**A–F** *Dorceus fastuosus*, female from Mashabim sand dunes, Israel (MR002, MR), scanning electron micrographs of spinnerets. **A** left ALS **B** left PMS **C** detail of left PMS **D** left PLS **E** aciniform field on left PLS **F** modified spigot and flanking aciniform gland spigot on left PLS. Unlabeled spigots in **B** and **C** thought to be a mixture of aciniform gland spigots and cylindrical gland spigots. **AC** aciniform gland spigot **MAP** major ampullate gland spigot **mAP** minor ampullate gland spigot **MS** modified spigot **PI** piriform gland spigot.

**Figure 31. F31:**
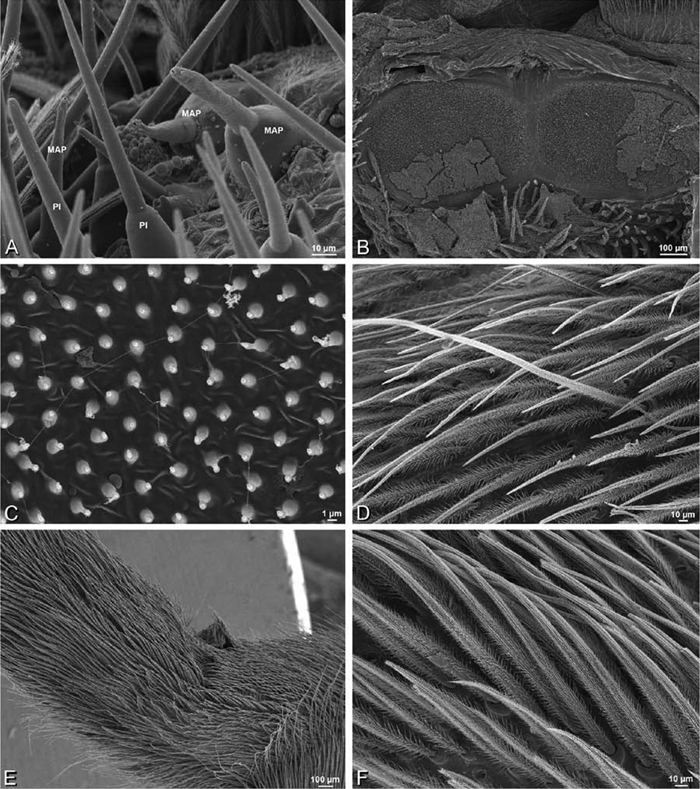
**A–F** *Dorceus fastuosus*, female from Mashabim sand dunes, Israel (MR002, MR), scanning electron micrographs. **A** detail of spigots on left ALS **B** cribellum **C** detail cribellar spigots **D** trichobothrium, left tibia IV **E** calamistrum, left metatarsus IV **F** detail, calamistrum seta, left metatarsus IV. **MAP** major ampullate gland spigot **PI** piriform gland spigot.

**Figure 32.**
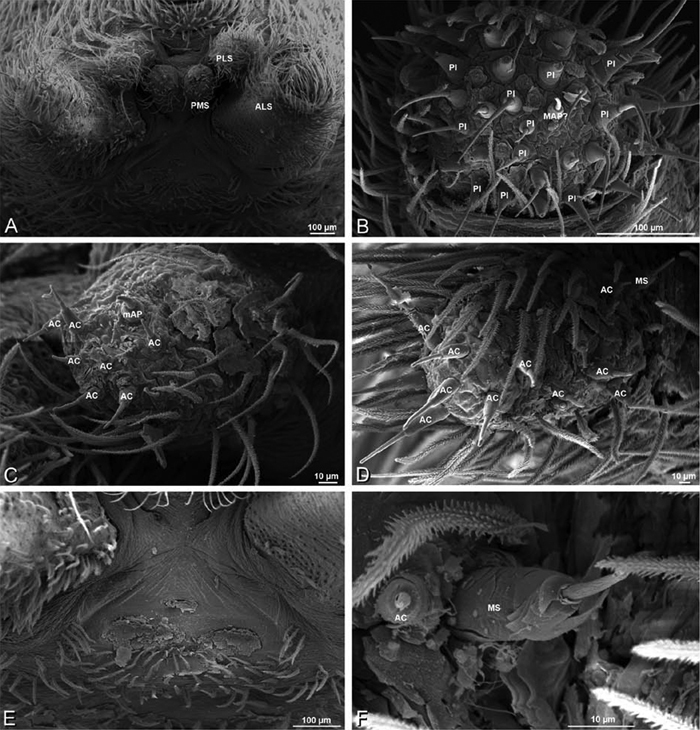
**A–F** *Dorceus fastuosus*, male from Mashabin Sand Dunes, Israel (MR006, HUJ), scanning electron micrographs of spinnerets. **A** overview **B** left ALS **C** left PMS **D** left PLS **E** vestigial cribellum **F** modified spigot and flanking aciniform spigot on left PLS. **AC** aciniform gland spigot **ALS** anterior lateral spinneret **MAP** major ampullate gland spigot **mAP** minor ampullate gland spigot **MS** modified spigot **PI** piriform gland spigot **PLS** posterior lateral spinneret **PMS** posterior median spinneret.

#### Spinneret spigot morphology

(Mashabim sand dunes, Israel, MR002, MR and MR006, HUJ)**.** Female ALS with at least 3 MAP near inner edge of spinning field of more the 50 PI (Figs 30A, 31A); male with about 25 PI spigots, 1 possible MAP visible (Fig. 32B). Female PMS with 1 anterior mAP and 20 spigots of various sizes posterior to this (Fig. 30B, C); male with 1 mAP and only 7 AC spigots (Fig. 32C), suggesting that female may have AC and CY spigots. Female PLS with anterior-basal MS and 1 accompanying AC, distal field of about 40 AC (Fig. 30D–F); male same except with only 12 AC (Fig. 32D). Male cribellar plate with no sign of spigots (Fig. 32E); epiandrous gland spigots present (Fig. 28D).

### Dresserus

Simon

http://species-id.net/wiki/Dresserus

Dresserus Simon 1876: LXXXVII [87]. Type species *Dresserus fuscus* Simon, 1876.

#### Note. 

*Dresserus* contains 24 recognized species from eastern and southern Africa (Platnick 2011). We examined specimens of multiple unidentified *Dresserus* species. Observations of the male plus the female spinneret spigot morphology is based on specimens from Mazumbai, West Usambara Mts., Tanzania (CASENT 9025746, CAS and CASENT 9025747, CAS); other observations of the female based on specimens from Klein Kariba (CASENT 9025745, CAS) and Drummond (CASENT 9037024, CAS), South Africa; other observations of the male based on specimens from Manga Forest Reserve, Tanzania (Frontier Tanzania, ZMUC), Hluhluwe Game Reserve, South Africa (TM 19739, TMSA), and Witbank, South Africa (TM 19738, TMSA).

#### Diagnosis.

**Figure 33.**
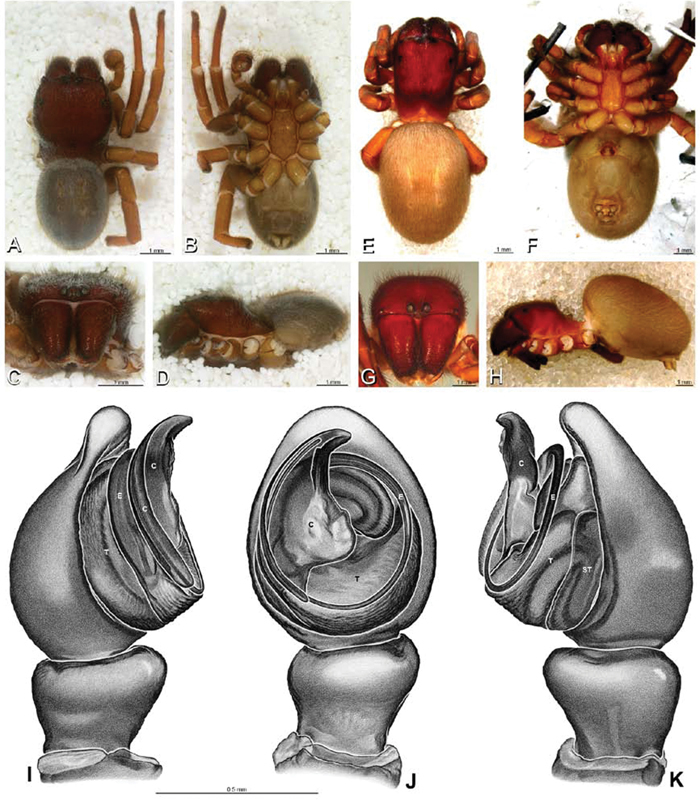
**A–K** *Dresserus* sp. **A–D** male from Manga Forest Reserve, Tanzania (ZMUC), image D reversed **E–H** female from Mazumbai, Tanzania (CASENT 9025747, CAS) **I–K** male from Mazumbai, Tanzania (CASENT 9025746, CAS) **A–D** habitus of male, photomicrographs **E–H** habitus of female, photomicrographs **I–K** illustrations of left male palp **A, E** dorsal view **B, F** ventral view **C, G** anterior view **D, H** lateral view **I** prolateral view **J** ventral view **K** retrolateral view. **C** conductor **E** embolus **ST** subtegulum **T** tegulum.

**Figure 34. F34:**
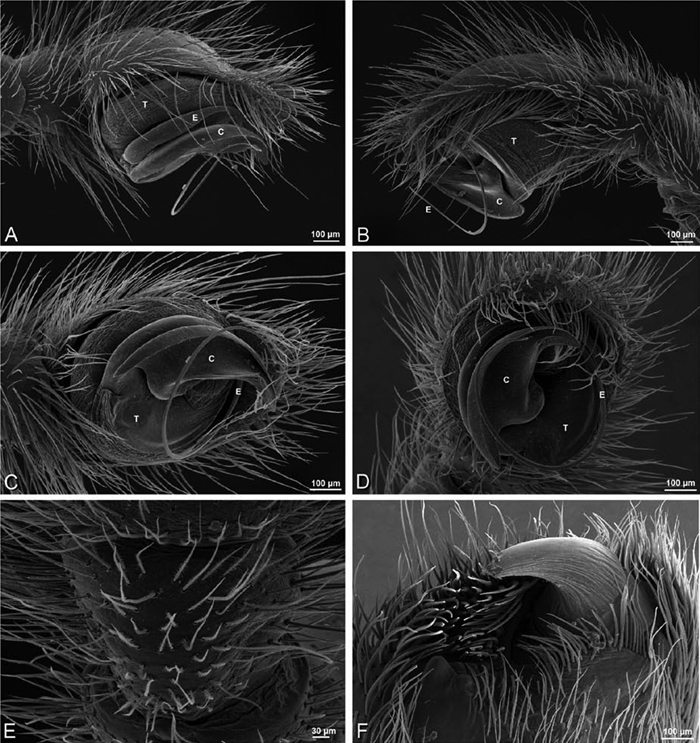
**A–F** *Dresserus* sp. **A–E** male from Mazumbai, Tanzania (CASENT 9025746, CAS), scanning electron micrographs of right palp, images reversed to appear as left palp **F** female from Klein Kariba, South Africa (CASENT 9025745, CAS), scanning electron micrographs of left chelicera **A** prolateral view **B** retrolateral view **C** ventral view **D** apical view **E** palpal tibia, dorsal view **F** distal part of chelicerae showing fang and teeth. **C** conductor **E** embolus **T** tegulum.

Distinguished from other eresids except *Gandanameno* by the position of the PLE which are both advanced (< 0.28) and widely spaced (PER/AER > 0.95; Fig. 8J); other eresids with advanced PLE (e.g., *Stegodyphus*, some *Paradonea*) have them closer together (PER/AER < 0.90) than the ALE (Figs 10B, 11B, D, F, H, J, L). Generally distinguished from other eresids by the distinctive 4-part cribellum (Fig. 36F), although some *Gandanameno* specimens show signs of this characteristic. Males distinguished from other eresids by the prominent tubercles bearing the ALE (Figs 8J, 33A), which are much more pronounced from those in other genera with ALE tubercles (e.g., *Stegodyphus*, some *Paradonea*). Further distinguished from other eresids except *Gandanameno* by the more or less ventral-dorsal axis of the palpal bulb with the embolus encircling the ventral part (Figs 12G–I, 33I–K, 34A–D; proximal-ventral in other eresids with the embolus encircling the distal part); distinguished from *Gandanameno* by the smooth conductor (Fig. 34C, D; fringed in *Gandanameno*, Fig. 55C, E). The cephalic region may be wider than long (e.g., TM 19738, TMSA, Frontier Tanzania, ZMUC) or longer than wide (e.g., TM 19739, TMSA). Female further distinguished from other eresids except *Gandanameno* and *Eresus walckenaeri* by the copulatory openings, which are broadly separated by hirsute cuticle (Figs 16C, 37D; separated by a glabrous median lobe in other eresids, e.g., Figs 16B, 29C); *Gandanameno* and *Dresserus* together distinguished from other eresids including *Eresus walckenaeri* by the vulva, which have the spermatheca extending anterior of the spermathecal heads, together subtended by helical copulatory ducts (Figs 16F, 37E; other eresids have the spermathecal head anterior, spermatheca posterior, and copulatory ducts other than helical), and by the subdivided PMS (entire in other eresids) with numerous short, conical, cylindrical(?) gland spigots (Fig. 36C, D; this spigot morphology absent in other eresids); distinguished from *Gandanameno* by the somewhat less prominent paired atria and possibly by having a single loop of the copulatory duct (Fig. 37E; three in *Gandanameno*, although this character has been investigated in few *Dresserus* species).

#### Distinguishing species.

Thetaxonomic literature on *Dresserus* is fragmentary and rarely comparative. This genus is ripe for revision. We have been unable to confidently assign species names to the specimens used in this study. Lehtinen (1967: 231) indicated that he was working on a taxonomic revision, but none was ever published. According to him, all but one of the described species (including the type species) had been checked and verified as congeneric based on primary types and other material.

#### Natural history.

Known from Savanna, stony semidesert, and forest habitats. They build a silken tube under stones. Prey is mainly beetles (Milan Řezáč, personal observation). Clutches consist of tens of juveniles (Martin Forman, personal observation of *Dresserus kannemeyeri*). Juveniles do not feed on their mother’s corpse. Mature females can live for several years in captivity and can produce a number of sequential clutches (Martin Forman, personal observation of *Dresserus kannemeyeri*).

### Dresserus sp.

Figs 1D 4B 8I–L 12G–I 16C, F 33 [Fig F34] [Fig F35] [Fig F36] [Fig F37] [Fig F38] –39

#### Description.

*Male* (Manga Forest Reserve, Tanzania, ZMUC): Carapace with few white setae, mostly in thoracic region; cephalic region subrectangular, wider than long, moderately raised; AME distinctly smaller than PME (AME/PME 0.71), median eyes overlapping on horizontal axis, separated on vertical axis; ALE placed on pointed apophyses, PER slightly wider than AER (PER/AER 1.04), PLE position on carapace 0.27, clypeal hood forms obtuse angle, fovea deep. Chelicerae slightly excavated mesally, with lateral boss. Legs with white setae, with row of distal ventral macrosetae on metatarsus II–IV. Abdomen dark gray, nearly encircled by a band of white setae (Figs 8I, J, 33A–D).

Male palp with dorsal-ventral axis; tegulum disc-shaped; conductor arises on broad membranous stalk from center of tegulum, with arching distal and proximal arms covering much of mesal portion of palpal bulb, distal arm larger than proximal, fringe absent (Fig. 34D); embolus makes slightly more than one loop, long and flexible, fits into groove originating on prolateral arm of conductor; cymbium without distinct macrosetae (Figs 12G–I, 33I–K, 34A–D).

**Figure 35. F35:**
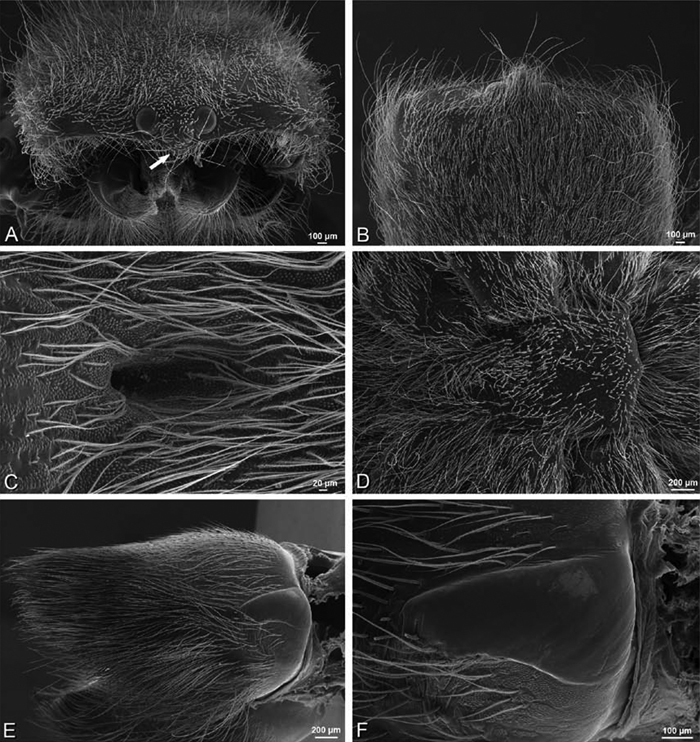
**A–F** *Dresserus* sp., female from Klein Kariba, South Africa (CASENT 9025745, CAS), scanning electron micrographs of prosoma. **A** anterior view, chelicerae removed, arrow indicates clypeal hood **B** dorsal view of eye region **C** fovea **D** sternum **E** right chelicera, ectal view **F** right cheliceral boss.

*Female* (Mazumbai, Tanzania, CASENT 9025747, CAS): Carapace without conspicuous white setae; cephalic region subrectangular, about as wide as long, moderately raised; AME distinctly smaller than PME (AME/PME 0.41), median eyes overlapping on horizontal axis, separated on vertical axis; ALE tubercles absent; PER as wide as AER (PER/AER 1.04), PLE position on carapace 0.27; clypeal hood forms obtuse angle, fovea deep. Chelicerae contiguous mesally, with lateral boss (Fig. 35E, F). Legs with white setae, with row of distal ventral macrosetae on metatarsus II–IV. Abdomen without conspicuous white setae (Figs 8K, L, 33E–H).

Epigynum with pair of longer-than-wide atria on posterior margin separated by hirsute cuticle (Figs 16C, 37D). Vulva with copulatory ducts making one loop leading to anterior complex of spermatheca and spermathecal head. Fertilization duct runs posteriorly through the copulatory duct loop (Figs 16F, 37E).

**Figure 36. F36:**
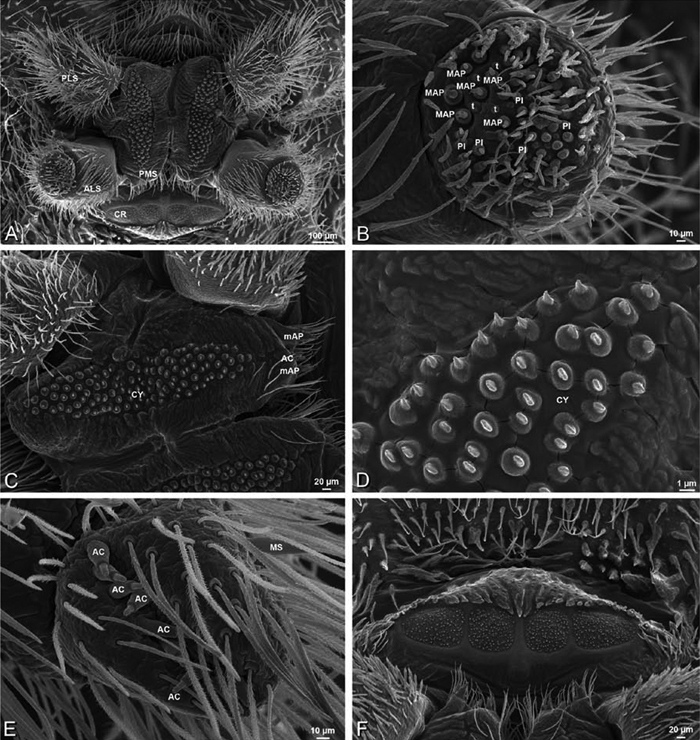
**A–F** *Dresserus* sp., female from Mazumbai, Tanzania (CASENT 9025747, CAS), scanning electron micrographs of spinnerets. **A** overview **B** left ALS **C** right PMS **D** detail, cylindrical gland spigots on right PMS **E** left PLS **F** cribellum. **AC** aciniform gland spigot **ALS** anterior lateral spinneret **CR** cribellum **CY** cylindrical gland spigot **MAP** major ampullate gland spigot **mAP** minor ampullate gland spigot **MS** modified spigot **PI** piriform gland spigot **PLS** posterior lateral spinneret **PMS** posterior median spinneret.

**Figure 37. F37:**
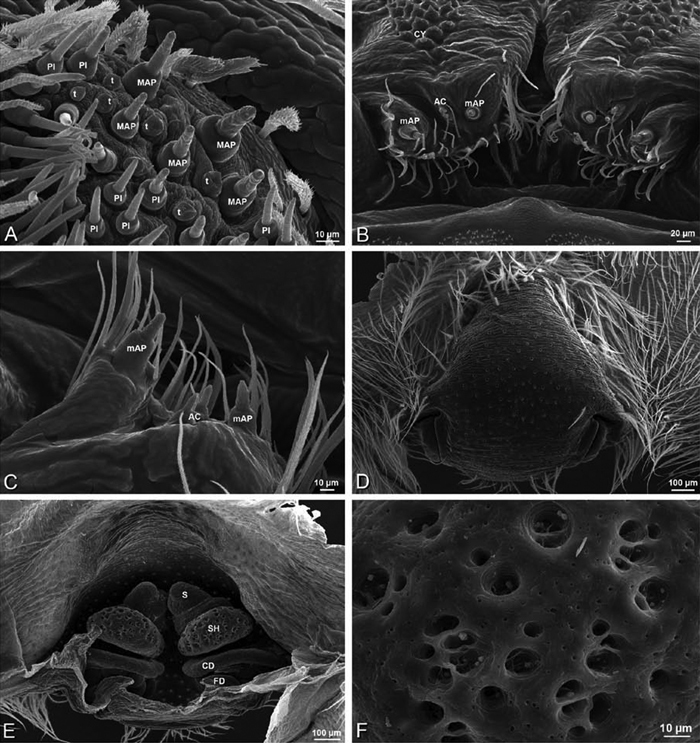
**A–F** *Dresserus* sp., scanning electron micrographs. **A, C** female from Mazumbai, Tanzania (CASENT 9025747, CAS) **D, F** female from Klein Kariba, South Africa (CASENT 9025745, CAS) **A** detail of spigots on right ALS **B** detail of spigots on anterior part of PMS **C** detail of spigots on anterior part of right PMS **D** epigynum, ventral view **E** vulva, dorsal view **F** detail of pores on right spermathecal head. **AC** aciniform gland spigot **CD** copulatory duct **CY** cylindrical gland spigot **FD** fertilization duct **MAP** major ampullate gland spigot **mAP** minor ampullate gland spigot **PI** piriform gland spigot **S** spermatheca **SH** spermathecal head **t** tartipore.

**Figure 38. F38:**
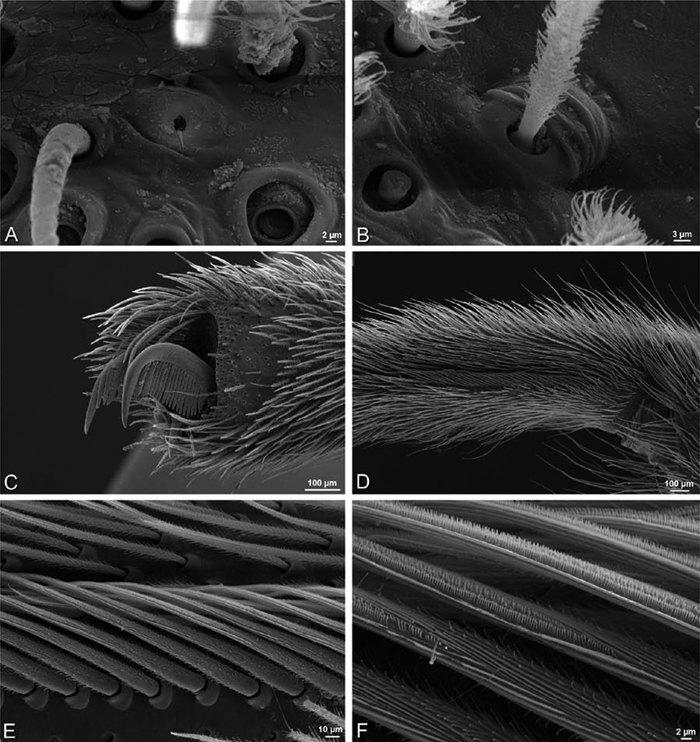
**A–F** *Dresserus* sp., female from Klein Kariba, South Africa (CASENT 9025745, CAS), scanning electron micrographs of legs **A** tarsal organ, left leg I **B** trichobothrium, left leg I **C** tarsal claw, left leg I setae removed **D** left metatarsus IV, retrolateral view, showing calamistrum **E** detail of calimistrum **F** detail of teeth on calimistrum setae.

**Figure 39.**
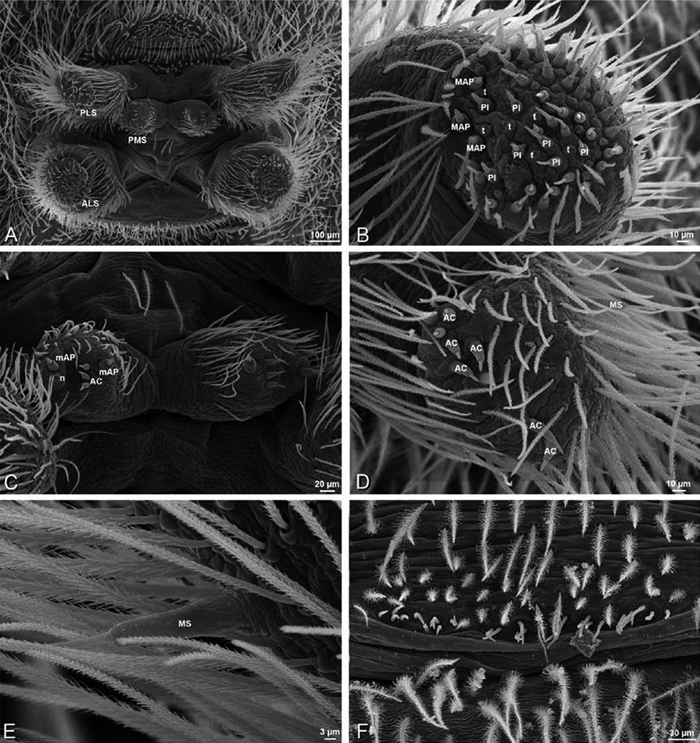
**A–F** *Dresserus* sp., male from Mazumbai, Tanzania (CASENT 9025747, CAS), scanning electron micrographs of spinnerets and epiandrous region. **A** overview of spinnerets **B** right ALS **C** PMS **D** left PLS **E** modified spigot on right PLS **F** epiandrous region. **AC** aciniform gland spigot **ALS** anterior lateral spinneret **MAP** major ampullate gland spigot **mAP** minor ampullate gland spigot **MS** modified spigot **n** nubbin **PI** piriform gland spigot **PLS** posterior lateral spinneret **PMS** posterior median spinneret **t** tartipore.

#### Spinneret spigot morphology

(Mazumbai, Tanzania, CASENT 9025746, CAS and CASENT 9025747, CAS)**.** Female ALS with at least 4 MAP within and 4–6 along inner edge of spinning field of more than 40 PI (Figs 36B, 37A); male with 4 MAP within and 3–5 along inner edge of spinning field of about 35 PI spigots (Fig. 39B). Female PMS longitudinally elongate, transversely bilobed, with 2 anterior mAP, between these 2–3 AC, posterior to this on anterior and posterior lobes a dense field of more than 105 short, squat, conical CY spigots (Figs 36C, D, 37B, C); male PMS small, oval, with 2 anterior mAP and 3 AC (Fig. 39C). Female PLS with anterobasal MS without accompanying spigot and distal field of 9 AC (Fig. 36E); male same (Fig. 39D, E). Male cribellar plate with no sign of spigots; epiandrous gland spigots present (Fig. 39F).

### Eresus

Walckenaer

http://species-id.net/wiki/Eresus

Eresus Walckenaer, 1805: 21. Type species *Aranea cinnaberina* Olivier, 1789.

#### Note. 

*Eresus* contains 23 recognized species group names (including 6 subspecies) from the Mediterranean and temperate latitudes of Europe and Asia. We examined specimens of several species representing the two major morphological groups within the genus. Our first exemplar, *Eresus walckenaeri*, is a cohesive species, based on both morphological and molecular data (Johannesen et al. 2005). Our specimens were from Bulgaria and Greece (Kresna, Bulgaria, MR; Pieria, Greece, MR020, MR; Lakonia, Greece, ZMUC 00012903, ZMUC). The primary types of *Eresus walckenaeri* are probably lost, but the original description and drawings, and our use in part of nearly topotypic material, allow us to identify this species with confidence. The second major group within the genus is a complex of closely related species including *Eresus kollari* (including the junior synonyms *Eresus cinnaberinus* and *Eresus niger*) and *Eresus sandaliatus* (Martini & Goeze, 1778); we refer to this assemblage collectively as the *Eresus sandaliatus* group. The somatic description was based on specimens of *Eresus kollari* from Czechia (Srbsko, MR016, MR; Prague, MR007, MR); scanning electron micrographs of the male palp are from a specimen of *Eresus kollari* from Hungary (Remete Mountain, CASENT 9037134, CAS); scanning electron micrographs and some photographs of the female genitalia are from a specimen of *Eresus sandaliatus* from SE of Silkeborg, Denmark (CASENT 9039243, CAS). The spinneret spigot morphology of *Eresus sandaliatus* group species was described and scanned in Griswold et al. (2005: 24–27, figs 31–32, 33A–F, 34A–C). Řezáč et al. (2008) examined copious material from the *Eresus sandaliatus* group including one of the two syntypes of *Eresus kollari*.

We found no characters that clearly and simultaneously separate *Eresus* females from other eresid genera, despite support from molecular data for a monophyletic *Eresus* (Miller et al. 2010a). Separate diagnoses for females of the two species groups appear in the following section.

#### Diagnosis.

**Figure 40.**
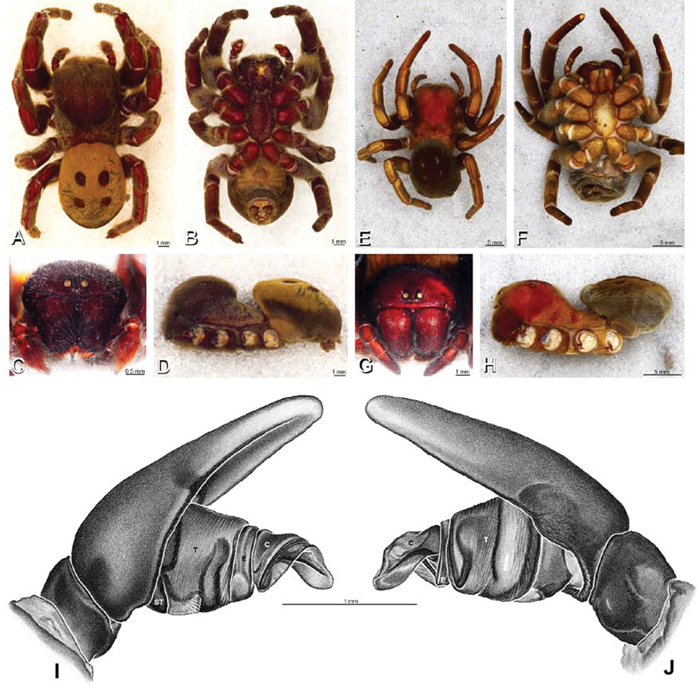
**A–J** *Eresus walckenaeri*. **A–D, I–J** male from Kresna, Bulgaria (MR) **E–H** female from 5 km south of Monemvasia, Lakonia, Greece (ZMUC 00012903, ZMUC) **A–D** habitus of male, photomicrographs **E–H** habitus of female, photomicrographs **I, J** illustrations of left male palp **A, E** dorsal view **B, F** ventral view **C, G** anterior view **D, H** lateral view **I** prolateral view **J** retrolateral view. **C** conductor **E** embolus **ST** subtegulum **T** tegulum.

Male *Eresus* are usually recognized by their distinctive dorsal abdominal pattern, which features two pairs of large round dark patches surrounding the first and second sigilla on a field of red setae, sometimes with a pair of smaller dark patches surrounding the third sigilla (Figs 2B, D, 40A, 43A); if with a black abdomen, then recognized by the distinctive notch on the embolic conductor (cf. Fig. 43J; note that black-abdomened form known as *Eresus tristis* Kroneberg, 1875 is currently cataloged as a synonym of *Eresus kollari*, but this is not accepted by all workers, e.g., Řezáč et al. 2008: 264, who noted that it is an “obviously different species”). Females of *Eresus walckenaeri* distinguished from other eresids except *Dresserus* and *Gandanameno* by the copulatory openings, which are broadly separated by hirsute cuticle (Figs 16G, 42B; separated by a glabrous median lobe in other eresids, e.g., Figs 16B, I, 29C, 45A), distinguished from *Dresserus* and *Gandanameno* by the more posterior position of the PLE (<0.28 in *Gandanameno* and *Dresserus*, > 0.33 in *Eresus walckenaeri*); females of the *Eresus sandaliatus* group distinguished from other eresids except *Paradonea variegata* by the large, bulbous spermathecal head (Figs 16K, L, 45B–D), distinguished from *Paradonea variegata* by the smaller size difference between the AME and PME (AME/PME > 0.5 in *Eresus sandaliatus* group, Fig. 9C; < 0.4 in *Paradonea variegata*, Fig. 10G) and by the overall darker color; thick stalks leading to the spermathecal head in some *Stegodyphus* species can be mistaken for large, bulbous spermathecal heads (Fig. 18J) although they are in fact compact sinuous ducts (Fig. 82B); *Eresus sandaliatus* group further distinguished from *Stegodyphus* by the more posterior position of the PLE (> 0.33 in *Eresus sandaliatus* group; < 0.29 in *Stegodyphus*).

#### Distinguishing species.

**Figure 41. F41:**
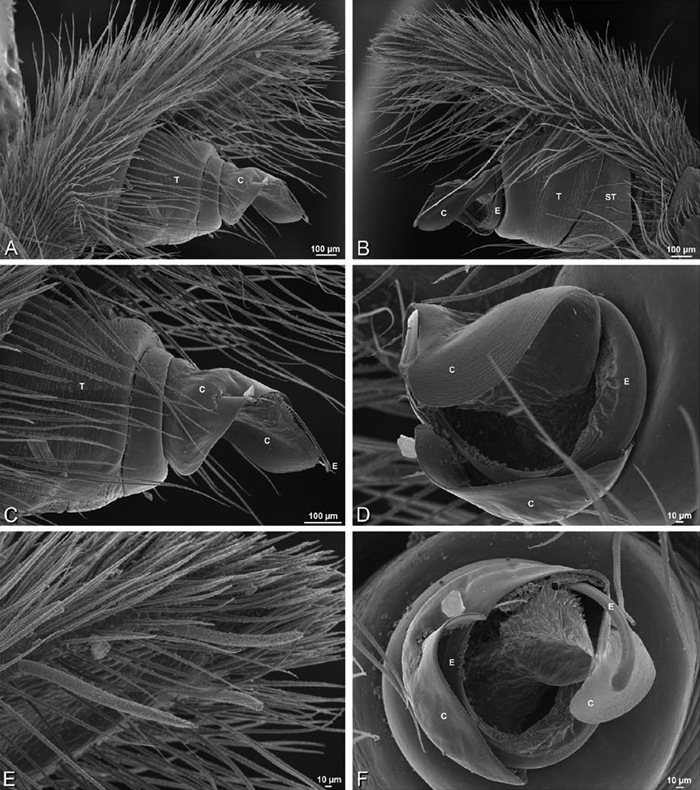
**A–F** *Eresus walckenaeri* from Kresna, Bulgaria (MR), scanning electron micrographs of right male palp, images reversed to appear as left palp. **A** prolateral view **B** retrolateral view **C** detail of embolic division, prolateral view **D** detail of embolic division, retrolateral view **E** apex of cymbium, ventral view **F** detail of embolic division, apical view. **C** conductor **E** embolus **ST** subtegulum **T** tegulum.

Male of *Eresus walckenaeri* distinguished from those of the *Eresus sandaliatus* group by the ribbon-like conductor with a blunt, rounded tip (Fig. 41C, D; notched in *Eresus sandaliatus* group, Fig. 44D, E) and the 1.5 helical turns of the embolus (ca. 1 helical turn in *Eresus sandaliatus* group). Various species within the *Eresus sandaliatus* group distinguished primarily by coloration and details of the conductor shape and vulva (Fig. 16H, I, K, L; see also Řezáč et al. 2008).

#### Natural history.

Known from various non-forest warm and dry habitats. Some species build a simple vertical burrow lined with silk. The opening is covered by silken sheet camouflaged from above by debris. Signaling threads radiate out from the edges of this roof. Some species (e.g., *Eresus walckenaeri*, *Eresus crassitibialis* Wunderlich) do not dig burrows; their silken tubes lie just under stones or on (Milan Řezáč, personal observation).

### Eresus walckenaeri

Brullé

http://species-id.net/wiki/Eresus_walckenaeri

Figs 2C–E 4C 12J–L 16G, J 40 [Fig F41] –42

Eresus walckenaeri Brullé, 1832: 54, pl. 28, fig. 4; Walckenaer 1837: 398; Simon 1884: 325–326; 1892: 248, fig. 201; Kulczyński 1903: 636, pl. 1, fig. 2; Giltay 1932: 12, fig. 5, 6; Brignoli 1978: 288, fig. 10; Johannesen et al. 2005: 1–8; Le Peru 2011: 321, fig. 561.
Eresus audouin Brullé, 1832: 54, pl. 28, fig. 10. (Synonymy in Simon 1884: 325).
Eresus theis Brullé, 1832: 54, pl. 28, fig. 11. (Synonymy in Simon 1884: 325).
Eresus ctenizoides C. L. Koch, 1836: 19, fig. 176. (Synonymy in Simon 1884: 325).
Eresus luridus C. L. Koch, 1836: 20, fig. 177. (Synonymy in Simon 1884: 325).
Eresus puniceus C. L. Koch, 1837: 102, fig. 315; Simon 1873: 345. (Synonymy in Simon 1884: 325).
Eresus pruinosus C. L. Koch, 1846: 3, fig. 1079. (Synonymy in Simon 1884: 325).
Eresus siculus Lucas, 1864: 28. (Synonymy in Simon 1884: 326).
Erythrophora punicea (C. L. Koch, 1837). Simon 1864: 300.

#### Description.

*Male* (Prague, Czechia, MR007, MR): Carapace with scattered white setae; cephalic region subtriangular, longer than wide, moderately raised; AME distinctly smaller than PME (AME/PME 0.63), median eyes adjacent on horizontal axis, slightly overlapping on vertical axis; ALE tubercles absent; PER slightly narrower than AER (PER/AER 0.89), PLE position on carapace 0.33; clypeal hood forms acute angle; fovea shallow. Chelicerae contiguous mesally, with lateral boss. Legs with clusters of white setae; with single distal ventral macroseta on metatarsus I, row of distal ventral macrosetae on metatarsus II–IV, scattered ventral macrosetae on metatarsus and tarsus II–IV, strongest and most numerous on metatarsus and tarsus IV, and retrolateral macroseta on metatarsus IV. Abdomen red dorsally with large dark patches surrounding anterior two pairs of sigilla (Figs 2D, 40A–D).

Male palp with proximal-distal axis; tegulum subtrapezoidal; conductor and embolus together form apical complex making 1.5 helical turns; conductor ribbon-like with blunt, rounded tip; tegular division longer than embolic division (Figs 12J–L, 40I, J, 41A–D, F); cymbium with several prolateral macrosetae (Fig. 41E).

*Female* (Srbsko, Czechia, MR016, MR): Carapace with few scattered white setae; cephalic region subtriangular, longer than wide, moderately raised; AME distinctly smaller than PME (AME/PME 0.63), median eyes separated on horizontal axis, adjacent on vertical axis; ALE tubercles absent; PER slightly narrower than AER (PER/AER 0.87), PLE position on carapace 0.34; clypeal hood forms acute angle; fovea shallow. Chelicerae contiguous mesally, with lateral boss. Legs without conspicuous white setae; legs with row of distal ventral macrosetae on metatarsus I–IV plus additional ventral macrosetae on metatarsus and tarsus I–IV. Abdomen without conspicuous white setae (Fig. 40E–H; see Fig. 2C for color of live animal).

**Figure 42.**
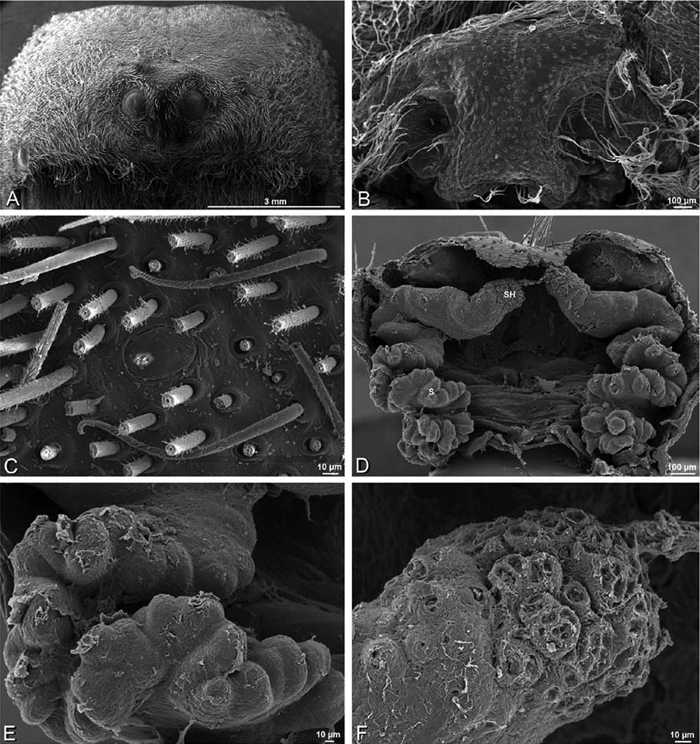
**A–F** *Eresus walckenaeri* female from 5 km south of Monemvasia, Lakonia, Greece (ZMUC 00012903), scanning electron micrographs. **A** prosoma, anterior view **B** epigynum **C** tarsal organ, left leg I. ventral view **D** vulva, dorsal view **E** left spermatheca **F** left spermathecal head. **S** spermatheca **SH** spermathecal head.

Epigynum with pair of wide atria on posterior margin separated by hirsute cuticle (Figs 16G, 42B). Vulva with spermathecal heads on long sinuous stalks gradually transitioning to sinuous multilobed spermathecae (Figs 16J, 42D–F).

### *Eresus sandaliatus* group

#### Eresus kollari

Rossi

http://species-id.net/wiki/Eresus_kollari

Figs 2A, B 9A–D 13A–C 16H, K 43 44 45E, F 46

Eresus kollari Rossi 1846: 17.. *See Platnick 2011 for additional synonymy*.

##### Description.

**Figure 43.**
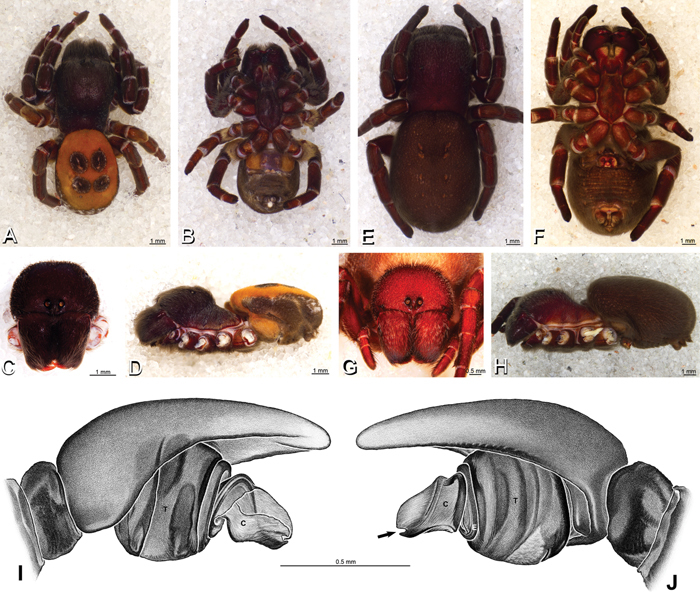
**A–J** *Eresus kollari*. **A–D,** **I, J** male from Prague, Czechia (MR007, MR) **E–H** female from res. Srbsko, Czechia (MR016, MR) **A–D** habitus of male, photomicrographs **E–H** habitus of female, photomicrographs **I, J** illustrations of left male palp **A, E** dorsal view **B, F** ventral view **C, G** anterior view **D, H** lateral view **I** prolateral view **J** retrolateral view, arrow indicates notch in conductor. **C** conductor **E** embolus **T** tegulum.

*Male* (Prague, Czechia, MR007, MR): Carapace with scattered white setae; cephalic region subrectangular with broadly rounded posterior margin, longer than wide, moderately raised; AME distinctly smaller than PME (AME/PME 0.63), median eyes slightly separated on horizontal axis, slightly overlapping on vertical axis; ALE tubercles absent, PER slightly narrower than AER (PER/AER 0.89), PLE position on carapace 0.39; clypeal hood forms acute angle; fovea moderately deep. Chelicerae contiguous mesally, with lateral boss. Legs with bands of white setae; with row of distal ventral macrosetae on metatarsus I–IV plus additional ventral macrosetae on tibia, metatarsus and tarsus I–IV. Abdomen red dorsally with large dark patches surrounding anterior two pairs of sigilla (Figs 2B, 9A, B, 43A–D).

**Figure 44.**
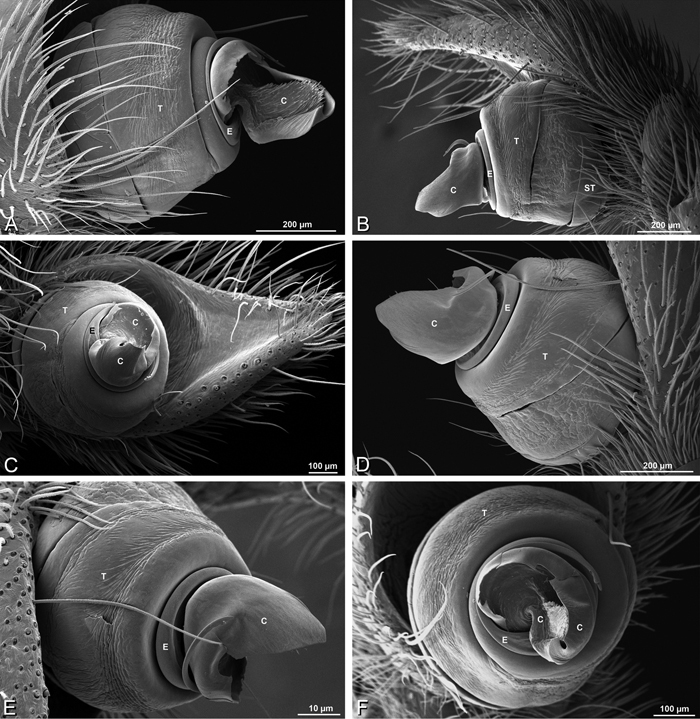
**A–F** *Eresus kollari* from Remete Mountain, Hungary (CASENT 9037134, CAS), scanning electron micrographs of left male palp. **A** prolateral view **B** retrolateral view **C** ventral view **D** palpal bulb, retrolateral view **E** palpal bulb, retrodorsal view **F** apical view. **C** conductor **E** embolus **ST** subtegulum **T** tegulum.

Male palp with proximal-distal axis; tegulum bulbous; conductor and embolus together form apical complex making one helical turn; conductor ribbon-like, tip broadly rounded with a notch (Figs 13A–C, 43I, J, 44A–F; Řezáč et al. 2008: fig. 5A, D); tegular division longer than embolic division; cymbium with several prolateral macrosetae.

*Female* (Srbsko, Czechia, MR016, MR): Carapace with scattered white setae; cephalic region subrectangular with broadly rounded posterior margin, longer than wide, moderately raised; AME distinctly smaller than PME (AME/PME 0.63), median eyes slightly overlapping on horizontal and vertical axes; ALE tubercles absent; PER slightly narrower than AER (PER/AER 0.89), PLE position on carapace 0.39; clypeal hood forms acute angle; fovea shallow. Chelicerae contiguous mesally, with lateral boss. Legs with scattered white setae, with row of distal ventral macrosetae on metatarsus I–IV plus additional ventral macrosetae on metatarsus and tarsus III–IV. Abdomen without conspicuous white setae (Figs 2A, 9C, D, 43E–H).

**Figure 45.**
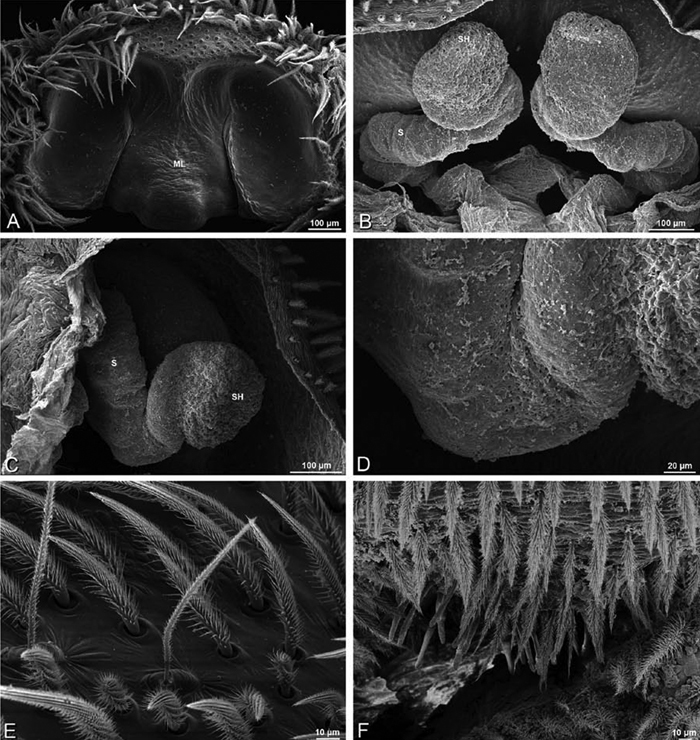
**A–F** *Eresus* spp., scanning electron micrographs. **A–D** *Eresus sandaliatus* female from SE of Silkeborg, Denmark (CASENT 9039243, CAS) **E** *Eresus kollari* female from Srbsko, Czechia (MR016, MR) **F** *Eresus kollari* male from Prague, Czechia (MR007, MR) **A** epigynum, ventral view **B** vulva, dorsal view **C** left spermathecal head **D** detail, left spermatheca **E** trichobothria, left tibia I **F** epiandrous region. **ML** median lobe **S** spermatheca **SH** spermathecal head.

Epigynum with slit-like atria occupying nearly the total length, anteriomedian part with notch-shaped invagination, anteriolateral margin a curved ridge (Figs 16H, 45A; Řezáč et al. 2008: fig. 4G). Vulva with large, bulbous spermathecal heads anteriorly leading to posterior spermathecae with multiple weakly-differentiated lobes (Fig. 16K; cf. Figs 16L, 45B–D; Řezáč et al. 2008: fig. 4J).

**Figure 46.**
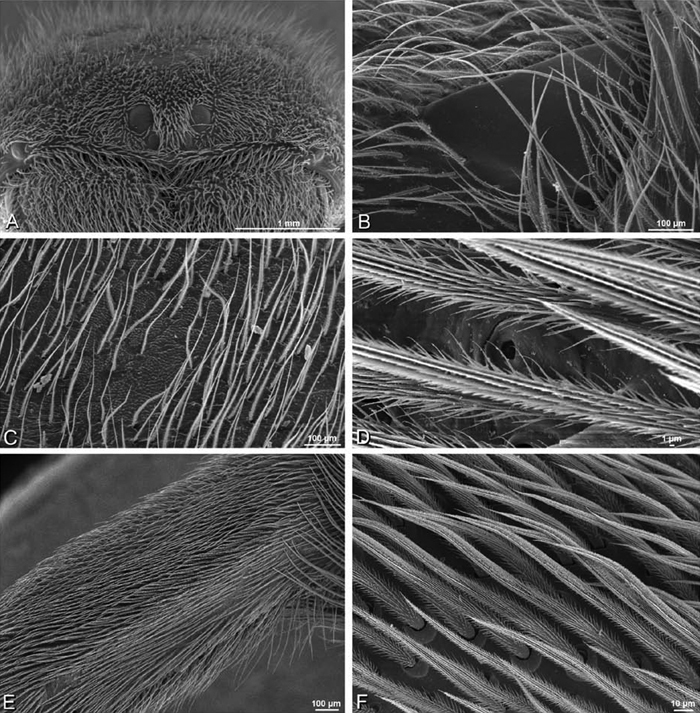
**A–F** *Eresus kollari* from Srbsko, Czechia (MR016, MR), scanning electron micrographs of female. **A** prosoma, anterior view **B** left cheliceral boss **C** detail of carapace texture **D** tarsal organ, left leg I **E** calamistrum, left metatarsus IV **F** detail, calamistrum seta, left metatarsus IV.

### Additional observations from the *Eresus sandaliatus* group

**Spinneret spigot morphology** (based on *Eresus* cf. *cinnaberinus* from Greece and Morocco in Griswold et al., 2005: figs 31A–D, 32A–D, 33A–F): Female ALS with at least 13 MAP within and on inner edge of spinning field of more than 120 PI; male with at 5 MAP and spinning field of more than 40 PI. Female PMS with anterior mAP spigots, with posterior field of more 40 small spigots of varying size and shape; male PMS with 4 mAP and 6 AC, suggesting the female may have AC and CY spigots. Female PLS with anterobasal MS with 2 accompanying spigots and distal field of about 55 AC; male MS MS with 2 accompanying spigot nubbins, with 9 AC. Male cribellar plate with no sign of spigots; numerous epiandrous gland spigots present.

#### Gandanameno

Lehtinen

http://species-id.net/wiki/Gandanameno

Gandanameno Lehtinen, 1967: 235. Type species *Eresus spenceri* Pocock, 1900.

##### Note.

*Gandanameno* contains five recognized species from eastern and southern Africa. We examined the collection holdings of several museums and most primary type specimens. The oldest available name, *Eresus fumosus* C. L. Koch, 1837, apparently lacks any type specimen (Lehtinen 1967: 235). We evaluated morphological variation and analyzed DNA sequences from 24 individuals based on previously published and new data. Descriptions are based on a female specimen from Tanzania (ZMUC 19970530, ZMUC) and a male from Zimbabwe (AcAT 2005/123, NCA) supplemented by collections mostly from South Africa.

##### Diagnosis.

Distinguished from other eresids except *Dresserus* by the position of the PLE which are both advanced (< 0.28) and widely spaced (PER/AER > 0.95; Fig. 9F, H); other eresids with advanced PLE (e.g., *Stegodyphus*, some *Paradonea*) have them closer together (PER/AER < 0.90) than the ALE (e.g., Figs 10B, 11F). Male further distinguished from other eresids except *Dresserus* by the more or less ventral-dorsal axis of the palpal bulb with the embolus encircling the ventral part (Figs 13D, E, 48A–C; proximal-ventral in other eresids with the embolus encircling the distal part, e.g., Fig. 20A–F) and conductor arising from the center of the tegulum with opposing projections covering much of the palpal bulb (Fig. 55C); distinguished from *Dresserus* by the lack of prominent tubercles bearing the ALE (Fig. 9F; compare with Fig. 8J) and the fringed conductor (Fig. 55C, E; smooth in *Dresserus*, Fig. 34D). Female further distinguished from other eresids except *Dresserus* and *Eresus walckenaeri* by the copulatory openings, which are broadly separated by hirsute cuticle (Figs 17A–C, 59A; separated by a glabrous median lobe in other eresids, e.g., Figs 16B, 29C); *Gandanameno* and *Dresserus* together distinguished from other eresids including *Eresus walckenaeri* by the vulva, which have the spermatheca extending anterior of the spermathecal heads, together subtended by helical copulatory ducts (Figs 17D–F, 59C; other eresids have the spermathecal head anterior, spermatheca posterior, and copulatory ducts other than helical, e.g., Fig. 29D), and by the subdivided PMS (Fig. 57C; entire in other eresids) with numerous short, conical, cylindrical gland spigots (Fig. 58E; cylindrical gland spigots absent or difficult to distinguish from aciniform gland spigots in other eresids); distinguished from *Dresserus* by the somewhat more prominent paired atria (Figs 17A–C, 59A; compare with Figs 16C, 37D) and possibly by having three loops of the copulatory duct (Figs 17D–F, 59C; fewer in *Dresserus*, although this character has been investigated in few *Dresserus* species, Figs 16F, 37E). Both sexes usually distinguished from *Dresserus* by the two part cribellum (Figs 57E, 60E, compare with Fig. 36F), although signs of the distinctive 4-part cribellum are sometimes apparent in *Gandanameno*.

##### Distinguishing species.

*Gandanameno* are variable in several conspicuous characteristics. In the male, these include the presence or absence of a cheliceral boss (Fig. 56C–F), palp size, details of the conductor shape, and the curvature and position of the tegular sperm duct (Figs 48B, D–F, S2A–J). In the female, these include the height of the cephalic region, and the presence of cuspules of various weights on the carapace, sternum, and basal segments of legs I, II, and sometimes III (Figs 50, 52A–F, 53A–F, 54A–F), also details of the epigynum shape (Figs 17A–C, S2K, L, S3A–F, H–L). It has been noted previously that multiple forms may occur sympatrically and that intermediate combinations make species determination problematic (Tucker 1920). Indeed, in most regions where several specimens are known, a wide range of variation can be found (Fig. 50). We attempted to sequence DNA from several museum specimens. Unfortunately, most of our successes were from specimens at one end of the range of variation, i.e., specimens with a moderately to strongly raised cephalic region and abundant, heavy cuspules on the carapace, sternum, and anterior legs. The three contrasting female specimens (13-10: KwaZulu-Natal; 18-04: AcAT 2002/181; 14517) did not group together. Instead, relationships appear to be more strongly tied to geography than to morphology (Fig. 51). The genitalia of 23 out of 24 *Gandanameno* specimens included in the molecular phylogenetic analysis are photo-documented in the electronic supplementary materials (Figs S2A–L, S3A–L).

We looked for patterns of variation in adult female morphology. Adult female specimens are much more abundant in collections than males. We defined eight geographic regions based on two criteria: 1) concentration of specimens available to us and/or 2) type localities of *Gandanameno* species (Figs 49, 50). We assessed a series of characters: carapace length, carapace width, presence and strength of cuspules on the ventral surface of femur I and II, prosoma, and sternum for all specimens available from these regions. When cuspules are present on the prosoma, they always occur on the femurs, but the reverse is not always true. So the set of specimens with cuspules on the femurs is larger than and contains the set of specimens with cuspules on the prosoma. There appears to be a general trend towards greater spinulation and a higher carapace with increasing size (carapace length), but the spinulation data are fairly noisy (Fig. 50). No morphological pattern emerged to segregate specimens by region. Not all degrees of spinulation were observed in every geographic region.

Based on the combination of genetic, morphometric, and spinulation data, we see no evidence that the characters traditionally used to discriminate *Gandanameno* species are valid. We speculate that ontogenetic factors are responsible for the morphological variation in this genus (perhaps juvenile nutrition or post adult molting). However, we judge that the synonymy of all *Gandanameno* species is premature, particularly in light of the phylogeographic signal suggested by the molecular data and the limited availability of specimens from beyond the Republic of South Africa. We therefore identify our specimens simply as *Gandanameno* sp. We encourage additional investigations that might further elucidate the systematics of *Gandanameno*.

##### Natural history.

Associated with crevices in or under rocks or tree bark in savanna, parks, and gardens. They build a silken tube with a widened entrance; the tube of an adult female under bark can be up to 1 m long (Martin Forman, personal observation). Also found under tree bark well above the ground in dense aggregations (Nikolaj Scharff, Jeremy Miller, Iringa, Tanzania). Prey remnants are placed at the bottom of the tube. Clutches consist of tens of juveniles (Martin Forman, personal observation). Juveniles do not feed on their mother’s corpse and adult females can produce a number of sequential clutches. Males mature in less than one year, females take longer (Martin Forman, personal observation).

#### Gandanameno sp.

Figs 1E, F 4D–F 9E–H 13D–F 17 47 [Fig F48] [Fig F49] –50 52 [Fig F53] [Fig F54] [Fig F55] [Fig F56] [Fig F57] [Fig F58] [Fig F59] [Fig F60] –61 S2, S3 

##### Description.

**Figure 47.**
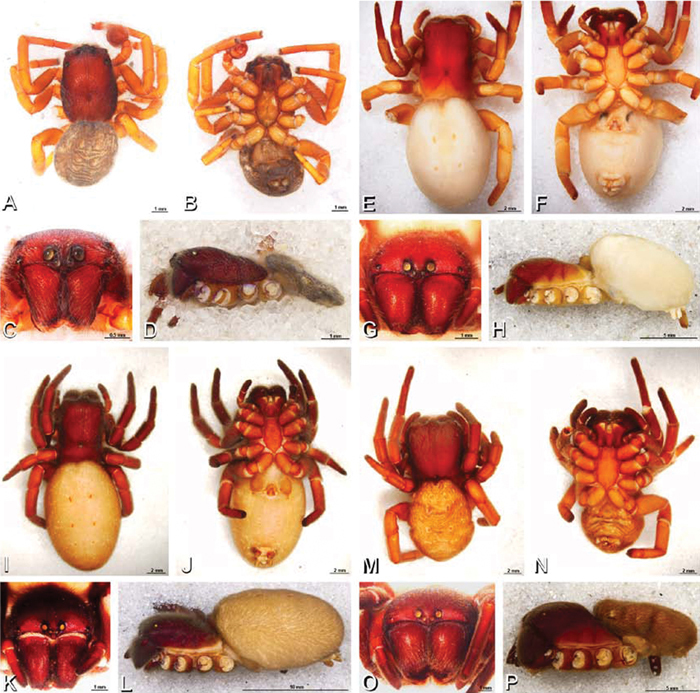
**A–P** *Gandanameno* sp., habitus, photomicrographs. **A–D** male from Harare, Zimbabwe (AcAT 2005/123, NCA), images reversed **E–H** female from Hanover, South Africa (SAM-ENW-B006896/9958, SAM) **I–L** female from Iringa, Tanzania (ZMUC 19970530, ZMUC) **M–P** female from Eierfontein, South Africa (SAM-12823, SAM) **A, E, I, M** dorsal view **B, F, J, N** ventral view **C, G, K, O** anterior view **D, H, L, P** lateral view.

*Male* (Harare, Zimbabwe, AcAT 2005/123, NCA): Male carapace with scattered white setae, cephalic region subrectangular, about as long as wide, slightly raised; AME distinctly smaller than PME (AME/PME 0.63), median eyes slightly overlapping on horizontal and vertical axes; ALE tubercles absent, PER nearly as wide as AER (PER/AER 0.95), PLE position on carapace 0.23; clypeal hood forms obtuse angle; fovea deep. Chelicerae contiguous mesally, without lateral boss (Fig. 56B–D; note that all other males examined have a cheliceral boss, Fig. 56E, F). Legs without conspicuous white setae; with row of distal ventral macrosetae on metatarsus I–IV. Abdomen dark without conspicuous white setae (Figs 9E, F, 47A–D).

**Figure 48. F48:**
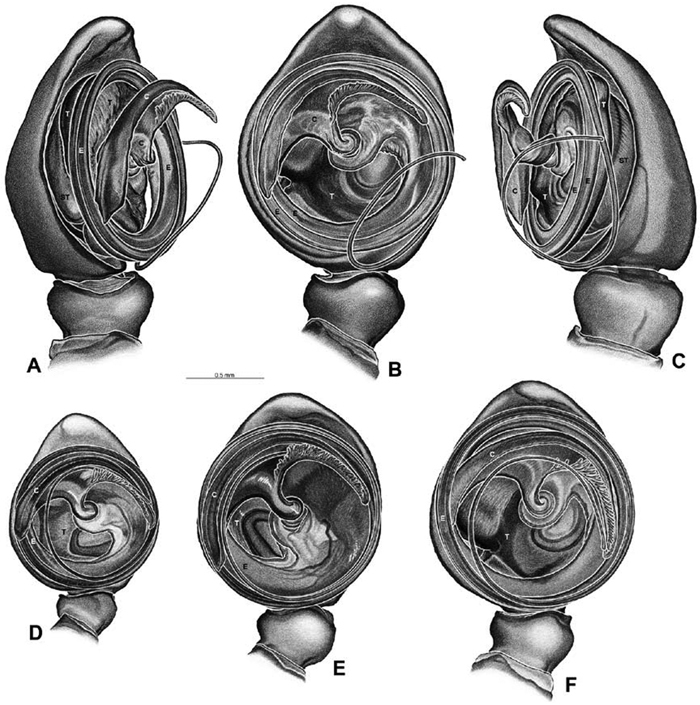
**A–F** *Gandanameno* sp., illustrations of left male palp. **A–C** from Naauwpoort, North West Province, South Africa (SAM 1600, SAM) **D** from Van Riebeeck Park, Western Cape, South Africa (CASENT 9023763, CAS) **E** from Graaff-Reinet, Eastern Cape, South Africa (SAM 12571, SAM) **F** from Hanover, South Africa (SAM 9465, SAM) **A** obliquely retrolateral view **B,** **D–F** ventral view **C** obliquely prolateral view. All images at the same scale. **C** conductor **E** embolus **ST** subtegulum **T** tegulum.

Male palp with dorsal-ventral axis; tegulum disc-shaped; conductor arises on stalk from center of tegulum, with arching prolateral and retrolateral arms covering much of anterior portion of palpal bulb, retrolateral arm with fringed posterior margin; embolus makes ca. three loops, long and flexible, fits into groove originating on prolateral arm of conductor; cymbium without distinct macrosetae (Figs 13D–F, 48A–F, 55A–E, S2A–J).

*Female* (Iringa, Tanzania, ZMUC 19970530, ZMUC): Female carapace without conspicuous white setae; cephalic region subrectangular, longer than wide, slightly raised, AME distinctly smaller than PME (AME/PME 0.58), median eyes slightly overlapping on horizontal axis, adjacent on vertical axis; ALE tubercles absent, PER as wide as AER (PER/AER 1.03), PLE position on carapace 0.20; clypeal hood forms obtuse angle; fovea deep. Chelicerae contiguous mesally, with lateral boss. Legs without conspicuous white setae; legs with row of distal ventral macrosetae on metatarsus III–IV. Abdomen without conspicuous white setae (Figs 4E, F, 9G, H, 47E–P).

Epigynum bell-shaped, with pair of semicircular atria on posterior margin separated by hirsute cuticle (Figs 17A–C, 59A, B, S2K, L, S3A–F, H–L). Vulva with copulatory ducts making three loops leading to anterior complex of spermatheca and spermathecal head. Fertilization duct runs posteriorly through the copulatory duct loops (Figs 17D–F, 59C–F, S3G).

##### Spinneret spigot morphology.

(Iringa, Tanzania, ZMUC 19970530, ZMUC; Hanover, South Africa, SAM-ENW-B006896/9958, SAM and SAM 9465, SAM): Female ALS with at least 7–8 MAP within and along inner edge of spinning field of 50–80 PI (Fig. 57B); male with 1 MAP within and 3 along inner edge of spinning field of about 40 PI spigots (Figs 60B, 61A). Female PMS longitudinally elongate, transversely bilobed, with 2–3 anterior mAP, between these 2–3 AC, posterior to this on anterior and posterior lobes a dense field of more than 40 (SAM-ENW-B006896/9958) to 55 (ZMUC 19970530, ZMUC) short, squat, conical CY spigots (Figs 57C, 58E, F); male PMS small, oval, with 2 mAP and 4 AC (Figs 60C, 61D). Female PLS with anterobasal MS without accompanying spigot and distal field of 9–15 AC (Figs 57D, 58C); male same (Figs 60D, 61B, C). Male cribellar plate with no sign of spigots (Fig. 60E, F); epiandrous gland spigots present (Fig. 61E, F). Cribellar plate divided medially, although some hint of subdivision into four fields of spigots (as in *Dresserus*) is evident (Fig. 57E).

**Figure 49. F49:**
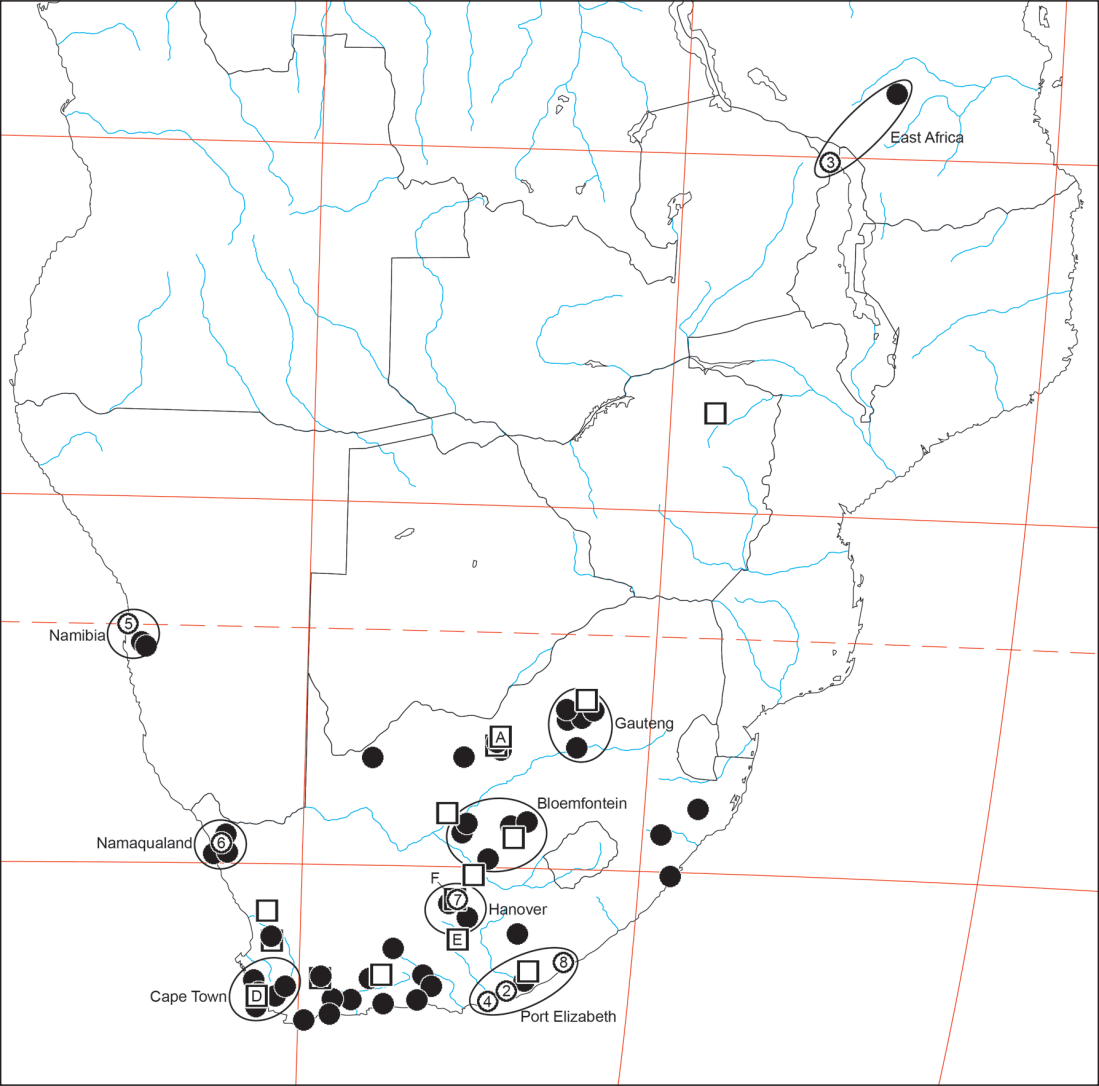
Distribution of *Gandanameno*. Type localities are numbered circles, males are squares (if with letters, these refer to illustrations in Fig. 48), non-type females are filled circles. Type localities: circle **2** *Eresus bubo* L. Koch, 1865; circle **3** *Eresus inornatus* Pocock, 1898; circle **4** *Eresus spenceri* Pocock, 1900; circle **5** *Eresus echinatus* Purcell, 1908; circle **6** *Eresus namaquensis* Purcell, 1908; circle **7** *Eresus depressus* Tucker, 1920; circle **8** *Eresus purcelli* Tucker, 1920; type locality of *Eresus fumosus* C. L. Koch, 1837 is reported simply as “Afrika" and no type specimen is known (Lehtinen 1967: 235). Localities of males illustrated in Fig. 48: square **A** Fig. 48A–C square **D** Fig. 48D square **E** Fig. 48E square **F** Fig. 48F. Ellipsoids indicate regions for size chart Fig. 50, region names are for convenience only.

**Figure 50.**
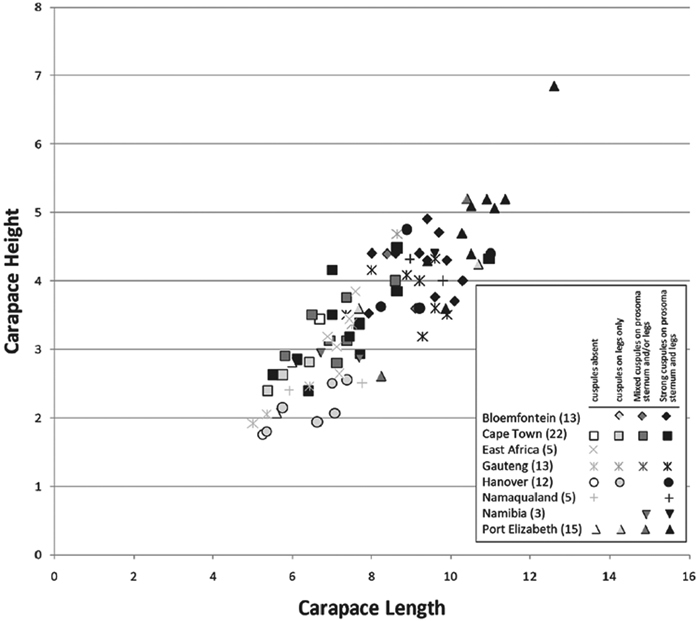
Carapace height plotted against carapace length for adult female *Gandanameno* specimens from eight regions: Bloemfontein, Cape Town, Gauteng, Hanover, Namaqualand, Namibia, and Port Elizabeth. Regions circumscribed in Fig. 49; sample size given in parentheses. Symbol shape indicates region while symbol darkness indicates presence and strength of cuspules. Specimens were scored as having cuspules absent, having medium to strong cuspules only on the legs, having a mixture of medium and strong cuspules on the prosoma, sternum, and/or legs, and having exclusively strong cuspules on the prosoma, sternum, and legs. As reflected in the legend, not all degrees of spinulation were observed in all regions.

**Figure 51.**
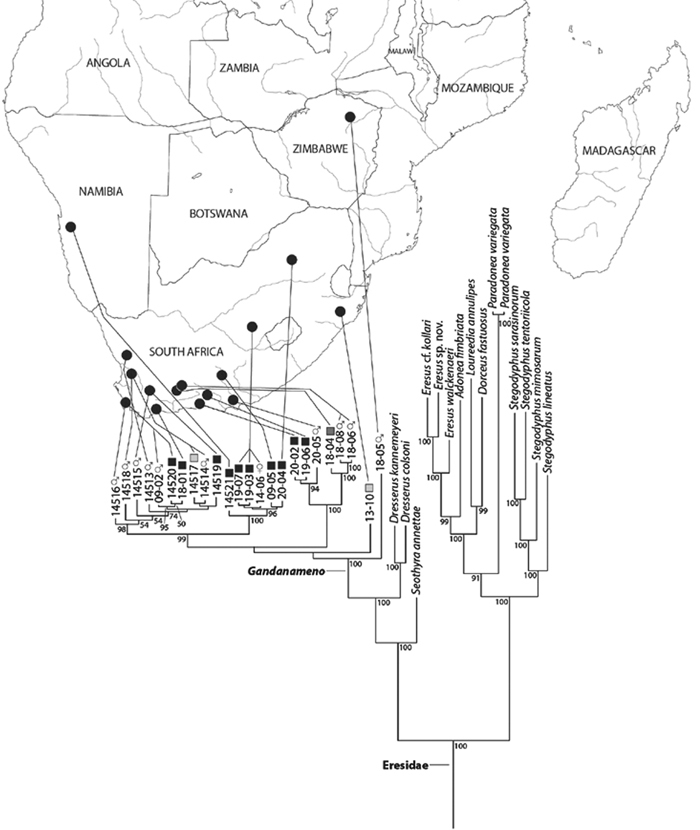
Bayesian phylogenetic tree of the spider family Eresidae based on mixed model analysis (eight data partitions, manually adjusted alignment; see Miller et al. 2010a); outgroups not shown, see Fig. S1. For the genus *Gandanameno*, DNA specimen codes are substituted for taxonomic name and specimens are linked to their collection locality in southern Africa. Male specimens indicated by male symbol, female specimens indicated either by a female symbol or a square, the darkness of which indicates the strength and presence of cuspules, scored as in Fig. 50. Branches drawn proportional to change. Numbers at nodes are percent posterior probabilities of 50 or greater.

**Figure 52.**
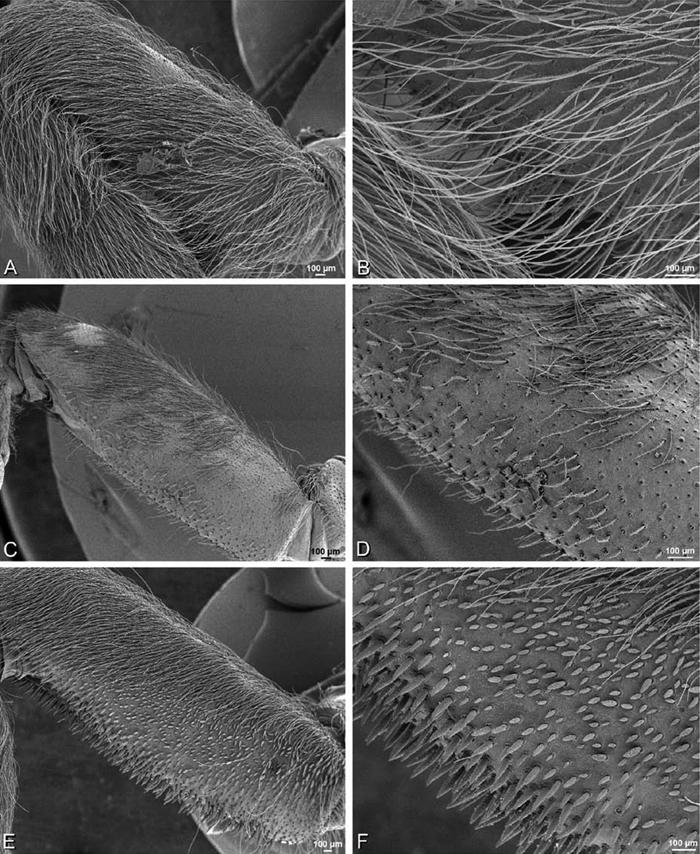
**A–F** *Gandanameno* sp., femur, left leg I of female, retrolateral view, scanning electron micrographs. **A, C, E** overview **B, D, F** detail of setae **A, B** from Iringa, Tanzania (ZMUC 19970530, ZMUC) **C, D** from Hanover, South Africa (SAM-ENW-B006896/9958, SAM) **E, F** from Eierfontein, Eastern Cape, South Africa (SAM-12823, SAM).

**Figure 53. F53:**
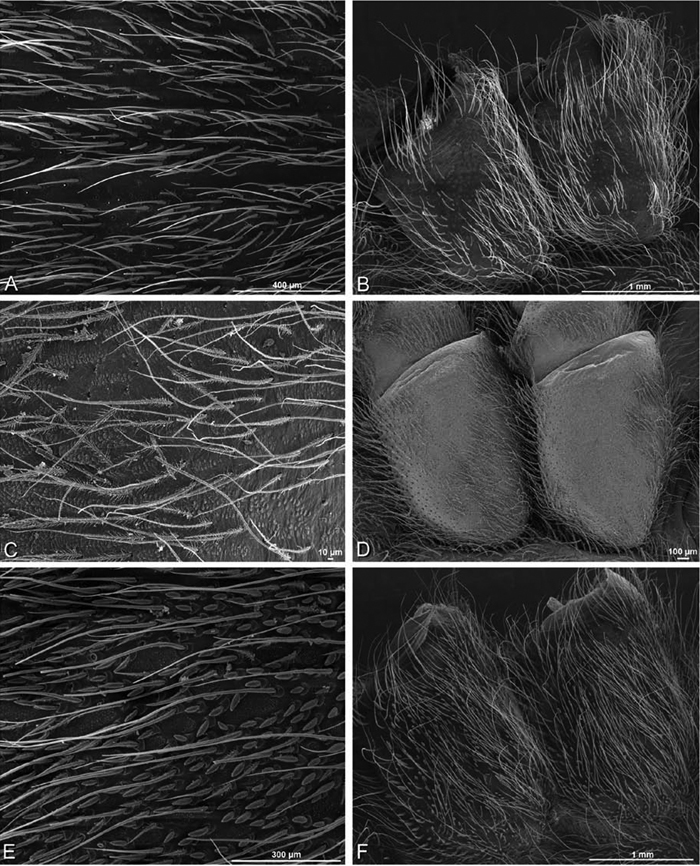
**A–F** *Gandanameno* sp., prosoma and coxae of female, scanning electron micrographs. **A, C, E** detail of setae on prosoma **B, F** right coxae I and II **D** left coxae I and II, image reversed to appear as right coxae **A, B** from Iringa, Tanzania (ZMUC 19970530, ZMUC) **C, D** from Hanover, South Africa (SAM-ENW-B006896/9958, SAM) **E, F** from Eierfontein, Eastern Cape, South Africa (SAM-12823, SAM).

**Figure 54. F54:**
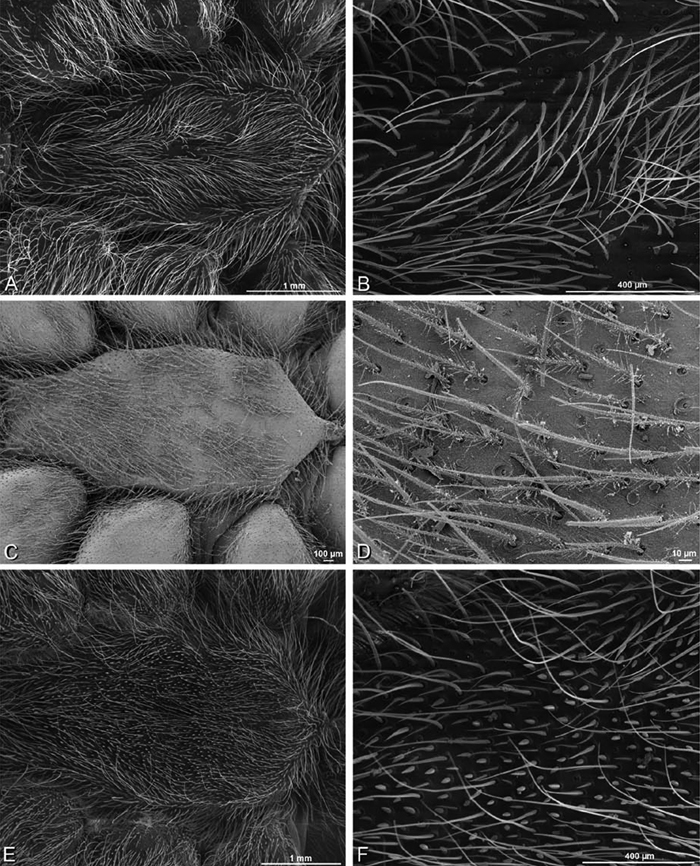
**A–F** *Gandanameno* sp., sternum of female, scanning electron micrographs **A, C, E** overview of sternum **B, D, F** detail of setae on sternum **A, B** from Iringa, Tanzania (ZMUC 19970530, ZMUC) **C, D** from Hanover, South Africa (SAM-ENW-B006896/9958, SAM) **E, F** from Eierfontein, South Africa (SAM-12823, SAM).

**Figure 55. F55:**
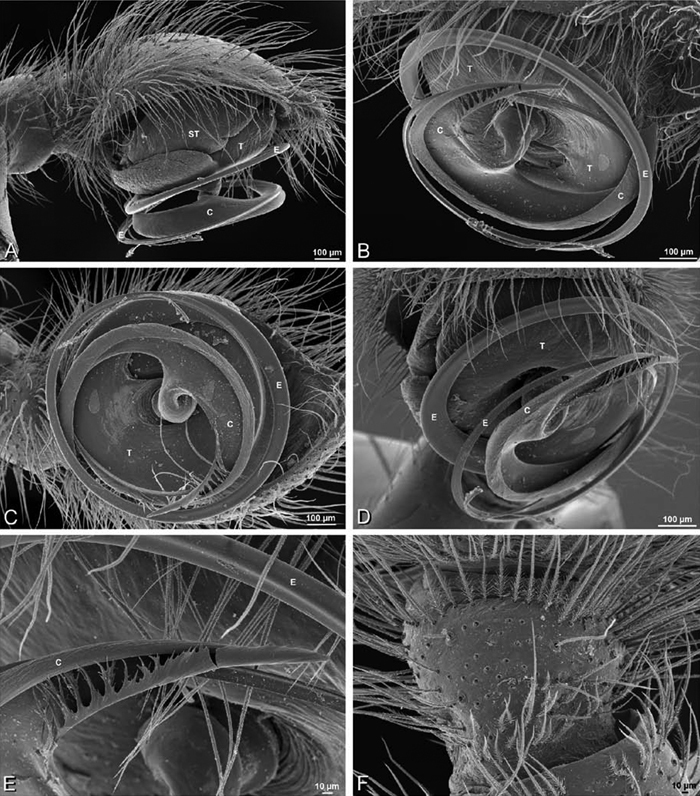
**A–F** *Gandanameno* sp. from Harare, Zimbabwe (AcAT 2005/123, NCA), scanning electron micrographs, right male palp, images reversed to appear as left palp. **A** prolateral view **B** retrolateral view **C** ventral view **D** apical view **E** detail of distal tip of conductor **F** palpal tibia, dorsal view. **C** conductor **E** embolus **ST** subtegulum **T** tegulum.

**Figure 56. F56:**
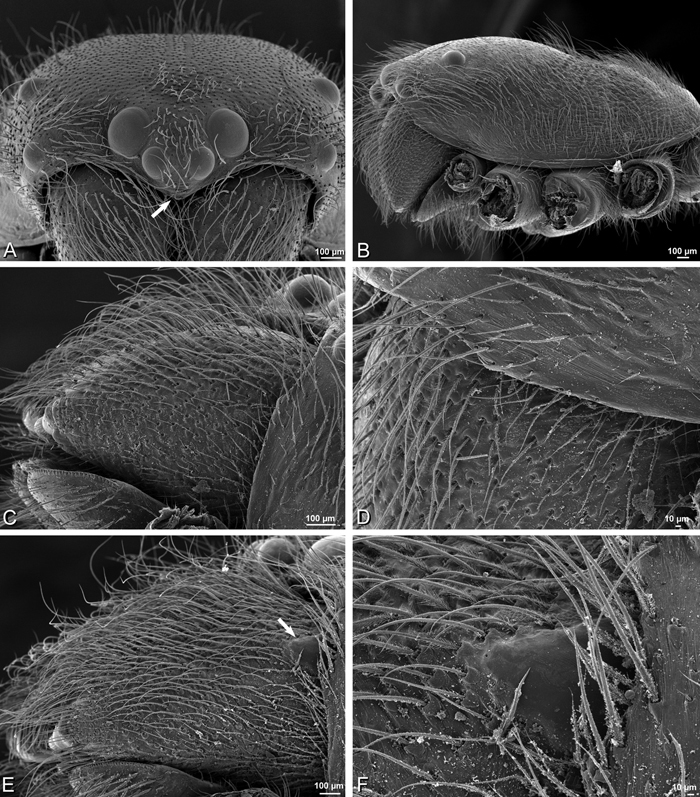
**A–F** *Gandanameno* sp., scanning electron micrographs of prosoma and chelicerae. **A–D** male from Harare, Zimbabwe (AcAT 2005/123, NCA) **E, F** male from Hanover, South Africa (SAM 9465, SAM) **A** prosoma, anterior view, arrow indicates clypeal hood **B** prosoma, lateral view **C, E** left chelicerae, lateral view, arrow in E indicates cheliceral boss **D** detail of left chelicerae showing absence of cheliceral boss **F** detail of left chelicerae showing cheliceral boss.

**Figure 57. F57:**
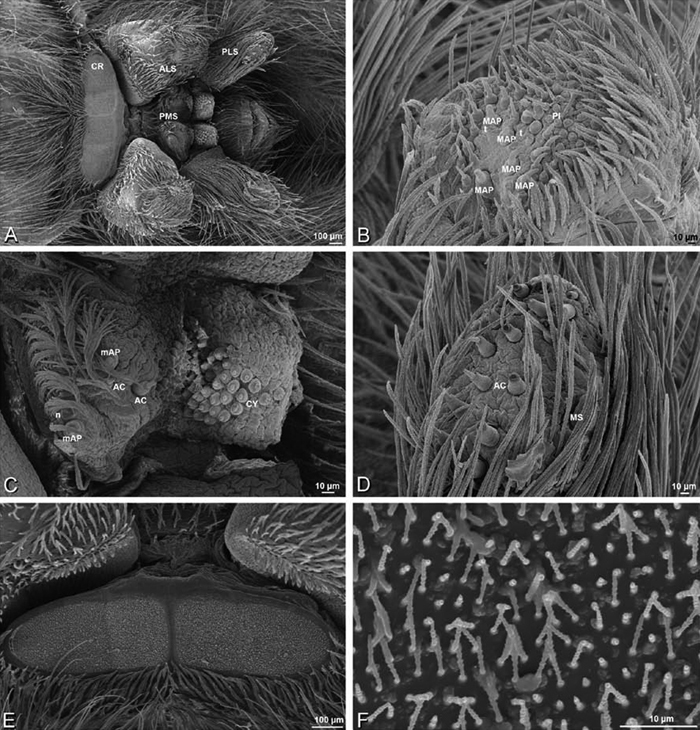
**A–F** *Gandanameno* sp. from Iringa, Tanzania (ZMUC 19970530, ZMUC), scanning electron micrographs of female spinnerets. **A** overview **B** right ALS **C** right PMS **D** left PLS **E** cribellum **F** cribellar spigots. **AC** aciniform gland spigot **ALS** anterior lateral spinneret **CR** cribellum **CY** cylindrical gland spigot **MAP** major ampullate gland spigot **mAP** minor ampullate gland spigot **MS** modified spigot **n** nubbin **PI** piriform gland spigot **PLS** posterior lateral spinneret **PMS** posterior median spinneret **t** tartipore.

**Figure 58. F58:**
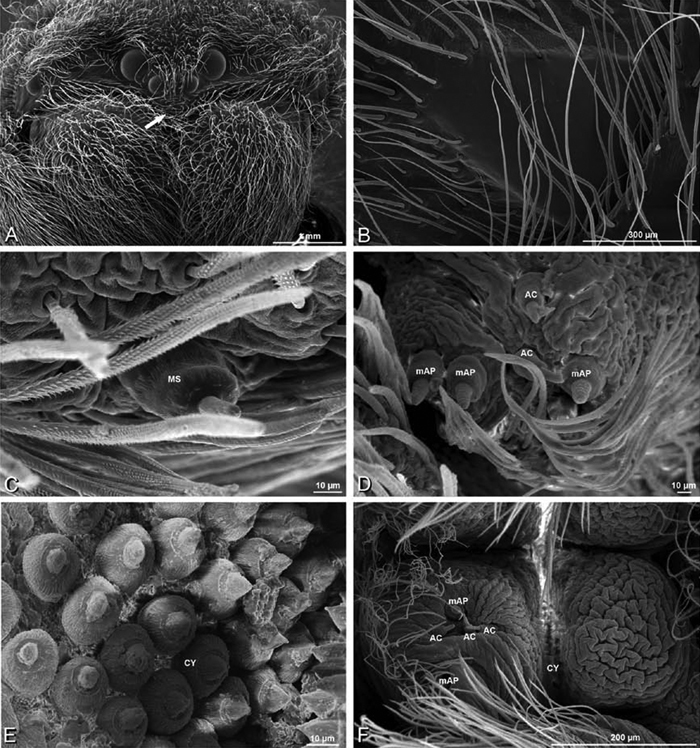
**A–F** *Gandanameno* sp., scanning electron micrographs. **A–E** female from Iringa, Tanzania (ZMUC 19970530, ZMUC) **F** female from Hanover, South Africa (SAM-ENW-B006896/9958) **A, B** prosoma **C–F** details of spinneret spigots **A** anterior view, arrow indicates clypeal hood **B** left cheliceral boss **C** detail of modified spigots on right female PLS **D** detail of spigots on anterior part of right female PMS **E** detail of cylindrical gland spigots on posterior part of left female PMS **F** right PMS. **AC** aciniform gland spigot **CY** cylindrical gland spigot **MAP** major ampullate gland spigot **mAP** minor ampullate gland spigot **MS** modified spigot **n** nubbin **PI** piriform gland spigot **t** tartipore.

**Figure 59. F59:**
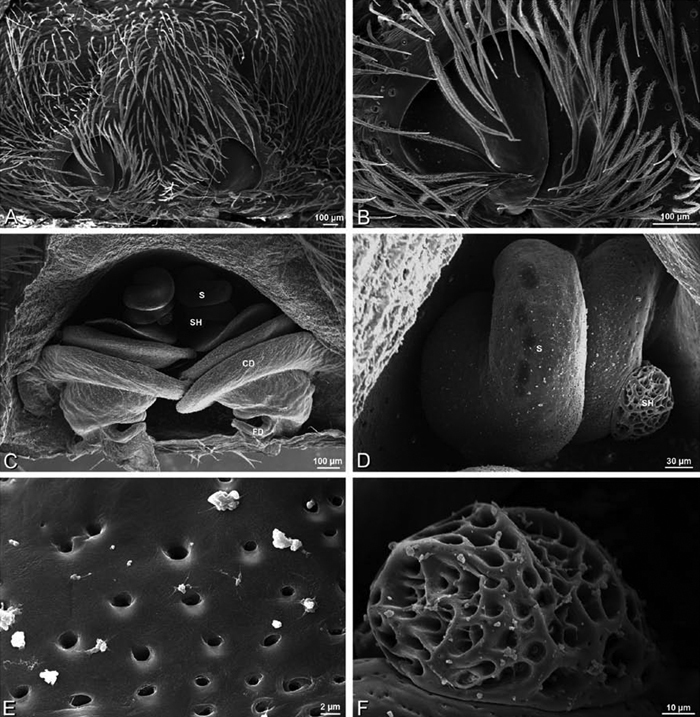
**A–F** Scanning electron micrographs of epigynum and vulva of *Gandanameno* sp. **A, B** from Iringa, Tanzania (ZMUC 19970530, ZMUC) **C–F** from Kommetjie, Cape Town, South Africa (CASENT 9039241, CAS) **A** epigynum, ventral view **B** detail of right copulatory opening, ventral view **C** cleared vulva, dorsal view **D** detail of right spermatheca and spermathecal head **E** detail, right spermatheca **F** detail, right spermathecal head. **CD** copulatory duct **FD** fertilization duct **S** spermatheca **SH** spermathecal head.

**Figure 60. F60:**
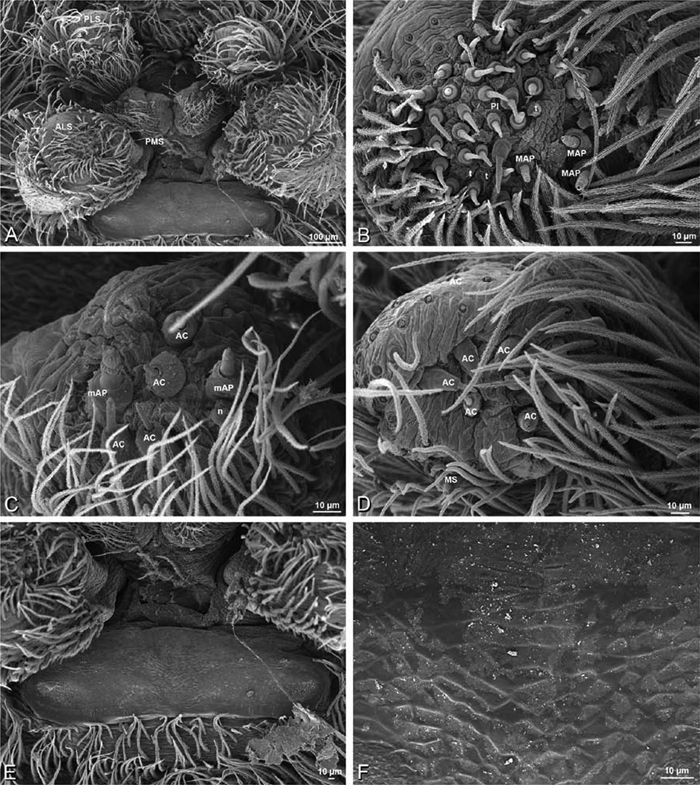
**A–F** *Gandanameno* sp. from Hanover, South Africa (SAM 9465, SAM), scanning electron micrographs of male spinnerets. **A** overview **B** left ALS **C** right PMS **D** left PLS **E** vestigial cribellum **F** detail of vestigial cribellum. **AC** aciniform gland spigot **ALS** anterior lateral spinneret **MAP** major ampullate gland spigot **mAP** minor ampullate gland spigot **MS** modified spigot **PI** piriform gland spigot **PLS** posterior lateral spinneret **PMS** posterior median spinneret **n** nubbin **t** tartipore.

**Figure 61.**
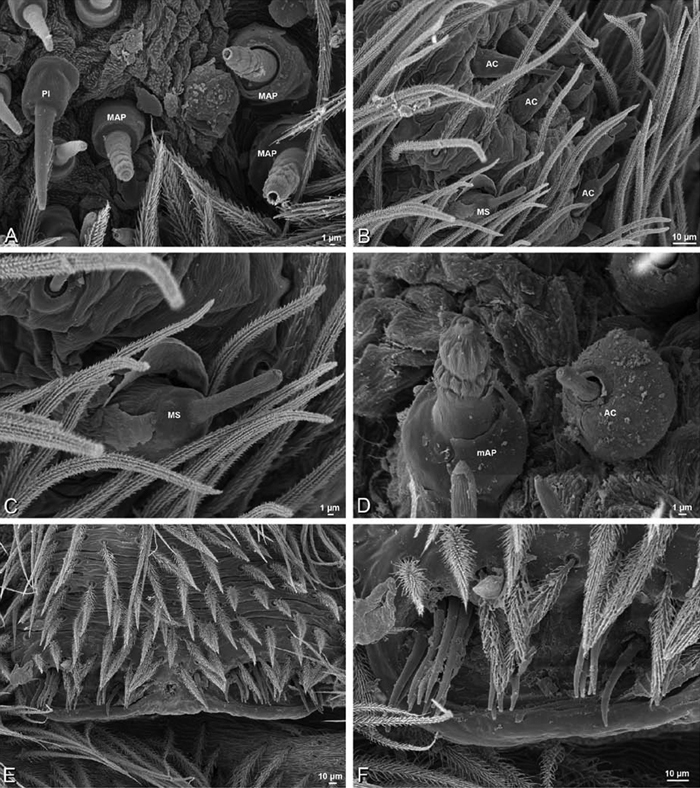
**A–F** *Gandanameno* sp. from Hanover, South Africa (SAM 9465, SAM), scanning electron micrographs of male spinnerets. **A** detail of spigots on left ALS **B** left PLS **C** detail of spigots on left PLS **D** detail of spigots on left PMS **E** epiandrous region **F** detail of epiandrous gland spigots. **AC** aciniform gland spigot **MAP** major ampullate gland spigot **mAP** minor ampullate gland spigot **MS** modified spigot **PI** piriform gland spigot.

#### Loureedia

Miller, Griswold, Scharff, Řezáč, Szűts & Marhabaie gen. n.

urn:lsid:zoobank.org:act:5FEC8D28-5F6F-4E58-A5C2-5EEBD35B0090

http://species-id.net/wiki/Loureedia

##### Type species.

*Eresus annulipes* Lucas, 1857.

##### Circumscription.

*Loureedia* gen. n. is monotypic, containing only the type species. Two species are here synonymized with *Loureedia annulipes*: *Eresus semicanus* Simon, 1908 and *Eresus jerbae* El-Hennawy, 2005. We examined specimens of *Loureedia annulipes* from Israel and type material from the Eugene Simon collection at the MNHN.

##### Etymology.

Named for Lou Reed, leader of the rock band The Velvet Underground from 1965–1970; the gender is feminine.

##### Diagnosis.

**Figure 62.**
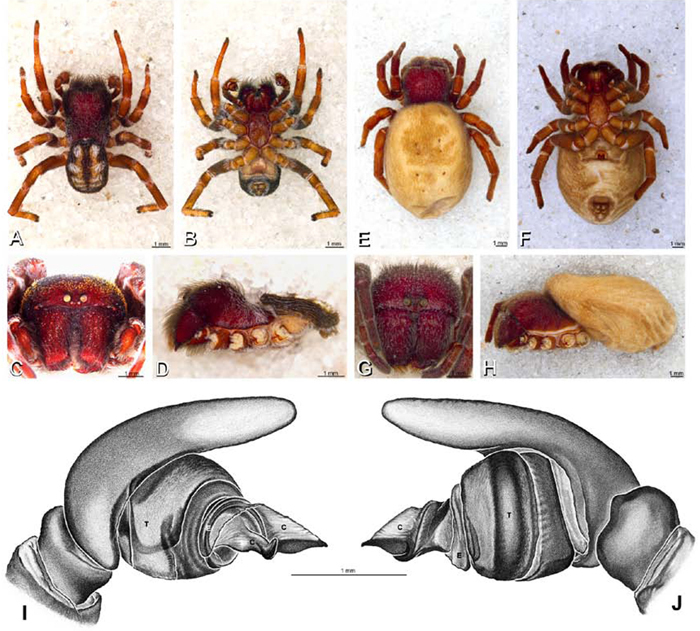
**A–J** *Loureedia annulipes*. **A, B, D** **I, J** male from Haluqim Ridge, Israel (MR008, HUJ) **C** male from Nitzanna village, Israel (MR018, HUJ) **E, F, H** female from Wadi Mashash, Israel (MR019, MR) **G** female from Haluquim, Israel (PET03, MR) **A–D** habitus of male, photomicrographs **E–H** habitus of female, photomicrographs **I–J** illustrations of left male palp **A, E** dorsal view **B, F** ventral view **C, G** anterior view **D, H** lateral view **I** prolateral view **J** retrolateral view. **C** conductor **E** embolus **T** tegulum.

**Figure 63. F63:**
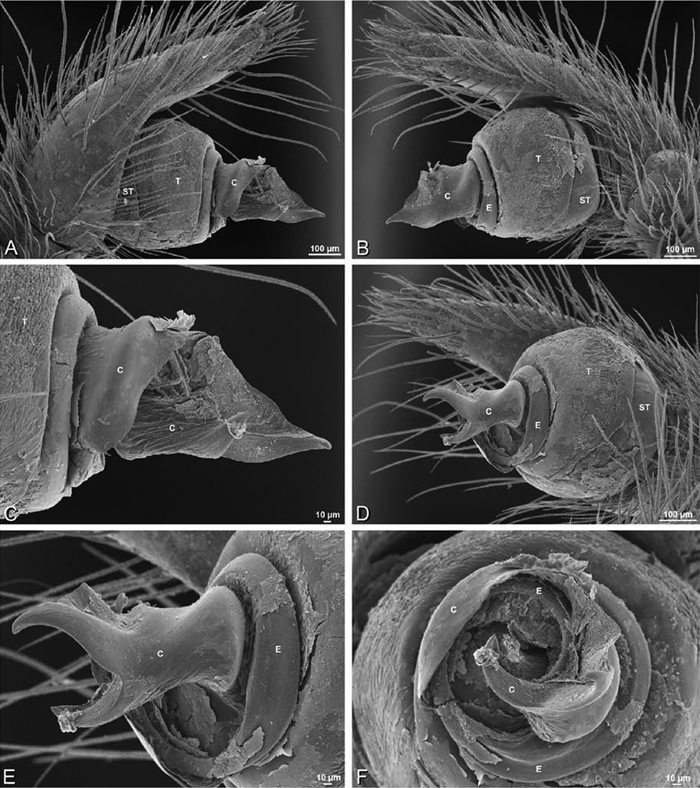
**A–F** *Loureedia annulipes* from Haluqim Ridge, Israel (MR008, HUJ), scanning electron micrographs of right male palp, images reversed to appear as left palp. **A** prolateral view **B** retrolateral view **C** conductor, prolateral view **D** ventral view **E** conductor, ventral view **F** apical view. **C** conductor **E** embolus **ST** subtegulum **T** tegulum.

Distinguished from other eresid genera except *Dorceus*, some *Dresserus*, and *Paradonea splendens* by the cephalic region, which is wider than long (Fig. 9J, L); distinguished from *Dorceus* by the median eye group, which have the PME clearly larger than the AME (AME/PME ca. 0.5; Fig. 9I, K); median eyes small with the PME only slightly larger than the AME in *Dorceus* (Fig. 8E), AME/PME > 0.8; Figs 8E, G, 28A, 29A); distinguished from *Dresserus* by the lack of prominent tubercles bearing the ALE and the palpal conformation, which has a proximal-ventral axis with the helical embolus encircling the distal part (Fig. 63B; obliquely ventral-dorsal in *Dresserus* with the embolus encircling the ventral part, Figs 33I–K, 34A–D); distinguished from *Paradonea splendens* by the subrectangular shape of the cephalic region, which does not overhang the thoracic region posteriorly (Figs 9J, 62A, D; subtrapezoidal, slightly overhanging the thoracic region in *Paradonea splendens*, Fig. 68D). Male further distinguished from other eresids except *Stegodyphus dumicola*, *Stegodyphus tentoriicola*, and *Paradonea striatipes* by the strongly bifid conductor (Fig. 63D, E); separated from these species by several characters including details of the conductor shape, the shape of the cephalic region, the lack of ALE tubercles, and a striking abdominal pattern of white and red patches on a black field (Fig. 1G, H). Female further distinguished by the epigynum with its unique anterior depression and by the compact configuration of the reproductive duct system (Figs 18A, D, 65A, B).

##### Natural history.

Known from Loess desert habitat with low shrubs, often in wadis. They build a simple vertical or inclined burrow lined by silk. The opening is covered by silken sheet camouflaged from above by debris. Signaling threads radiate out from the edges of this roof. Mating occurs in late autumn. Prey remnants are incorporated into the roof of the burrow. Juveniles feed on their mother’s corpse before dispersing (cf. Fig. 3D). Males take approximately 3 years to mature, females one year longer (Martin Forman, personal observation).

#### Loureedia annulipes

(Lucas) comb. n.

http://species-id.net/wiki/Loureedia_annulipes

Figs 1G, H 4I 9I–L 13G–I 18A, D 62 [Fig F63] [Fig F64] [Fig F65] [Fig F66] –67

Eresus annulipes Lucas, 1857: 21.
Eresus semicanus Simon 1908: 83; 1910: 294, fig. 5; El-Hennawy 2004: 28, figs 2A–B, 3A–C, 4A–B. Syn. n.
Stegodyphus annulipes (Lucas, 1857). Kraus and Kraus 1992: 15, 19.
Eresus jerbae El-Hennawy, 2005: 88, figs 1–4. Syn. n.

##### Description.

*Male* (Nitzanna village, Israel, MR018, HUJ): Carapace with scattered white setae; cephalic region subrectangular with broadly rounded posterior margin, wider than long, strongly raised; AME distinctly smaller than PME (AME/PME 0.52), median eyes slightly overlapping on horizontal and vertical axes; ALE tubercles absent; PER as wide as AER (PER/AER 1.01), PLE position on carapace 0.42; clypeal hood forms a nearly 90° angle; fovea moderately deep. Chelicerae slightly excavated mesally, with lateral boss. Legs relatively long with bands of white setae; legs with row of distal ventral macrosetae on metatarsus I–IV, and scattered ventral macrosetae on tibia III–IV and metatarsus and tarsus II–IV, strongest and most numerous on metatarsus and tarsus III–IV. Abdomen dark with pattern of white spots surrounding sigilla and two longitudinal yellow stripes running through sigilla (Figs 1G, H, 9I, J, 62A–D).

Male palp with proximal-distal axis; tegulum subtrapezoidal; conductor and embolus together form apical complex making one helical turn; conductor membranous at prolateral origin, abruptly transitioning dorsally-retrolaterally to heavily sclerotized bifid structure; tegular division longer than embolic division; cymbium with several long prolateral mesosetae (only slightly thicker than normal setae; Figs 13G–I, 62I, J, 63A–F).

**Figure 64. F64:**
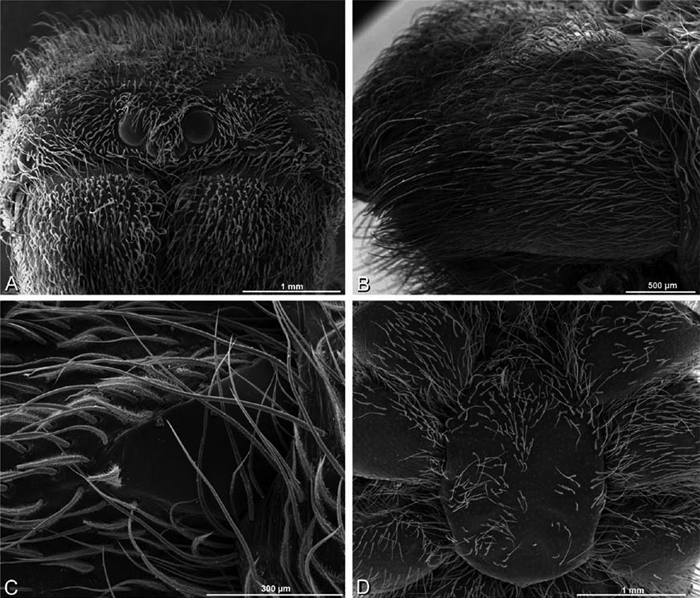
**A–F** *Loureedia annulipes*, scanning electron micrographs of female from from Wadi Mashash, Negev, Israel (MR019, MR), images reversed. **A** prosoma, anterior view **B** chelicera **C** cheliceral boss **D** sternum, ventral view.

*Female* (Haluquim, Israel, PET03, MR): Carapace with many white setae; cephalic region subrectangular, wider than long, moderately raised; AME distinctly smaller than PME (AME/PME 0.47), median eyes slightly overlapping on horizontal and vertical axes; ALE tubercles absent; PER nearly as wide as AER (PER/AER 0.96), PLE position on carapace 0.38; clypeal hood forms acute angle; fovea moderately deep. Chelicerae contiguous mesally, with lateral boss. Legs with scattered white setae; legs with row of distal ventral macrosetae on metatarsus I–IV plus one subdistal ventral macroseta on metatarsus III and scattered ventral macrosetae on tarsus IV. Abdomen without conspicuous white setae (Figs 9K, L, 62E–H, 64A–C).

**Figure 65. F65:**
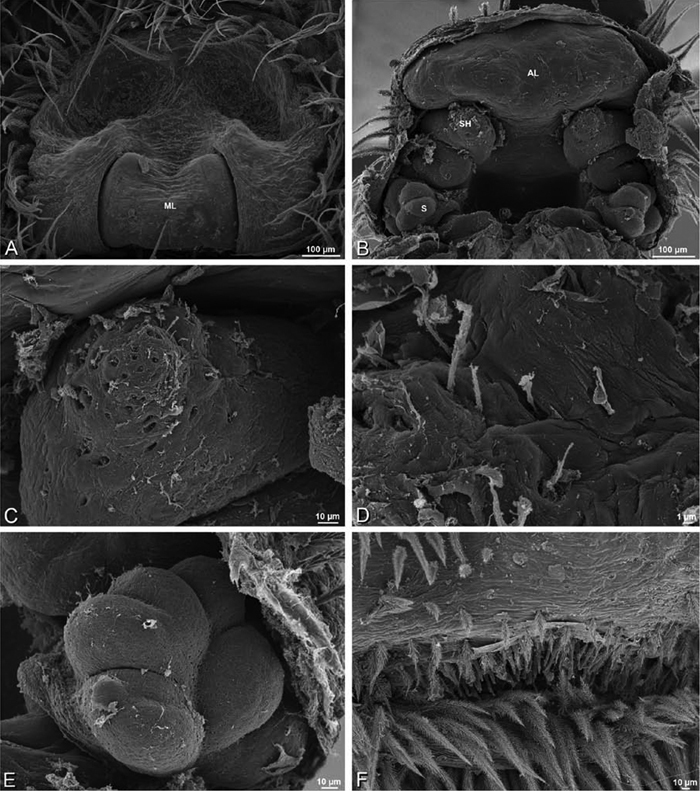
**A–F** *Loureedia annulipes*, scanning electron micrographs. **A–E** vulva of female from Wadi Mashash, Negev, Israel (MR019, MR) **F** male from Haluqim Ridge, Israel (MR008, HUJ) **A** epigynum, ventral view **B** cleared vulva, dorsal view **C, D** detail, left spermathecal head **E** detail, right spermatheca. **F** epiandrous region. **AL** anterior lobe on atrium **ML** median lobe **S** spermatheca **SH** spermathecal head.

Epigynum with slightly bowed slit-like atria occupying ca. the posterior half, anterior part with transverse oblong anterior lobe (Figs 18A, 65A). Vulva with compact, anteriorly converging reproductive duct system with anterior spermathecal head, sinuous duct leading to multilobed spermathecae; with transverse lobe anteriorly (Figs 18D, 65B–E).

##### Spinneret spigot morphology.

**Figure 66. F66:**
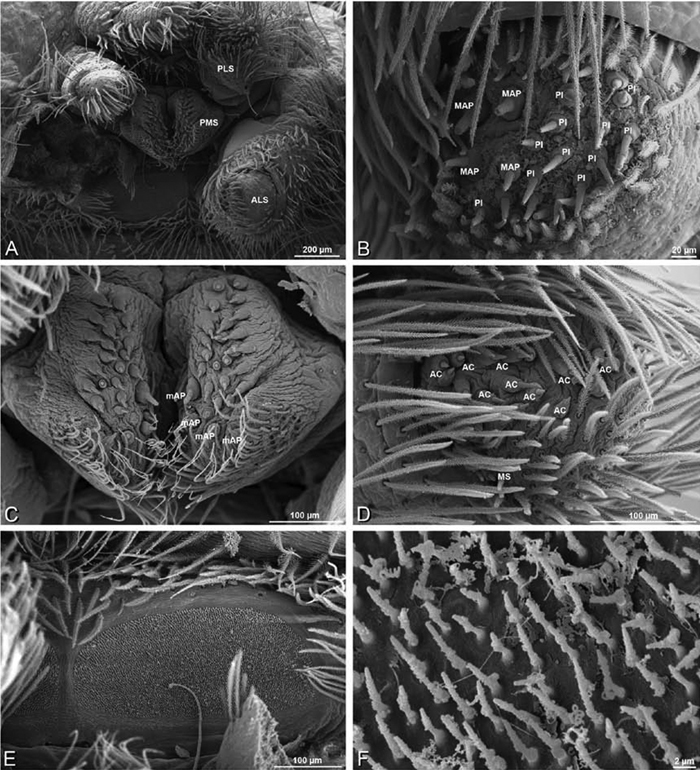
**A–F** *Loureedia annulipes*, female from Wadi Mashash, Negev, Israel (MR019, MR), scanning electron micrographs of spinnerets. **A** overview **B** right ALS **C** left and right PMS **D** right PLS **E** cribellum. **F** cribellar spigots. Unlabeled spigots in **C** thought to be a mixture of aciniform gland spigots and cylindrical gland spigots. **AC** aciniform gland spigot **ALS** anterior lateral spinneret **MAP** major ampullate gland spigot **mAP** minor ampullate gland spigot **MS** modified spigot **PI** piriform gland spigot **PLS** posterior lateral spinneret **PMS** posterior median spinneret.

**Figure 67.**
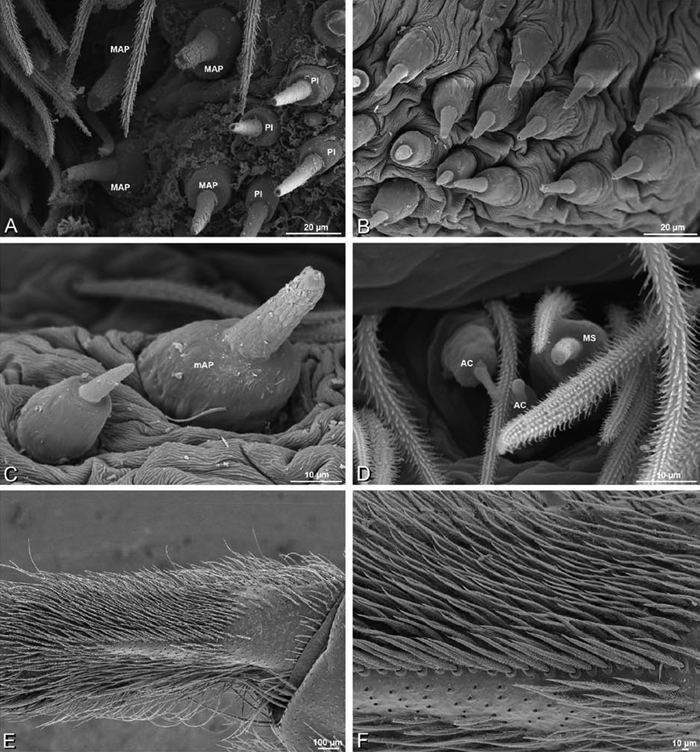
**A–F** *Loureedia annulipes*, female from Wadi Mashash, Negev, Israel (MR019, MR), scanning electron micrographs of spinnerets. **A** detail of spigots on right ALS **B, C** detail of spigots on left PMS **D** modified spigot and flanking aciniform gland spigots on left PLS **E** calamistrum, left metatarsus IV **F** detail, calamistrum seta, left metatarsus IV. Spigots in **B** thought to be a mixture of aciniform gland spigots and cylindrical gland spigots; unlabeled spigot in **C** thought to be either an aciniform gland spigot or a cylindrical gland spigot. **AC** aciniform gland spigot **MAP** major ampullate gland spigot **mAP** minor ampullate gland spigot **MS** modified spigot **PI** piriform gland spigot.

Our female preparation is in poor condition but intact; the male preparation is in very poor condition making it impossible to provide accurate counts, especially on the PMS. Female ALS with at least 5 MAP within and on inner edge of spinning field of more than 23 PI (Figs 66B, 67A); male with MAP and PI, but number can’t be determined. Female PMS with 4 anterior mAP spigots and posterior field of 22 spigots of varying size and shape (Figs 66C, 67B, C); male seems to have fewer spigots than female, suggesting the female may have AC and CY spigots. Female PLS with anterobasal MS with two accompanying AC spigots and distal field of at least 19 AC (Figs 66D, 67D); male appears to have MS and flanking AC, with field of more than 8 AC. Male cribellar plate with no sign of spigots; numerous epiandrous gland spigots present (Fig. 65F).

#### Paradonea

Lawrence

http://species-id.net/wiki/Paradonea

Paradonea Lawrence, 1968: 116. Type species *Paradonea striatipes* Lawrence, 1968.

##### Note.

*Paradonea* contains four recognized species from southern Africa (Fig. 71). We examined specimens (including the primary types) of all four described species and propose one new species. Of these five, only one (*Paradonea variegata*) is known from the female as well as the male. Two species are particularly remarkable in their morphological distinctness: the type species, *Paradonea striatipes*, is large with distinct markings, an enlarged first leg with tibial brush, relatively small palpi with a distinctly bifid conductor, widely spaced median eyes, and PLE set near the front of the carapace; *Paradonea splendens* has a strongly raised cephalic region that occupies most of the cephalothorax and slightly overhangs the thoracic region, but the palpal morphology is fairly typical of Eresidae. Despite our skepticism about the monophyly of this group, we are not proposing nomenclatural changes at this time. We hope that this work may help catalyze new activity in this group, including the discovery of females and the collection of fresh specimens for DNA sequencing. Our phylogenetic analysis includes *Paradonea variegata*, the first *Paradonea* species to be sequenced and the first time *Paradonea* has been placed phylogenetically (Figs 51, S1). We do not offer a diagnosis of the genus but distinguish each species independently in the key and species diagnostic sections.

Spinneret spigot morphology was not investigated for any species in the genus.

#### Paradonea striatipes

Lawrence

http://species-id.net/wiki/Paradonea_striatipes

Figs 10A, B 13J, K 68A–C, G, H 71

Paradonea striat *ipes* Lawrence, 1968: 116–118, figs 2f, 3b.

##### Diagnosis.

**Figure 68.**
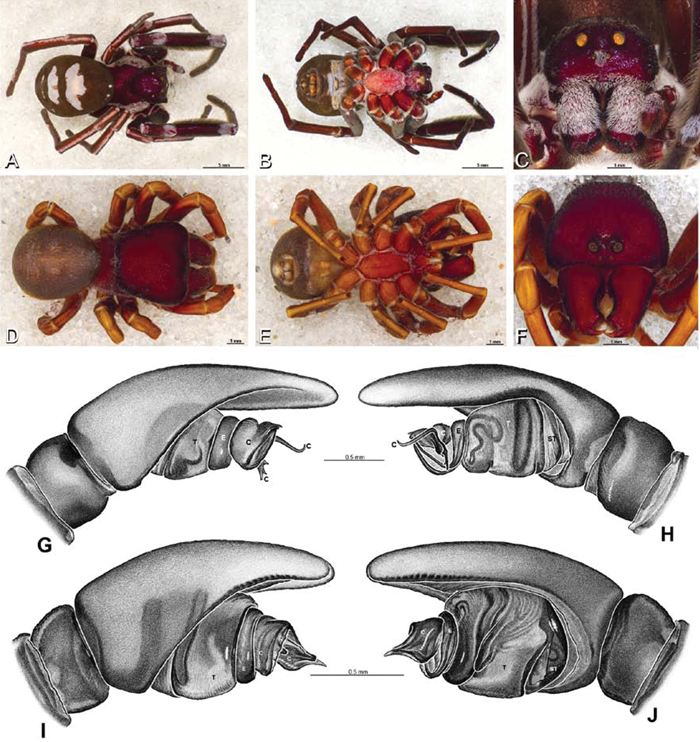
**A–J** males of *Paradonea striatipes* and *Paradonea splendens*. **A–C, G, H** *Paradonea striatipes*, male from Otjivasandu, Namibia (NMN) **D–F, I, J** *Paradonea splendens*, male from Sunnyside, South Africa (C1076, SAM) **A–F** habitus of male, photomicrographs **G–J** illustrations of left male palp **A, D** dorsal view **B, E** ventral view **C, F** anterior view **G, I** prolateral view **H, J** retrolateral view. **C** conductor **E** embolus **ST** subtegulum **T** tegulum.

Distinguished from other Eresidae except *Gandanameno*, some *Dresserus*, *Stegodyphus dumicola*, *Stegodyphus tentoriicola*, and *Loureedia annulipes* by the bifid conductor (Figs 13J, K, 68G, H); distinguished from *Dresserus* and *Gandanameno* by the palpal conformation, which has a proximal-ventral axis with the helical embolus encircling the distal part (obliquely ventral-dorsal in *Dresserus* and *Gandanameno* with the embolus encircling the ventral part, Figs 12G–I, 13D–F, 33I–K, 48A–C); distinguished from *Stegodyphus dumicola*, *Stegodyphus tentoriicola*, and *Loureedia annulipes* by the shape of the conductor branches, which strongly diverge in orientation and feature a curved, spine-like dorsal branch and a broad ventral branch with several small sharp processes (Fig. 68H). Distinguished from other eresids except *Paradonea parva*, *Paradonea presleyi* sp. n., *Seothyra*, and some *Stegodyphus* by the enlarged leg I (Fig. 68A, B), distinguished from *Paradonea parva*, *Paradonea presleyi* sp. n., and *Seothyra* by the presence of a dense brush of setae, especially on the tibia (Fig. 68A, B); distinguished from *Paradonea presleyi* sp. n. and *Stegodyphus* by the separation of the median eyes on the vertical axis (Fig. 10A; broadly overlapping in *Paradonea presleyi* sp. n. and *Stegodyphus*, Figs 11E, 70I). *Paradonea striatipes* has the PLE in a more advanced position (ca. 0.25) than most other eresids (Fig. 11B; *Dresserus*, *Gandanameno*, and *Stegodyphus* may also have the PLE around 0.25). The markings, especially the distribution of white setae, are unique (Fig. 68A–C). The palpi are relatively small proportional to body size compared to other eresids (Fig. 68B).

##### Description.

*Male* (Outjo Namibia, NMBA05700, BMSA): Carapace with broad band of white setae around margin and between AME; cephalic region subtriangular, longer than wide, strongly raised; AME distinctly smaller than PME (AME/PME 0.33), median eyes widely separated on horizontal axis, adjacent on vertical axis; ALE on distinct tubercles; PER much narrower than AER (PER/AER 0.82), PLE position on carapace 0.25; clypeal hood forms acute angle; fovea shallow. Chelicerae with lateral boss, basal three quarters covered in white setae, contiguous mesally. Legs with bands of white setae, especially dorsally along the length of most segments; leg I somewhat thickened and elongated, tibia I with brush of dark setae; with scattered ventral macrosetae on tibia II–IV and metatarsus and tarsus I–IV. Abdomen black with series of irregular transverse stripes formed by thick patches of white setae (Figs 10A, B, 68A–C).

Male palp with proximal-distal axis; tegulum moderately elongate, subtrapezoidal; second loop of sperm duct follows complicated path featuring multiple switchbacks; conductor and embolus together form apical complex making 1.5 helical turns; conductor with conspicuous bifid apophysis arising from retrolateral side, consisting of a spine-like dorsal branch curving distally for nearly 180° and a broad, flattened ventral branch with several small sharp processes along the ventral and distal margins; tegular division longer than embolic division; cymbium with a few mesosetae (only slightly thicker than normal setae) over dorsal to prolateral surface (Figs 12J, K, 68G, H).

*Female*: Unknown.

#### Paradonea splendens

(Lawrence)

http://species-id.net/wiki/Paradonea_splendens

Figs 10C, D 13L 14A 68D–F, I, J 71

Adonea splendens Lawrence, 1936: 146.
Paradonea splendens (Lawrence, 1936). Lawrence 1968: 116.

##### Diagnosis.

Distinguished from other eresids except *Dorceus*, some *Dresserus*, and *Loureedia* gen. n. by the wider than long cephalic region (Figs 10D, 68D), distinguished from these by the subtrapezoidal shape of the cephalic region, much wider anteriorly than posteriorly (Figs 10D, 68D; subrectangular in *Dorceus*, *Dresserus*, and *Loureedia* gen. n. with little difference in width anteriorly to posteriorly, e.g., Figs 8F, 9J); distinguished from other eresids except the male of *Adonea* by the posterior margin of the cephalic region, which slightly overhangs the thoracic region; distinguished from many eresids including *Adonea*, *Dorceus*, *Dresserus*, and *Loureedia* gen. n. by the mesally excavated chelicerae (Fig. 68F; contiguous or only slightly excavated in *Adonea*, *Dorceus*, *Dresserus*, and *Loureedia* gen. n.).

##### Description.

*Male* (Sunnyside, South Africa, C1076, SAM): Carapace with band of white setae around margin of thoracic region, additional white setae form several patches including one on the posterior part of the cephalic region; cephalic region subtrapezoidal, wider than long anteriorly, posterior margin straight, strongly raised, slightly overhanging thoracic region posteriorly; AME distinctly smaller than PME (AME/PME 0.40), median eyes separated on horizontal axis, some overlap on vertical axis; ALE tubercles absent; PER narrower than AER (PER/AER 0.86), PLE position on carapace 0.44; clypeal hood forms acute angle; fovea moderately deep. Chelicerae with lateral boss, excavated mesally. Legs with bands of white setae, especially dorsally along the length of most segments; with row of distal ventral macrosetae on metatarsus I–IV and scattered ventral macrosetae on tibia, metatarsus and tarsus I–IV. Abdomen black with longitudinal stripe with uneven margins formed by a thick concentration of white setae (Figs 10C, D, 68D–F; note description of pattern of setae and color on carapace, legs, and abdomen based in part on specimens other than C1076, which is missing most setae; see Appendix A)

Male palp with proximal-distal axis; tegulum bulbous; conductor and embolus together form apical complex making one helical turn; conductor moderately sclerotized, tip a blunt point; tegular division longer than embolic division; cymbium with several macrosetae over dorsal to prolateral surface (Figs 13L, 14A, 68I, J).

*Female*: Unknown.

#### Paradonea variegata

(Purcell)

http://species-id.net/wiki/Paradonea_variegata

Figs 2G, H 10E–H 14B, C 18B, E 69 71

Adonea variegata Purcell 1904: 137, pl. 10, fig. 7; Lehtinen 1967: 461, figs 457, 459.
Paradonea variegata (Purcell, 1904). Lawrence 1968: 116.

##### Diagnosis.

**Figure 69.**
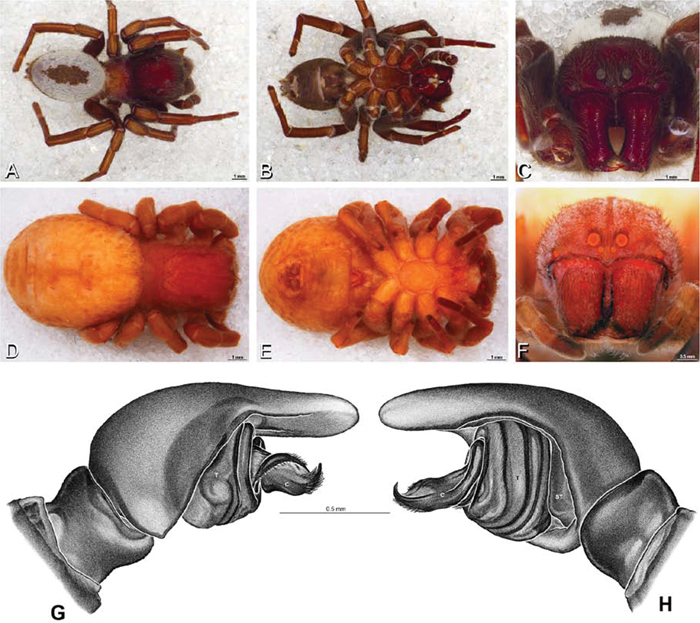
**A–H** *Paradonea variegata*. **A–C, G–H** male from Breekkierie Dunes, Northern Cape, South Africa (C1062, SAM) **D–F** female from Steinkopf, South Africa (ZMB 26964, ZMHB) **A–C** habitus of male, photomicrographs **D–F** habitus of female photomicrographs **G–H** illustrations of left male palp **A, D** dorsal view **B, E** ventral view **C, F** anterior view **G** prolateral view **H** retrolateral view. **C** conductor **ST** subtegulum **T** tegulum.

**Figure 70.**
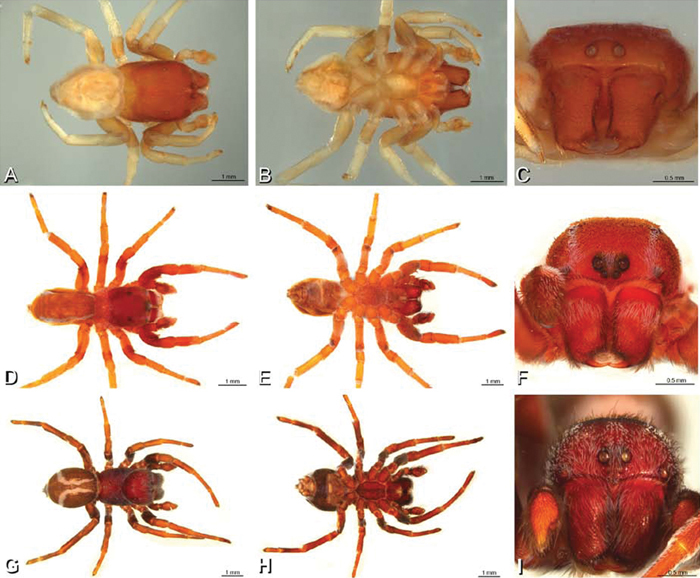
**A–I** males of *Paradonea parva* and *Paradonea presleyi* sp. n., habitus, photomicrographs. **A–F** *Paradonea parva* **A–C** male holotype from junction of Marico and Crocodile Rivers, South Africa (B3701, SAM) **D–F** male from 4 km N of Hopetown, Northern Cape, South Africa (AcAT 97/988, NCA). **G–I** male holotype of *Paradonea presleyi* sp. n. from Falcon College, Zimbabwe (CASENT 9039236, CAS) **A, D, G** dorsal view **B, E, H** ventral view **C, F, I** anterior view.

Male distinguished from other eresids except *Paradonea presleyi* sp. n. by the conductor, which bears a helical ridge fringed with distinct papillae (Figs 14B, C, 69G, H); distinguished from *Paradonea presleyi* sp. n. by the mesally excavated chelicerae (Fig. 69C; contiguous in *Paradonea presleyi* sp. n., Fig. 70I), the dorsal abdominal pattern (Fig. 69A), the median eye group, which has only slight overlap on the vertical axis (Figs 10E, 69C; significant overlap in *Paradonea presleyi* sp. n., Fig. 70I), and the relatively more narrow and long shape of the conductor (Fig. 14B, C; compare with Fig. 14J, L). Female distinguished from other eresids except members of the *Eresus sandaliatus* group by the large, bulbous spermathecal head (Fig. 18E); distinguished from the *Eresus sandaliatus* group by the larger size difference between the AME and PME (AME/PME ca. 0.5 in *Paradonea variegata*, Fig. 69F; >0.6 in *Eresus sandaliatus* group, Fig. 9C) and by the overall lighter color (Fig. 69D; compare with Fig. 43E). Note that interpretation of the female reproductive duct characters for *Paradonea variegata* is based on light microscopy, not SEM, and must therefore be regarded as tentative.

##### Description.

*Male* (Breekkierie Dunes, South Africa, C1062, SAM): Carapace with some white setae, especially around margin of thoracic region, cephalic region subtriangular, longer than wide, moderately raised; AME distinctly smaller than PME (AME/PME 0.52), median eyes adjacent on horizontal axis, some overlap on vertical axis; ALE tubercles absent; PER much narrower than AER (PER/AER 0.86), PLE position on carapace 0.31, clypeal hood forms a slightly less than 90° angle; fovea shallow. Chelicerae with lateral boss, excavated mesally. Legs with some white setae; with row of distal ventral macrosetae on metatarsus I–IV and scattered ventral macrosetae on metatarsus and tarsus I–IV. Dorsal surface of abdomen thickly covered in white setae except for central irregular oblong dark patch (Figs 10E, F, 69A–C).

Male palp with proximal-distal axis; tegulum bulbous; conductor and embolus together form apical complex running more or less distally; conductor moderately sclerotized with helical ridge fringed with distinct papillae, hooked distally; tegular division longer than embolic division; cymbium with several prolateral macrosetae (Figs 14B, C, 69G, H).

*Female* (Steinkopf, South Africa, ZMB 26964, ZMHB): Carapace with patches of white setae; cephalic region subtrapezoidal, longer than wide, moderately raised; AME distinctly smaller than PME (AME/PME 0.48), median eyes with some overlap on horizontal axis, some overlap on vertical axis; ALE on small tubercles; PER slightly narrower than AER (PER/AER 0.93), PLE position on carapace 0.31; clypeal hood forms acute angle; fovea moderately deep. Chelicerae contiguous mesally, with lateral boss. Legs without conspicuous white setae; legs with row of distal ventral macrosetae on metatarsus I–IV and numerous ventral macrosetae on metatarsus and tarsus II–IV. Abdomen with numerous white setae (Figs 10G, H, 69D–F).

Epigynum with slightly converging slit-like atria copulatory converging slits occupying ca. the posterior half, median lobe subtrapezoidal, nearly as long as wide, deeply recessed within epigynum (Fig. 18B). Vulva with bulbous lobes slightly converging anteriorly (presumably bearing the spermathecal heads) and multilobed spermathecae posteriorly (Fig. 18E).

##### Natural history.

Known from savanna and semiarid desert. They build silken tubes under stones or under shrubs. Sometimes, spiders build a round web approximately 10 cm in diameter that may be covered with sand and herbal debris. Juveniles feed on their mother’s corpse before dispersing (cf. Fig. 3D). Adults appear around December, juveniles disperse in October (Martin Forman, personal observation).

#### Paradonea parva

(Tucker)

http://species-id.net/wiki/Paradonea_parva

Figs 14D–I 70A–F 71

Adonea parva Tucker, 1920: 451, pl. 28, fig. 2.
Paradonea parva (Tucker, 1920). Lawrence 1968: 116.

##### Diagnosis.

Distinguished from other eresids except *Seothyra* and some *Stegodyphus* by the embolic division, which is much longer than the tegular division (Fig. 14D–I); distinguished from *Seothyra* by the median eye group, which have the PME clearly larger than the AME (AME/PME ca. 0.5, Fig. 70F; median eyes small and subequal *Seothyra*, Fig. 10K); distinguished from *Stegodyphus* by having the PLE position >0.33 (<0.28 in *Stegodyphus*). By contrast with most other eresids (except *Paradonea striatipes*, *Paradonea presleyi* sp. n., *Seothyra*, and some *Stegodyphus*), *Paradonea parva* has a slightly enlarged leg I (Fig. 70D, E).

##### Note.

The holotype specimen is bleached and damaged (Figs 14D–F, 70A–C). The description is based mostly on better preserved, putatively conspecific individuals. However, there are morphological differences including carapace shape (compare Fig. 70C with Fig. 70F) and the shape of the palpal conductor (compare Fig. 14E with Fig. 14H), which could be taken to mean that there is more than one species in this complex. But for now, we attribute these differences to preservation artifacts. The discovery and examination of more fresh specimens would be helpful in resolving this question. For now, we present photographs of both the holotype (Figs 14D–F, 70A–C) and a more well-preserved specimen (Figs 14G–I, 70D–F).

##### Description.

*Male* (4 km N of Hopetown, South Africa, AcAT 97/988, NCA): Carapace with white setae concentrated in thoracic region and posterior of cephalic region, and forming two longitudinal lines connecting the lateral eyes; cephalic region subtriangular, longer than wide, slightly raised; AME distinctly smaller than PME (AME/PME 0.45), median eyes slightly overlapping on horizontal and vertical axes; ALE tubercles absent; PER slightly narrower than AER (PER/AER 0.94), PLE position on carapace 0.35; clypeal hood forms a slightly less than 90° angle; fovea indistinct. Chelicerae slightly excavated mesally, with lateral boss. Legs with patches and bands of white setae; leg I slightly thickened; with row of distal ventral macrosetae on metatarsus I–IV, a few scattered ventral macrosetae on tarsus I–IV and metatarsus III–IV; leg I slightly thickened. Abdomen with two longitudinal stripes of white hairs ectal to sigilla (Fig. 70D–F).

Male palp with proximal-distal axis; tegulum bulbous; conductor and embolus together form apical complex running more or less distally; conductor moderately sclerotized, broad with longitudinal ridges, tip blunt, embolic division much longer than tegular division; cymbium with several prolateral, fewer retrolateral macrosetae (Fig. 14G–I).

*Female*: Unknown.

**Figure 71.**
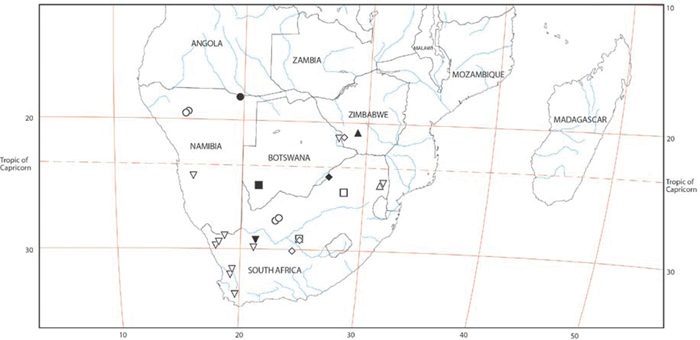
Distribution of *Paradonea* species. Circles, *Paradonea striatipes*; squares, *Paradonea splendens*; inverted triangles, *Paradonea variegata*; diamonds, *Paradonea parva*; triangles, *Paradonea presleyi* sp. n. Primary type localities with all black symbols, those for other localities with white center.

#### Paradonea presleyi

Miller, Griswold, Scharff, Řezáč, Szűts & Marhabaie sp. n.

urn:lsid:zoobank.org:act:29D16AC7-AB63-4125-8CD7-9B0A7EE8456B

http://species-id.net/wiki/Paradonea_presleyi

Figs 14J–L 70F–I 71

##### Types.

Holotype male from Falcon College, Zimbabwe, 20°18'S, 29°E, 10 November 1990 (V. & B. Roth, CASENT 9039236, deposited in CAS); paratype male from Kruger National Park, South Africa, 29 November 2005, pit traps PCZ (K. Harris, AcAT 2008/4480, deposited in NCA).

##### Etymology.

A patronymic in honor of Elvis Aaron Presley, king of rock and roll and subject of innumerable black velvet paintings.

##### Diagnosis

**.** Distinguished from other eresids except *Stegodyphus* by the median eye group, which has large, subequal eyes (AME/PME > 0.6) with clear separation on the horizontal axis and significant overlap on the vertical axis (Fig. 70I); distinguished from *Stegodyphus* by the short, slightly obtuse clypeal hood (long and acute in *Stegodyphus*, Fig. 11A, E, I). Male distinguished from other eresids by the distinctive dorsal abdominal pattern (Fig. 70G). Further distinguished from other eresids except *Paradonea variegata* by the conductor, which bears a helical ridge fringed with distinct papillae (Fig. 14J, L); distinguished from *Paradonea variegata* by the contiguous chelicerae (Fig. 70I; mesally excavated in *Paradonea variegata*, Fig. 69C), by the median eye group, which has significant overlap on the vertical axis (Fig. 70I; slight overlap in *Paradonea variegata*, Fig. 69C), and by the proportions of the conductor, which are wider and shorter than in *Paradonea variegata* (Fig. 14J, L; compare with 14B, C).

##### Description.

*Male* (Kruger National Park, South Africa, AcAT 2008/4480, NCA): Carapace with white setae concentrated in thoracic region and eye region; cephalic region semicircular, wider than long, moderately raised; AME smaller than PME (0.57), median eyes slightly separated on horizontal axis, significant overlap on vertical axis, ALE tubercles absent, PER width narrower than AER width (0.87), PLE position on carapace 0.29; clypeal hood forms a slightly more than 90° angle; fovea shallow. Chelicerae contiguous mesally, with lateral boss. Legs with patches and longitudinal bands of white setae; femur I slightly thickened with thick brush of dark setae; with row of distal ventral macrosetae on metatarsus I–IV, a few scattered ventral macrosetae on tarsus I–IV and metatarsus II–IV. Dorsum of abdomen with two longitudinal stripes of white hairs more or less parallel anteriorly, diverging posteriorly, then connected by transverse portion, median part medium brown, ectal and posterior part dark brown (Fig. 70G–I).

Male palp with proximal-distal axis; tegulum subrectangular; conductor and embolus together form apical complex running more or less distally; conductor moderately sclerotized, broad with helical ridge fringed with distinct papillae, hooked distally; tegular division slightly longer than embolic division; cymbium with several prolateral macrosetae (Fig. 14J–L).

*Female*: Unknown.

#### Seothyra

Purcell

http://species-id.net/wiki/Seothyra

Seothyra Purcell 1903: 31; Lehtinen 1967: 264; Dippenaar-Schoeman 1990: 136. Type species *Seothyra schreineri* Purcell, 1903.

##### Note.

*Seothyra* contains 13 recognized species from southern Africa and was revised by Dippenaar-Schoeman (1990). We examined specimens of *Seothyra henscheli* Dippenaar-Schoeman, 1991 from Namibia. Our species identification was based on the recent revision; we did not seek to examine type material. See *Dorceus* for note on phylogenetic relationships.

##### Diagnosis.

**Figure 72.**
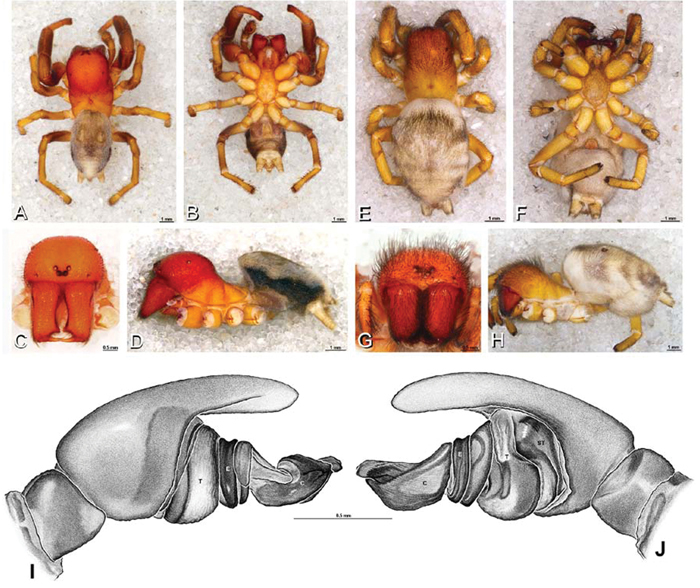
**A–J** *Seothyra henscheli*. **A–D, I–J** male from Gobabeb Station, Namibia (SMN 40828, NMN) **E–H** female from Kuiseb River, Gobabeb, Namibia (SMN 46627, NMN) **A–D** habitus of male, photomicrographs **E–H** habitus of female, photomicrographs **I, J** illustrations of left male palp **A, E** dorsal view **B, F** ventral view **C, G** anterior view **D, H** lateral view **I** prolateral view **J** retrolateral view. **C** conductor **E** embolus **ST** subtegulum **T** tegulum.

**Figure 73. F73:**
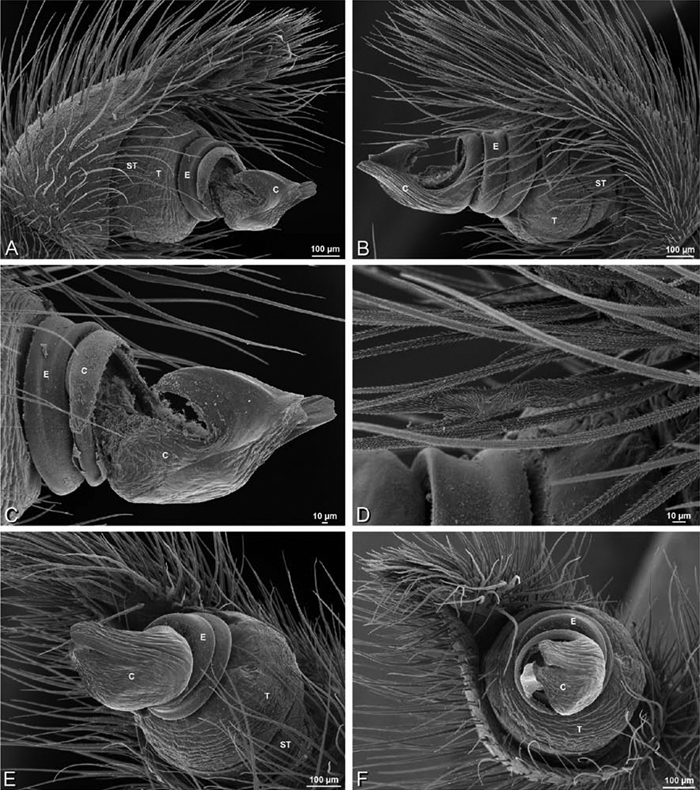
**A–F** *Seothyra henscheli* from Gobabeb Station, Namibia (SMN 40828, NMN), scanning electron micrographs of right male palp, images reversed to appear as left palp. **A** prolateral view **B** retrolateral view **C** detail of embolic division, prolateral view **D** detail of modified setae on retrolateral side of cymbium. **E** ventral view **F** apical view. **C** conductor **E** embolus **ST** subtegulum **T** tegulum.

**Figure 74. F74:**
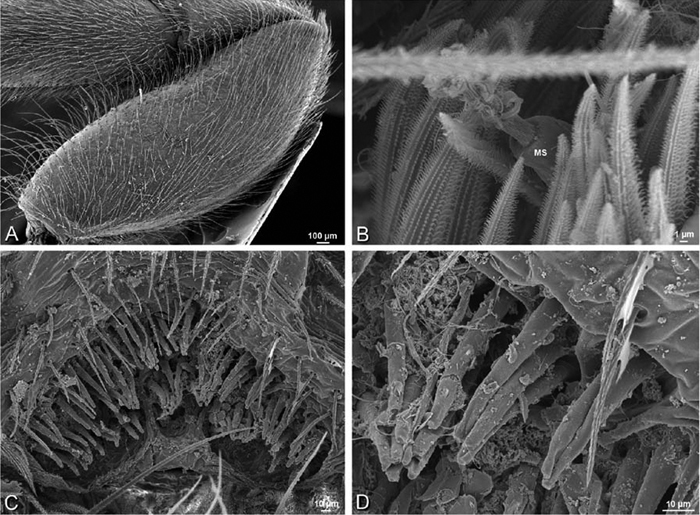
**A–D** *Seothyra henscheli* from Gobabeb Station, Namibia (SMN 40828, NMN), scanning electron micrographs of male legI spinnerets, and epiandrous region. **A** femurI retrolateral view **B** detail of modified spigot on left male PLS **C** epiandrous region **D** detail of epiandrous gland spigots. **MS** modified spigot.

Distinguished from other eresid genera except *Dorceus* by the small eyes subequal in size (AME/PME > 0.7, Figs 10I, K, 72C, G), and the long, extensible ALS contrasting with reduced PLS (Fig. 72D, although ALS may be retracted and therefore not look so long). Male distinguished from *Dorceus* by the enlarged leg I (Figs 72A, B, 74A; legs I and II subequal in *Dorceus*, Fig. 26A), and by the form of the conductor, which is highly variable and elaborate and usually longer than the tegular division (Figs 15A, B, 72I, J, 73A–F; a simple spiral or L-shape hook and shorter than the tegular division in *Dorceus*, Figs 12D, E, 26I, J; see El-Hennawy 2002). Female distinguished by the median lobe of the epigynum, which is clearly longer than wide with a central constriction (Figs 18C; 76A; wider than long with more or less straight, converging lateral margins in *Dorceus*, Figs 16B, 29C) and by the lack of ampullate gland spigots on the ALS (Fig. 77B).

##### Distinguishing species.

*Seothyra* was revised by Dippenaar-Schoeman in 1990.

##### Natural history.

Known from semi-stabilized sand dunes in semiarid desert (Dippenaar-Schoeman 1990). They build a simple straight to curved 5–15 cm deep burrow (Fig. 4H). The opening is covered by two or four lobed silk flaps covered with sand, resembling a hoof print (Fig. 4G; Lubin 1989; Peters 1992a). Prey remnants are placed at the bottom of the burrow. The spider positions itself upside down under the burrow cover (Filmer 1995). Prey are mainly ants (Dippenaar-Schoeman 1990). Eggs hatch at the beginning of summer. Juveniles feed on their mother’s corpse before dispersing (cf. Fig. 3D). Mating occurs in April and May. Males are active during the day, mimicking *Camponotus* ants and mutillid wasps in behavior and appearance (Henschel 1989).

#### Seothyra henscheli

Dippenaar-Schoeman

http://species-id.net/wiki/Seothyra_henscheli

**Figure F77:**
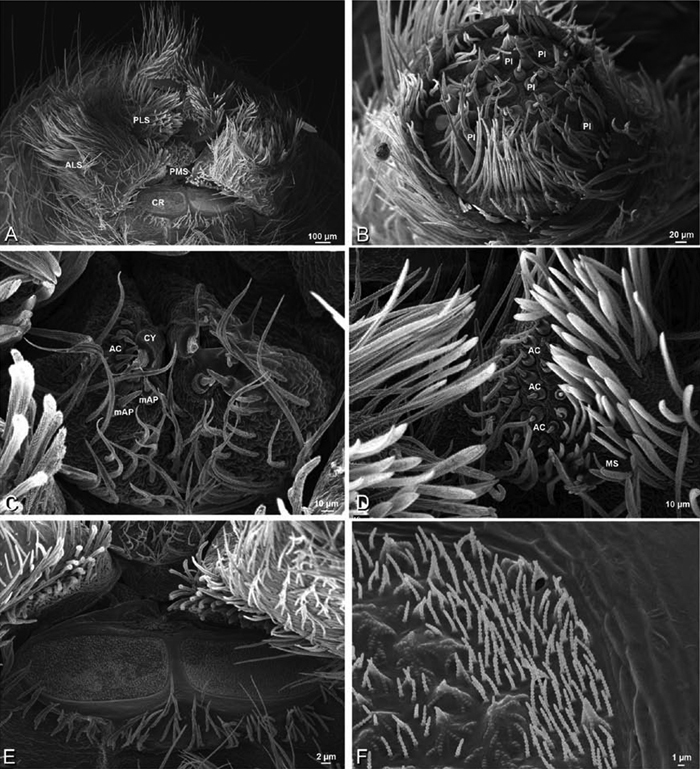
**Figure 77. A–F** *Seothyra henscheli* from Kuiseb River, Gobabeb, Namibia (SMN 46627, NMN), scanning electron micrographs of female spinnerets. **A** overview **B** left ALS **C** left and right PMS **D** right PLS **E** cribellum **F** cribellar spigots. **AC** aciniform gland spigot **ALS** anterior lateral spinneret **CR** cribellum **CY** cylindrical gland spigot **mAP** minor ampullate gland spigot **MS** modified spigot **PI** piriform gland spigot **PLS** posterior lateral spinneret **PMS** posterior median spinneret.

Figs 10I–L 15A–C 18C, F 72 [Fig F73] [Fig F74] [Fig F75] [Fig F76] [Fig F77] –78

Seothyra henscheli Dippenaar-Schoeman, 1991: 156, figs 15, 26, 37, 67–70.

##### Description.

*Male* (Gobabeb Station, Namibia, SMN 40828, NMN): Carapace without conspicuous white setae; cephalic region subrectangular, longer than wide, strongly raised; AME slightly larger than PME (AME/PME 1.08), median eyes adjacent on horizontal axis, some overlap on vertical axis; ALE tubercles absent; PER slightly wider than AER (PER/AER 1.18), PLE position on carapace 0.35; clypeal hood forms a slightly less than 90° angle; fovea deep. Chelicerae with small lateral boss, excavated mesally, with tooth bearing a row of four denticles adjacent to the base of the fang. Legs without conspicuous white setae; femur, patella and tibia I conspicuously thickened; with row of distal ventral macrosetae on metatarsus II–IV plus a distal ventral macroseta on tibia III and scattered ventral macrosetae on metatarsus and tarsus III–IV and tibia IV. Abdomen with many white setae dorsally (Fig. 72A–D).

Male palp with proximal-distal axis; tegulum bulbous; conductor and embolus together form apical complex making about two helical turns; conductor increasingly sclerotized distally terminating in a recurved hook; embolic division longer than tegular division; cymbium with several prolateral and retrolateral macrosetae, some macrosetae arising from retrolateral modified with subbasal enlargement (Figs 15A–C, 72I, J, 73A–F).

**Figure 75. F75:**
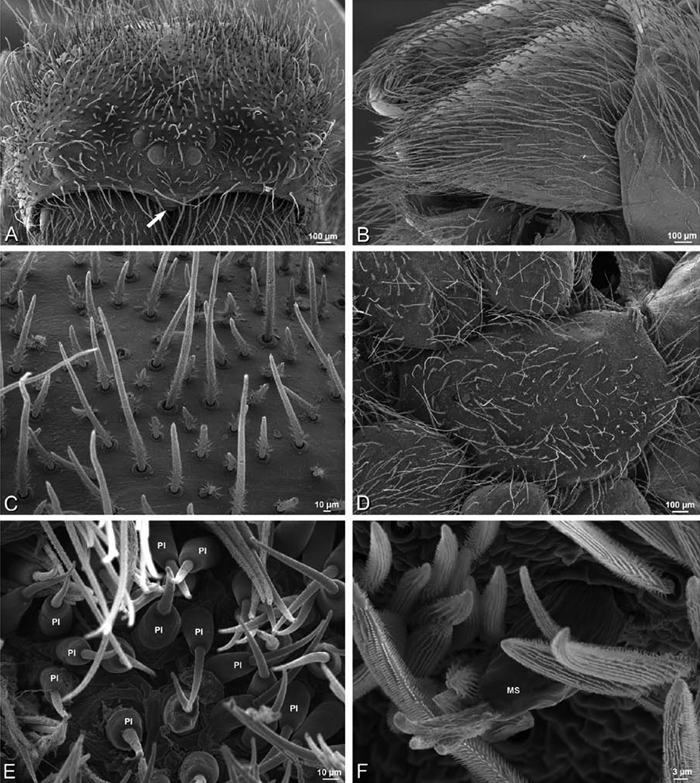
**A–F** *Seothyra henscheli* from Kuiseb River, Gobabeb, Namibia (SMN 46627, NMN), scanning electron micrographs of female prosoma and spinnerets. **A** prosoma, anterior view, arrow indicates clypeal hood **B** left chelicerae, ectal view **C** detail of setae on prosoma **D** sternum, ventral view **E** detail of piriform gland spigots on left female ALS **F** detail of modified spigot on right female PLS. **MS** modified spigot **PI** piriform gland spigot.

*Female* (Gobabeb, Namibia, SMN 46627, NMN): Carapace with many stiff dark setae in the cephalic region and scattered white setae, especially in the thoracic region; cephalic region subrectangular, longer than wide, moderately raised; AME slightly larger than PME (AME/PME 1.05), median eyes adjacent on horizontal axis, some overlap on vertical axis; ALE tubercles absent; PER slightly wider than AER (PER/AER 1.09), PLE position on carapace 0.38; clypeal hood forms a slightly obtuse angle; fovea moderately deep. Chelicerae contiguous mesally, with small lateral boss. Legs without conspicuous white setae; legs with row of distal ventral macrosetae on metatarsus II–IV, a few scattered ventral macrosetae on tarsus II and numerous ventral macrosetae on metatarsus and tarsus III–IV. Abdomen with white setae mostly around the margin (Figs 72E–H, 75A, B).

**Figure 76. F76:**
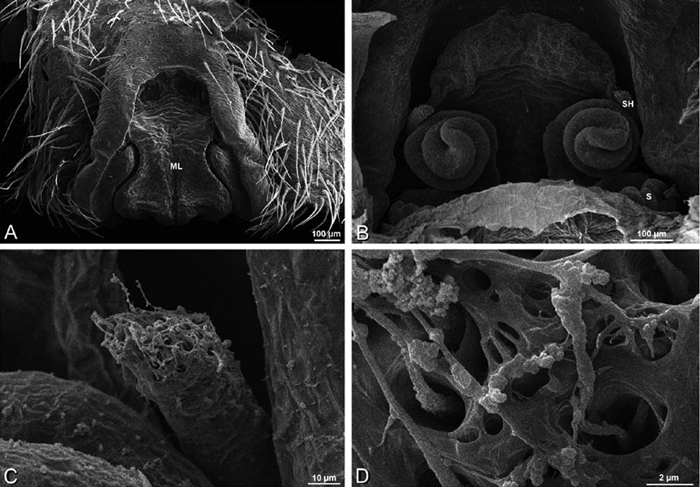
**A–F** *Seothyra henscheli* from Sout Rivier, Namibia (CASENT 9039242, CAS), scanning electron micrographs of epigynum **A** epigynum, ventral view **B** vulva, dorsal view **C, D** spermathecal head. **ML** median lobe **S** spermatheca **SH** spermathecal head.

Epigynum with sinuous slit-like atria occupying ca. half the total length, median lobe margin defined anteriorly and laterally by a ridge (Figs 18G, 76A). Vulva with tightly-wound sinuous stalk leading to spermathecal head at anterior end, multilobed spermathecae at posterior end (Figs 18F, 76B–D).

**Figure 78.**
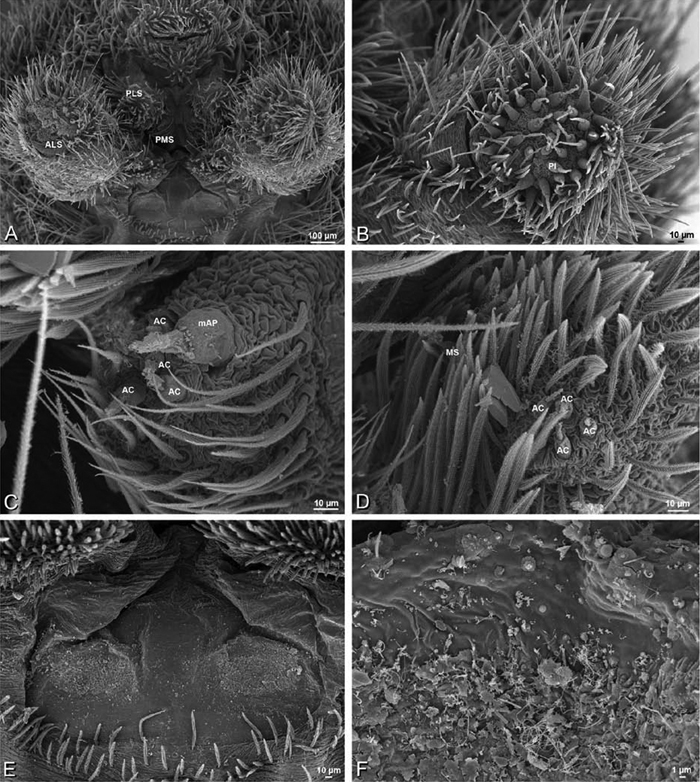
**A–F** *Seothyra henscheli* from Gobabeb Station, Namibia (SMN 40828, NMN), scanning electron micrographs of male spinnerets. **A** overview **B** right ALS **C** left PMS **D** left PLS **E** vestigial cribellum **F** detail of vestigial cribellum. **AC** aciniform gland spigot **ALS** anterior lateral spinneret **mAP** minor ampullate gland spigot **MS** modified spigot **PI** piriform gland spigot **PLS** posterior lateral spinneret **PMS** posterior median spinneret.

##### Spinneret spigot morphology.

Female ALS with spinning field of more than 50 PI (Figs 75E, 77B), male with more than 30 PI (Fig. 78B); both sexes lack MAP on ALS. Female PMS with 2 anterior mAP spigots, a large posteromedian spigot (probably CY), and 8 smaller spigots (Fig. 77C); male PMS with 1 central mAP and 3 AC (Fig. 78C). Female PLS with anterobasal MS and 2 accompanying spigots and distal field of more than 35 AC (Figs 75F, 77D); male MS with 2 flanking nubbins, and only 3–4 AC (Figs 74B, 78D); male and female PLS with peculiar, large, clavate lateral setae. Male cribellar plate with no sign of spigots (Fig. 78E, F); numerous epiandrous gland spigots present (Fig. 74C, D).

#### Stegodyphus

Simon

http://species-id.net/wiki/Stegodyphus

Stegodyphus Simon 1873: 336; 1892: 253; Lehtinen 1967: 265. Type species *Eresus lineatus* Latreille, 1817.
Magunia Lehtinen, 1967: 246. Synonymy in Kraus and Kraus 1988.

##### Note.

With the transfer of *Stegodyphus annulipes* to the genus *Loureedia* gen. n., the genus *Stegodyphus* contains 20 recognized species from Africa, southern Europe and Asia, plus a single species from Brazil (Platnick 2011). The genus *Magunia* Lehtinen 1967 was established to accommodate *Stegodyphus tentoriicola* Purcell, 1904 and *Stegodyphus dumicola* Pocock, 1898, both of which have a bifurcated conductor including a hook-shaped sclerotized branch (Kraus and Kraus 1988: figs 245, 248). Kraus and Kraus (1988) rejected this genus on the grounds that it would require dividing *Stegodyphus* sensu lato into at least three genera, which they considered needless. Molecular analyses have corroborated the monophyly of *Stegodyphus* sensu lato and nested taxa representing *Magunia* within (not sister to) a monophyletic *Stegodyphus* (Johannesen et al. 2007; Miller et al. 2010a). We therefore concur with Kraus and Kraus (1988) in treating *Magunia* as a subjective junior synonym of *Stegodyphus*.

We studied specimens representing three *Stegodyphus* selected to cover a wide range of the variation present in the genus. Our exemplars were *Stegodyphus lineatus*, the type species for the genus, based on specimens from Afghanistan, Israel, and Turkey, *Stegodyphus mimosarum* based on specimens from Madagascar and Malawi, and *Stegodyphus sarasinorum* based on specimens from Myanmar. Our identification was based on the revision of Kraus and Kraus (1988); we did not seek to examine type material.

##### Diagnosis.

Distinguished from other eresids except *Paradonea presleyi* sp. n. by the median eye group, which has large, subequal eyes (AME/PME > 0.6) with clear separation on the horizontal axis and significant overlap on the vertical axis (Fig. 11A, C, E, G, I, K); distinguished from *Paradonea presleyi* sp. n. by the long, acute clypeal hood (short, slightly obtuse in *Paradonea presleyi* sp. n., Fig. 70I). Classically diagnosed by having the PER noticeably more narrow (65–90%) than the AER (Kraus and Kraus 1988; Simon 1892); however, *Adonea*, and some *Eresus* and *Paradonea* species approach or even overlap with some *Stegodyphus* species. Further distinguished from other eresids except *Dresserus*, *Gandanameno*, and some *Paradonea* by the advanced position of the PLE (< 0.29; > 0.31 in other eresids).

##### Distinguishing species.

Therevisionary monograph by Kraus and Kraus (1988) remains the key resource for identifying *Stegodyphus* species. Kraus and Kraus (1988) divided *Stegodyphus* into three species groups, each with one non-territorial permanently social species. In a molecular phylogenetic study, Johannesen et al. (2007) suggested that *Stegodyphus lineatus* is distinctive enough to warrant its own monotypic species group, but otherwise their findings were congruent with the earlier morphological study of Kraus and Kraus (1988) and supported the hypothesis that sociality in *Stegodyphus* has been independently derived multiple times. The *Stegodyphus africanus* group contains seven species including our exemplar, the social species *Stegodyphus mimosarum*. Males of the *Stegodyphus africanus* group have the palpal conductor as a distally-projecting complex comprising an outer leaf and a longer, less sclerotized, inner leaf. The embolic division is longer than the tegular division. The epigynum has the posterior part of median lobe raised, membranous, and wider than long. The median eyes in both sexes are relatively heterogenous (AME/PME ca. 0.6–0.8; data from Kraus and Kraus 1988). The *Stegodyphus dufouri* group contains four or five species (depending on whether *Stegodyphus pacificus* and *Stegodyphus dufouri* are considered distinct) including our exemplar, the social species *Stegodyphus sarasinorum*. Males of the *Stegodyphus dufouri* group have a spiral conductor that lacks a free inner leaf and is shorter than the tegular division. The median eyes in both sexes are relatively homogenous (AME/PME usually ca. 0.7–1.0, rarely ca. 0.6; data from Kraus and Kraus 1988). The *Stegodyphus mirandus* group contains six species including our exemplar, *Stegodyphus lineatus*, the type species of the genus (but see Johannesen et al. 2007). Males of the *Stegodyphus mirandus* group have complex conductors with a membranous inner lobe similar to that found in the *Stegodyphus africanus* group, although it is characteristically more spiral (as opposed to distally-projecting) and the embolic division is shorter than the tegular division. In some species (*Stegodyphus tentoriicola* and *Stegodyphus dumicola*), a sclerotized hook-like apophysis arises from the inner lobe of the conductor. The median eyes in both sexes are relatively homogenous (AME/PME ca. 0.8–0.9) except in *Stegodyphus tibialis* (AME/PME ca. 0.5–0.6; data from Kraus and Kraus 1988). The epigynum of some *Stegodyphus mirandus* group species is more or less rotated into vertical position (Kraus and Kraus 1988).

##### Natural history.

*Stegodyphus* are known from a variety of warm, dry habitats and include both solitary and social species. The solitary species make webs with a central, funnel-like retreat and an irregular cribellate mass appressed to the substrate or multiple cribellate sheets in different planes on which they walk. The social species make a large nest of silk, plant debris and chitinous remains of their insect prey, and large sheets of cribellate silk may extend out in several directions (Fig. 4J–L). The spiders walk upon or hang beneath these sheets. The cribellate silk carding leg is braced with a mobile leg IV, and the margins of the cribellate band are entire (Griswold et al. 2005). We have never observed them to wrap captured prey. The fine structure of the cribellate silk of *Stegodyphus* was studied by Kullmann (1975) who recorded both axial fibers and reserve warp and noted that the cribellar fibrils are cylindrical in cross section. Prey include a variety of arthropods (Dewar and Koopowitz 1970); social spiders hunting cooperatively are more effective than solitary hunters (Ward and Enders 1985). Juveniles feed on the bodies of dead females (Fig. 3D), which may or may not be their mother in social species (Schneider 2002). The sex ratio is female biased (more than 4:1) in social species (Avilés 1997). Social colonies last for 7–8 years. Mating in many species occurs in December–February (Kraus and Kraus 1988).

#### Stegodyphus lineatus

(Latreille)

http://species-id.net/wiki/Stegodyphus_lineatus

Figs 3A–C 4K 11A–D 15D–F 18G, J 79 [Fig F80] [Fig F81] [Fig F82] –83

Eresus lineatus Latreille, 1817: 393.
Eresus acanthophilus Dufour, 1820: 302, pl. 95, figs 3–4 (not seen); Walckenaer 1837: 399, pl. 11, fig. 1. Synonymy in Simon 1873: 337.
Eresus unifasciatus C. L. Koch, 1846: 5, fig. 1081. Synonymy in Simon 1873: 337.
Eresus fuscifrons C. L. Koch, 1846: 9, fig. 1084 (see Platnick 2011). Synonymy in Simon 1873: 337.
Eresus lituratus C. L. Koch, 1846: 11, fig. 1085. Synonymy in Simon 1873: 337.
Stegodyphus lineatus (Latreille, 1817). Simon 1873: 337; 1892: 253, fig. 208; 1910: 286, fig. 4A; Pavesi 1876: 443; Bacelar 1929: 252, figs 6–7; Nenilin and Pestova 1986: 1734, figs 5, 6, 8; Kraus and Kraus 1988: 231, figs 1–2, 28, 202–205, 227–228, 234–242; Ergashev 1990: 44, figs 17–23; Melic 1995: 11, figs 11–16; El-Hennawy 2009: 130, figs 3–4, 9, 11; Le Peru 2011: 321, fig. 562.
Eresus arenarius Kroneberg 1875: 44, pl. 5, fig. 32 (not seen). Synonymy in Nenilin and Pestova 1986: 1734.
Stegodyphus lineatus deserticola Simon 1908: 79; 1910: 287. Synonymy in Kraus and Kraus 1988: 232.
Stegodyphus quadriculatus Franganillo 1925: 38; 1926: 75. Synonymy in Kraus and Kraus 1988: 232.

##### Description.

**Figure 79.**
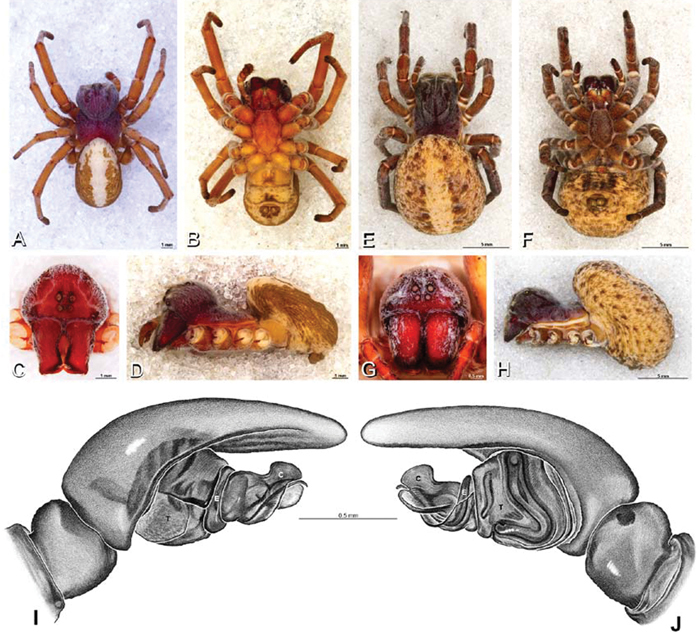
**A–J** *Stegodyphus lineatus*. **A–D, I, J** male from Nengrahar, Afghanistan (MR010, MR) **E–H** female from Belkis, near Birecor, Turkey (MR015, MR) **A–D** habitus of male, photomicrographs **E–H** habitus of female, photomicrographs **I, J** illustrations of left male palp **A, E** dorsal view **B, F** ventral view **C, G** anterior view **D, H** lateral view **I** prolateral view **J** retrolateral view. **C** conductor **E** embolus **T** tegulum.

*Male* (Nengrahar, Afghanistan, MR010, MR): Carapace with numerous white setae, cephalic region subtriangular, longer than wide, strongly raised; AME nearly as large as PME (AME/PME 0.88), median eyes separated on horizontal axis, largely overlapping on vertical axis; ALE on small tubercles; PER much narrower than AER (PER/AER 0.62), PLE position on carapace 0.24; clypeal hood forms acute angle; fovea shallow. Chelicerae with lateral boss, excavated mesally. Legs with numerous white setae; leg I distinctly thickened and elongated (contra Kraus and Kraus 1988: 232), without thick brush of setae; with distal ventral macrosetae on tibia I–IV, row of distal ventral macrosetae on metatarsus I–IV plus scattered ventral macrosetae on metatarsus and tarsus I–IV. Abdomen with numerous white setae (Figs 11A, B, 79A–D).

**Figure 80. F80:**
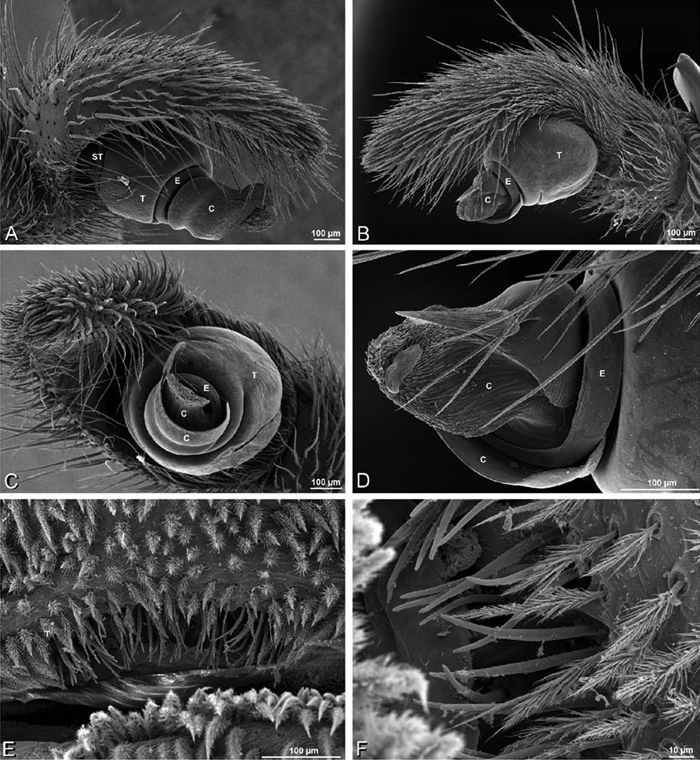
**A–D** male *Stegodyphus lineatus* from Nengrahar, Afghanistan (MR010, MR), scanning electron micrographs. **A–D** right palp, images reversed to appear as left palp **E, F** epiandrous region **A** prolateral view **B** retrolateral view **C** apical view **D** detail, prolateral view **E** ventral view **F** detail of epiandrous gland spigots. **C** conductor **E** embolus **ST** subtegulum **T** tegulum.

Male palp with proximal-distal axis; tegulum subtrapezoidal; conductor and embolus together form apical complex making one helical turn; conductor with more or less membranous and papilliated inner layer extending beyond moderately sclerotized outer layer; tegular division slightly longer than embolic division; cymbium with several prolateral macrosetae (Figs 15D–F, 79I, J, 80A–D).

**Figure 81. F81:**
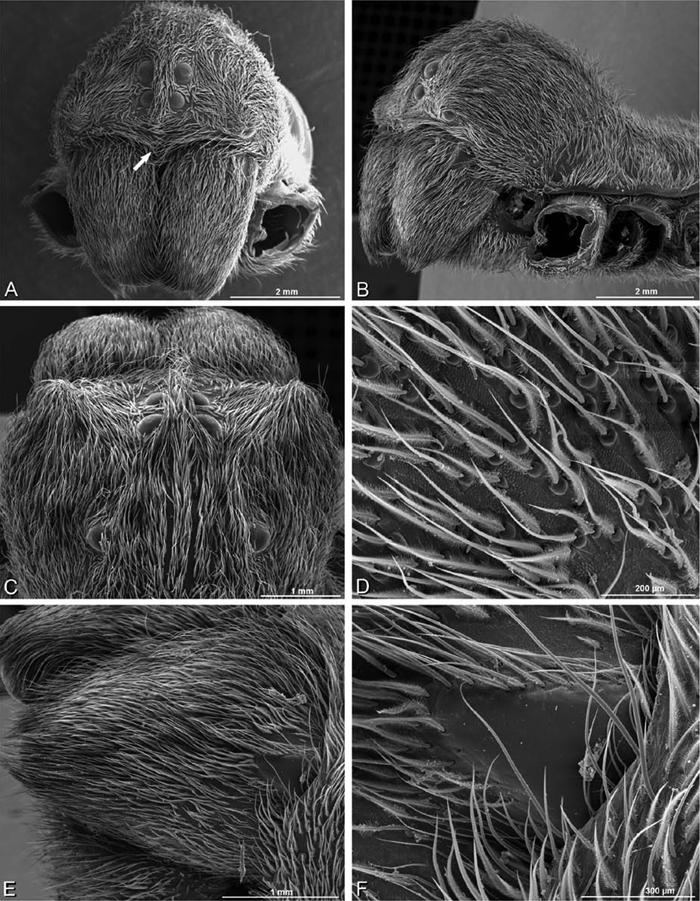
**A–F** Female *Stegodyphus lineatus* from Belkis, near Birecor, Turkey (MR015, MR), scanning electron micrographs of prosoma and chelicerae. **A** anterior view, arrow indicates clypeal hood **B** left lateral view **C** eye region, dorsal view **D** detail, prosoma cuticle **E** left chelicera **F** left cheliceral boss.

*Female* (Belkis, Turkey, MR015, MR): Carapace with numerous white setae, cephalic region subtriangular, longer than wide, moderately raised; AME nearly as large as PME (AME/PME 0.87), median eyes separated on horizontal axis, largely overlapping on vertical axis; ALE on small tubercles; PER much narrower than AER (PER/AER 0.68), PLE position on carapace 0.21; clypeal hood forms acute angle; fovea shallow. Chelicerae contiguous mesally, with lateral boss. Legs with numerous white setae, with pair of distal ventral macrosetae on tibia I–IV and row of distal ventral macrosetae on metatarsus I–IV plus scattered ventral macrosetae on metatarsus and tarsus I–IV. Abdomen with numerous white setae (Figs 11C, D, 79E–H, 81A–F).

**Figure 82. F82:**
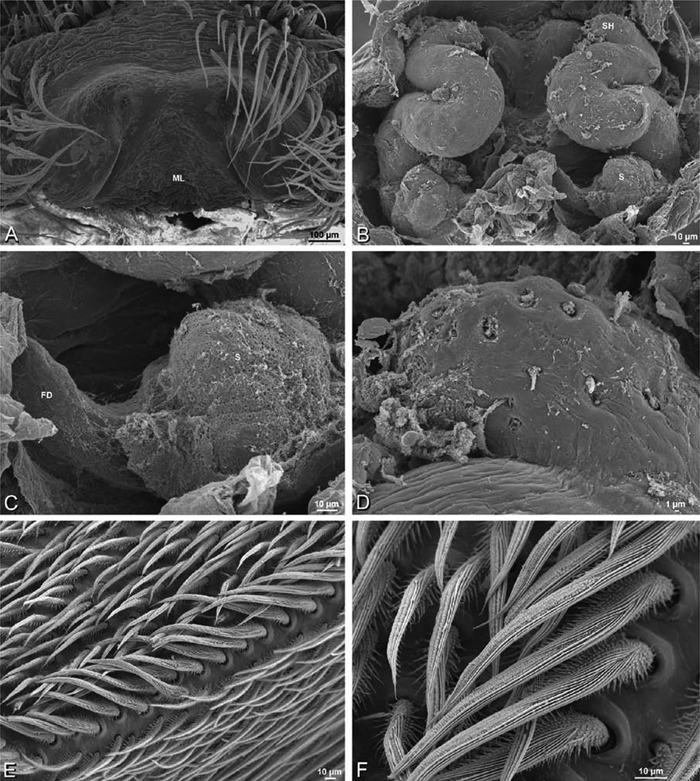
**A–F** female *Stegodyphus lineatus* from Belkis, near Birecor, Turkey (MR015, MR), scanning electron micrographs of epigynum, vulva, and calamistrum. **A** epigynum, ventral view **B** vulva, dorsal view **C** right spermatheca and fertilization duct **D** right spermathecal head **E** calamistrum, left metatarsus IV **F** detail, calamistrum setae. **FD** fertilization duct **ML** median lobe; **S** spermatheca **SH** spermathecal head.

Epigynum with converging slit-like atria occupying nearly the total length, anteriomedian part with weak notch-shaped invagination, anteriolateral margin a strong curved ridge (Figs 18G, 82A). Vulva with spermathecal heads on thick sinuous stalks leading to multilobed spermathecae posteriorly (Figs 18J, 82B–D).

**Figure 83.**
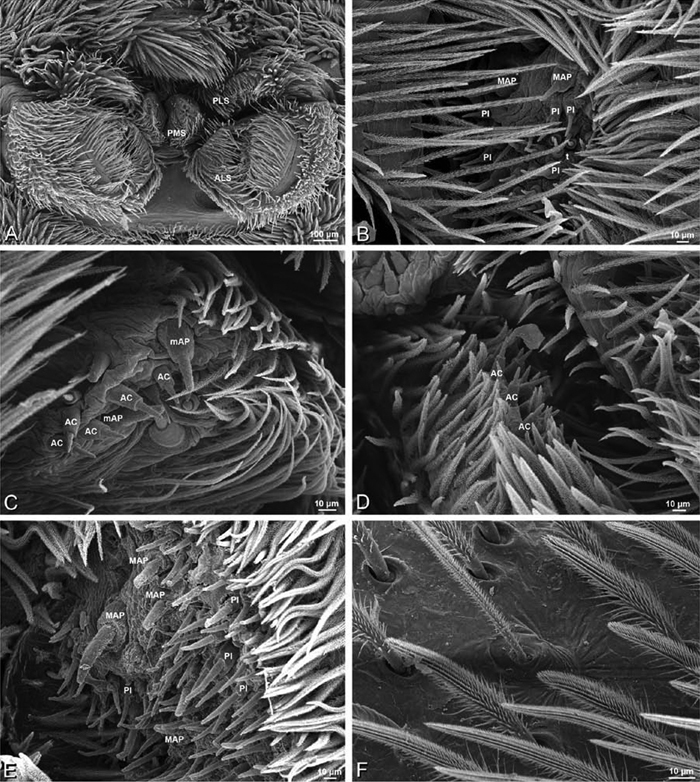
**A–F** *Stegodyphus lineatus*, scanning electron micrographs of spinnerets and trichobothrium. **A–D** male from Nengrahar, Afghanistan (MR010, MR) **E, F** female from Belkis,near Birecor, Turkey (MR015, MR). **A** overview **B, E** right ALS **C** left PMS **D** right PLS **F** trichobothrium on left metatarsus I. **AC** aciniform gland spigot **ALS** anterior lateral spinneret **MAP** major ampullate gland spigot **mAP** minor ampullate gland spigot **PI** piriform gland spigot **PLS** posterior lateral spinneret **PMS** posterior median spinneret.

##### Spinneret spigot morphology.

Our preparations are collapsed, obscuring the spigots of the posterior spinnerets, especially of the female. We are nevertheless able to make some provisional comments about the spigots. Female ALS with 5 MAP within and on inner edge of spinning field of more than 60 PI (Fig. 83E); male with at least 2 MAP within PI field; MAP shafts nearly smooth (Fig. 83B). Male PMS with 2 median mAP spigots flanked by a large anterior mAP tartipore (with shafts scaly as in other eresids), with 11 AC scattered anterior and posterior of mAP (Fig. 83C); female PMS spinning surface nearly hidden, but there appears to be numerous spigots, more than in male, so it is possible that, as in other eresids, both AC and CY are present. Male PLS with at least 13 AC (Fig. 83D); the base of the male PLS and most of the female PLS are not visible, so we cannot confirm the presence of a MS or flanking spigots. Male cribellar plate with no sign of spigots; numerous epiandrous gland spigots present (Fig. 80E, F).

#### Stegodyphus mimosarum

Pavesi

http://species-id.net/wiki/Stegodyphus_mimosarum

Figs 3E, F 4L 11E–H 15G–I 18H, K 84 [Fig F85] [Fig F86] [Fig F87] –88

Stegodyphus mimosarum Pavesi, 1883: 81; Simon 1909: 30; Strand 1913: 329; Lehtinen 1967: 461, fig. 454; Kraus and Kraus 1988: 195, figs 3–4, 8–12, 14–19, 37–39, 43–45, 49–51, 58–76, 96–99, 261, 266; 1990: 226, figs 6–8.
Stegodyphus gregarius O. Pickard-Cambridge 1889: 42, pl. 2, figs 4–5 (Synonymy in Kraus and Kraus 1988: 195).
Stegodyphus corallipes Simon 1906: 305 (Synonymy in Kraus and Kraus 1988: 195).
Stegodyphus hildebrandti (Karsch, 1878). Tullgren 1910: 95, pl. 1, fig. 5 (misidentified, see Kraus and Kraus 1988: 186).
Stegodyphus simoni Giltay, 1927: 105, figs 1–6 (Synonymy in Kraus and Kraus 1988: 195).

##### Description.

**Figure 84.**
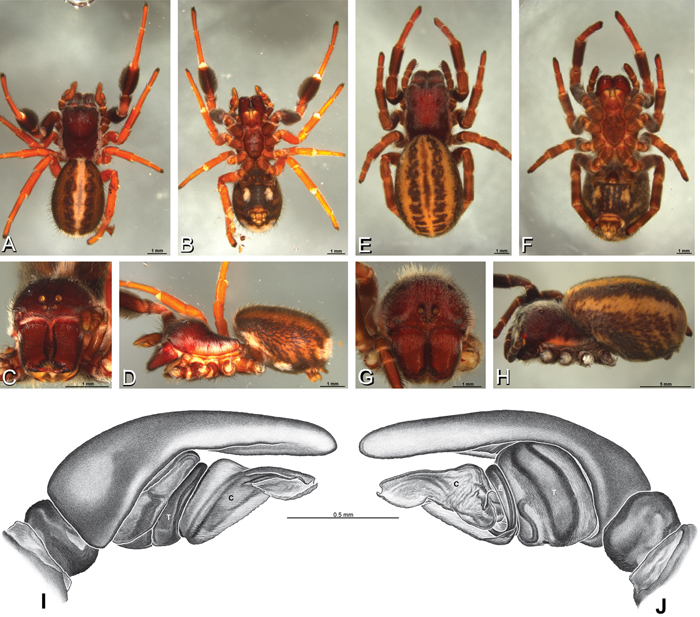
**A–J** *Stegodyphus mimosarum* from Forêt d'Analalava, Fianarantsoa, Madagascar (CASENT 9005869, CAS), images reversed. **A–D, I, J** male **E–H** female **A–D** habitus of male, photomicrographs **E–H** habitus of female, photomicrographs **J–K** illustrations of left male palp **A, E** dorsal view **B, F** ventral view **C, G** anterior view **D, H** lateral view **I** prolateral view **J** retrolateral view. **C** conductor **E** embolus **T** tegulum.

*Male* (Forêt d’Analalava, Madagascar, CASENT 9005869, CAS): Carapace with band of white setae around margin, longitudinal line in cephalic region and patches near PLE; cephalic region subtriangular, longer than wide, moderately raised; AME distinctly smaller than PME (AME/PME 0.62), median eyes separated on horizontal axis, largely overlapping on vertical axis; ALE on small tubercles; PER much narrower than AER (PER/AER 0.76), PLE position on carapace 0.32; clypeal hood forms acute angle; fovea shallow. Chelicerae with lateral boss, slightly excavated mesally. Legs with patches and longitudinal bands of white setae; leg I thickened with thick brush of dark setae on femur and especially tibia; with row of distal ventral macrosetae on metatarsus I–IV, a few scattered ventral macrosetae on tarsus I–IV and metatarsus II–IV. Dorsum of abdomen with median longitudinal stripe and posterior patch of white setae (Figs 11E, F, 84A–D).

**Figure 85. F85:**
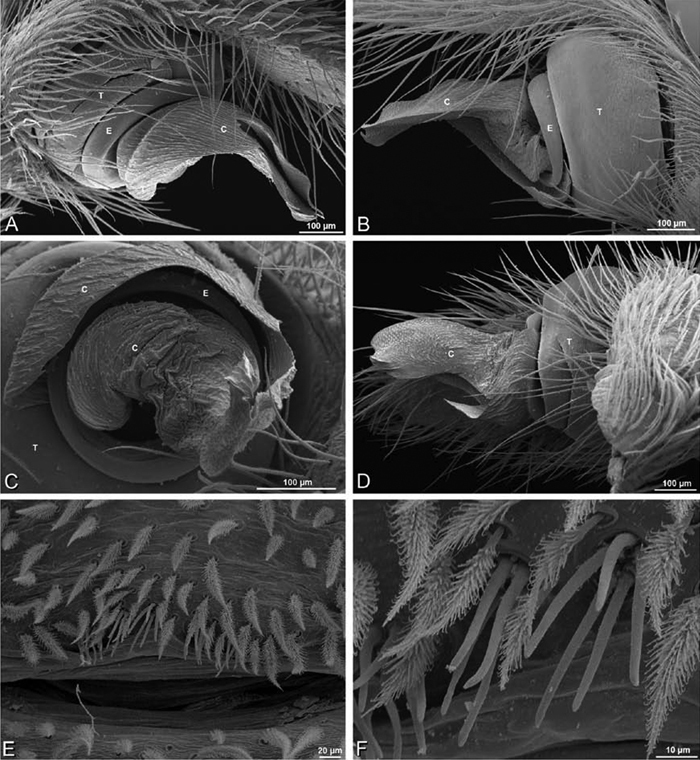
**A–F** *Stegodyphus mimosarum*, scanning electron micrographs of male from Forêt d'Analalava, Fianarantsoa, Madagascar (CASENT 9015950, CAS). **A–D** right palp, images reversed so as to appear a left palp **E, F** epiandrous gland spigots **A** prolateral view **B** retrolateral view **C** apical view **D** ventral view **E** epiandrous region, ventral view **F** detail, epiandrous gland spigots. **C** conductor **E** embolus **T** tegulum.

Male palp with proximal-distal axis; tegulum subtrapezoidal; conductor and embolus together form apical complex making one helical turn; conductor with more or less membranous and papilliated inner layer extending beyond moderately sclerotized outer layer; embolic division longer than tegular division; cymbium with several prolateral macrosetae (Figs 15G–I, 84I, J, 85A–D).

*Female* (Forêt d’Analalava, Madagascar, CASENT 9005869, CAS):Carapace with band of white setae around margin, densely mixed in cephalic region, fewer in thoracic region mesal to lateral band; cephalic region subtrapezoidal, longer than wide, moderately raised; AME distinctly smaller than PME (AME/PME 0.69), median eyes separated on horizontal axis, largely overlapping on vertical axis; ALE on small tubercles; PER much narrower than AER (0.77), PLE position on carapace 0.27; clypeal hood forms acute angle; fovea shallow. Chelicerae contiguous mesally, with lateral boss. Legs with patches of white setae; with row of distal ventral macrosetae on metatarsus I–IV, scattered ventral macrosetae on tarsus I–IV and metatarsus II–IV. Dorsum of abdomen with alternating light and dark longitudinal bands (Figs 11G, H, 84E–H).

**Figure 86. F86:**
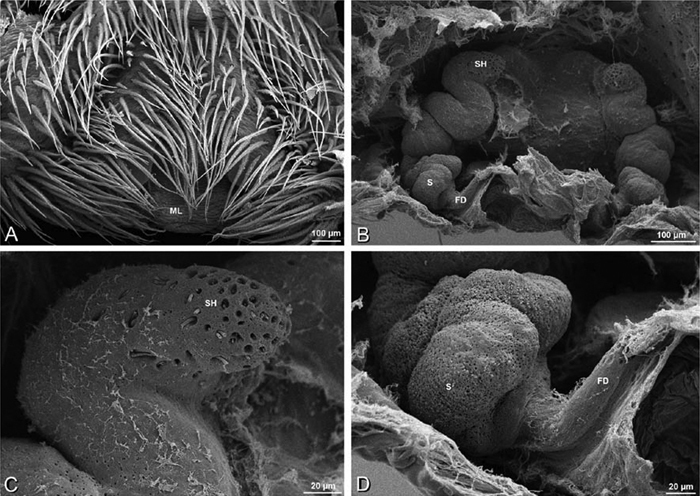
**A–D** *Stegodyphus mimosarum* female from Forêt d'Analalava, Fianarantsoa, Madagascar (CASENT 9015950, CAS), scanning electron micrographs of genitalia. **A** epigynum, ventral view **B** vulva, dorsal view **C** spermathecal head **D** spermatheca and fertilization duct. **FD** fertilization duct **ML** median lobe **S** spermatheca **SH** spermathecal head.

Epigynum bell-shaped with fleshy, bell-shaped median lobe, higher posteriorly than anteriorly, anteriomedian part with notch-shaped invagination (Figs 18H, 86A). Vulva with spermathecal heads on compact sinuous stalks leading to multilobed spermathecae posteriorly (Figs 18K, 86B–D).

**Figure 87. F87:**
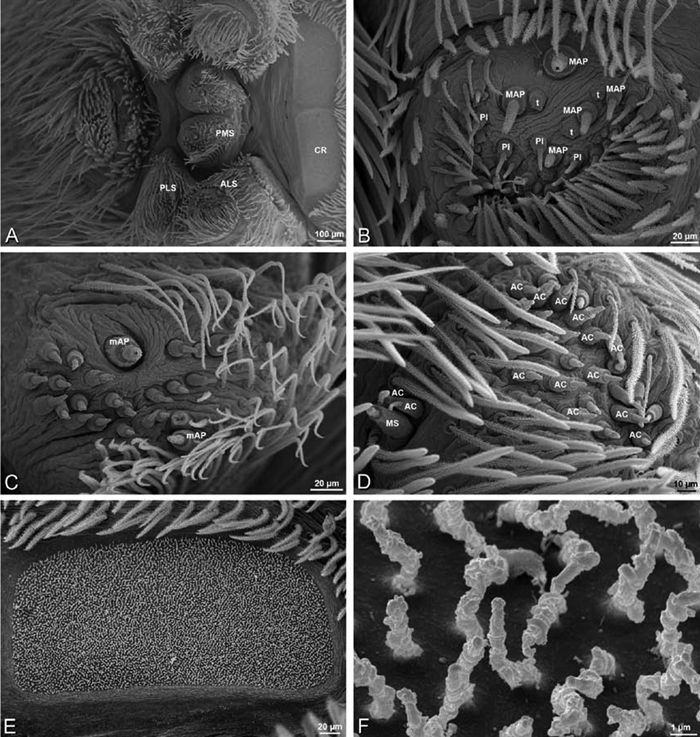
**A–F** *Stegodyphus mimosarum* female from Forêt d'Analalava, Fianarantsoa, Madagascar (CASENT 9015950, CAS), scanning electron micrographs of spinnerets. **A** Overview **B** Left ALS **C** Left PMS **D** Right PLS **E** Left lobe of cribellum **F** Cribellar spigots. Unlabeled spigots in **C** thought to be a mixture of aciniform gland spigots and cylindrical gland spigots. **AC** aciniform gland spigot **ALS** anterior lateral spinneret **CR** cribellum **MAP** major ampullate gland spigot **mAP** minor ampullate gland spigot **MS** modified spigot **PI** piriform gland spigot **PLS** posterior lateral spinneret **PMS** posterior median spinneret **t** tartipore.

**Figure 88.**
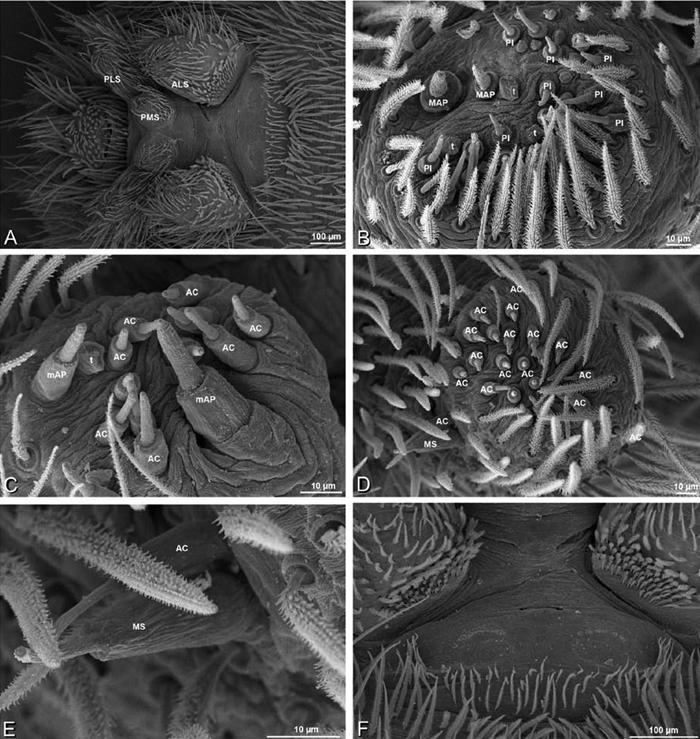
**A–F** *Stegodyphus mimosarum* male from Forêt d'Analalava, Fianarantsoa, Madagascar (CASENT 9015950, CAS), scanning electron micrographs of spinnerets. **A** Overview **B** Right ALS **C** Left PMS **D** Right PLS **E** Detail of modified spigot on right PLS **F** Vestigial cribellum. **AC** aciniform gland spigot **ALS** anterior lateral spinneret **CR** cribellum **MAP** major ampullate gland spigot mAP minor ampullate gland spigot **MS** modified spigot **PI** piriform gland spigot **PLS** posterior lateral spinneret **PMS** posterior median spinneret **t** tartipore.

##### Spinneret spigot morphology.

Female ALS with 6–8 MAP within and on inner edge of spinning field of 40–80 or more PI (Fig. 87B; Griswold et al. 2005: fig. 37B); male with 2 MAP and spinning field of more than 25 PI (Fig. 88B). Female PMS with 2 median mAP spigots, with posterior field of about 25 spigots of varying size and shape (Fig. 87C); male PMS with 1 median mAP and 12 AC (Fig. 88C); the large anterolateral spigot on the female may be a mAP or CY; other smaller spigots on the female may also be CY, though these cannot be differentiated morphologically (Fig. 87C). Female PLS with anterobasal MS with 2 accompanying AC spigots and distal field of 35–50 AC (Fig. 87D); male MS and flankers same, with about 18 AC (Fig. 88D, E). Male cribellar plate with no sign of spigots (Fig. 88F); numerous epiandrous gland spigots present (Fig. 85E, F). See also Griswold et al. (2005: 24–27, figs 34D–F, 25A–D, 36A–D, 37A–D).

#### Stegodyphus sarasinorum

Karsch

http://species-id.net/wiki/Stegodyphus_sarasinorum

Figs 11I–L 15I, L 18I, L 89 [Fig F90] [Fig F91] [Fig F92] [Fig F93] [Fig F94] –95

Stegodyphus sarasinorum Karsch, 1891: 275, pl. 10, fig. 4; 209, fig. 65; Bradoo 1975: 239, figs 9, 11; Tikader and Biswas 1981: 15, figs 5–7; Wunderlich 1986: 208, fig. 199; Kraus and Kraus 1988: 204, figs 21–27, 103, 110, 117, 120, 125, 139–141; Gajbe 2007: 428, figs 16–19.

##### Description.

**Figure 89.**
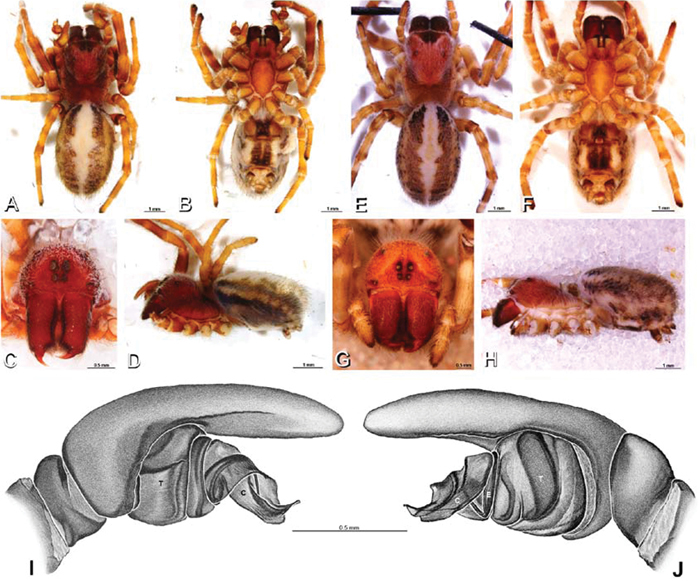
**A–J** *Stegodyphus sarasinorum* from 7.5 km E PwintPhyu, Magway Division, Myanmar (CASENT 9019370, CAS). **A–D,** **I–J** male, images reversed **E–H** female **A–D** habitus of male, photomicrographs **E–H** habitus of female, photomicrographs **I, J** illustrations of left male palp **A, E** dorsal view **B, F** ventral view **C, G** anterior view **D, H** lateral view **I** prolateral view **J** retrolateral view. **C** conductor **E** embolus **T** tegulum.

*Male* (7.5 km E PwintPhyu, Myanmar, CASENT 9019370, CAS): Carapace with few white setae, cephalic region subtriangular, longer than wide, moderately raised; AME nearly as large as PME (AME/PME 0.87), median eyes separated on horizontal axis, largely overlapping on vertical axis; ALE on small tubercles; PER much narrower than AER (PER/AER 0.86), PLE position on carapace 0.24; clypeal hood forms acute angle; fovea shallow. Chelicerae with lateral boss, slightly excavated mesally, with a single large keel-like tooth bearing denticles on the ectal side. Legs with numerous white setae; leg I somewhat thickened and elongated; with distal ventral macrosetae on tibia I–IV, row of distal ventral macrosetae on metatarsus I–IV plus scattered ventral macrosetae on metatarsus and tarsus I–IV. Abdomen with numerous white setae (Figs 11I, J, 89A–D).

**Figure 90. F90:**
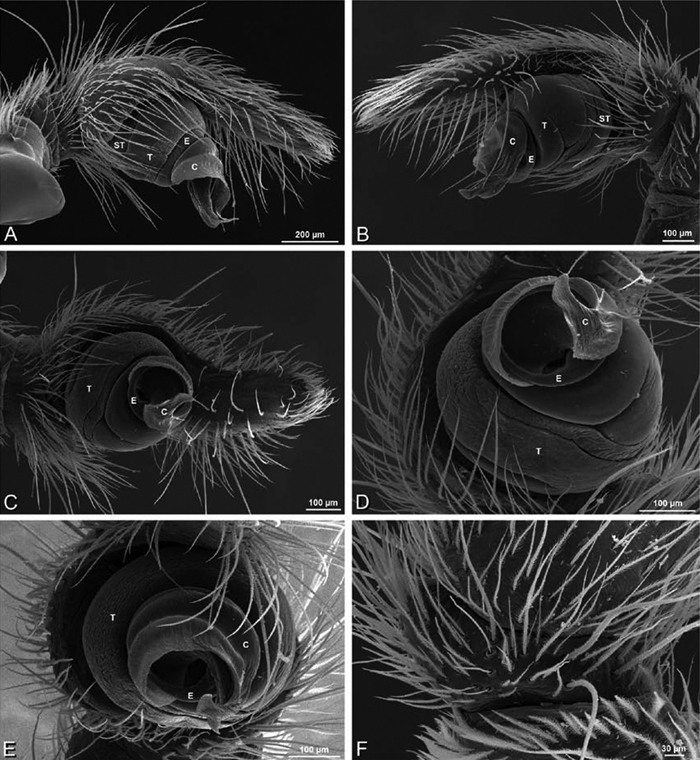
**A–F** *Stegodyphus sarasinorum* male from 7.5 km E PwintPhyu, Magway Division, Myanmar (CASENT 9019370, CAS), scanning electron micrographs of right palp, images reversed to appear as left palp. **A** prolateral view **B** retrolateral view **C** ventral view **D** ventral-apical view **E** apical view **F** palpal tibia, dorsal view. **C** conductor **E** embolus **ST** subtegulum **T** tegulum.

Male palp with proximal-distal axis; cymbium slightly excavated retrobasally; conductor and embolus together form apical complex making more than one helical turn; conductor moderately sclerotized, tegular division longer than embolic division; cymbium with several prolateral macrosetae (Figs 15J–L, 89I, J, 90A–E).

**Figure 91. F91:**
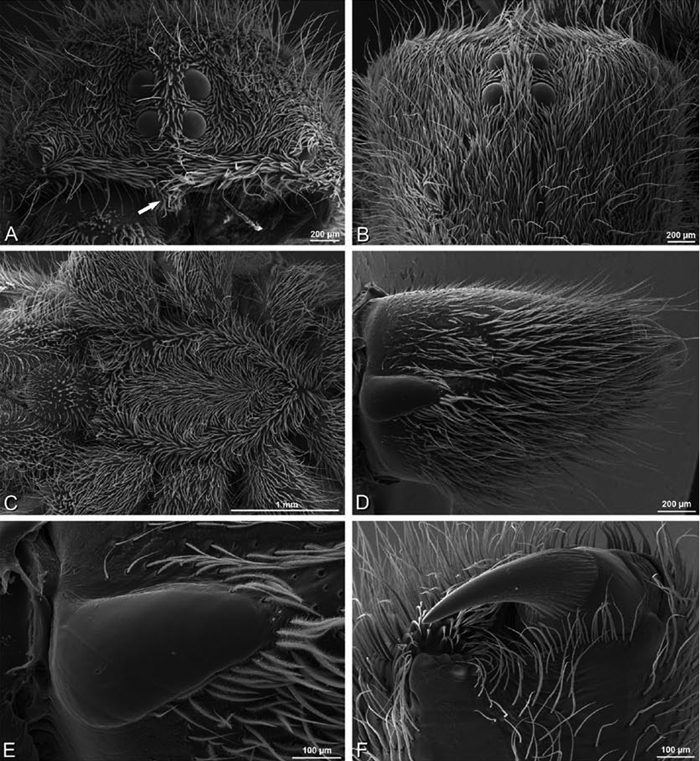
**A–F** *Stegodyphus sarasinorum* female from 7.5 km E PwintPhyu, Magway Division, Myanmar (CASENT 9019370, CAS), scanning electron micrographs of prosoma and chelicerae. **A** Prosoma, anterior view, left chelicera removed, arrow indicates clypeal hood **B** prosoma, dorsal view **C** sternum, ventral view **D** left chelicerae, ectal view **E** left cheliceral boss **F** distal part of left chelicera showing fang and teeth.

*Female* (7.5 km E PwintPhyu, Myanmar, CASENT 9019370, CAS):Carapace with numerous white setae, cephalic region subtriangular, longer than wide, slightly raised; AME nearly as large as PME (AME/PME 0.84), median eyes separated on horizontal axis, largely overlapping on vertical axis; ALE on small tubercles; PER much narrower than AER (0.86), PLE position on carapace 0.24; clypeal hood forms acute angle; fovea shallow. Chelicerae contiguous mesally, with lateral boss. Legs with numerous white setae; with pair of distal ventral macrosetae on metatarsus I–IV, scattered ventral macrosetae on tibia III and metatarsus and tarsus III–IV. Abdomen with numerous white setae (Figs 11K, L, 89E–H, 91A, B, D, E).

Epigynum with slit-like atria that converge then briefly diverge near anterior limit, occupying nearly the total length, anteriomedian part with moderate notch-shaped invagination, anteriolateral margin a moderate curved ridge (Figs 18I, 93A). Vulva with spermathecal heads on long sinuous stalks leading to multilobed spermathecae posteriorly (Figs 18L, 93B–E).

**Figure 92. F92:**
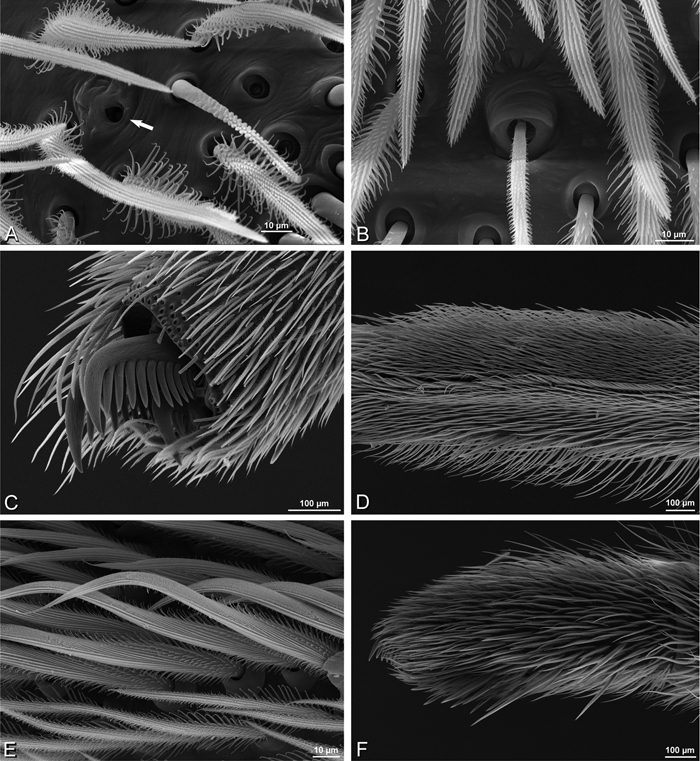
**A–F**, *Stegodyphus sarasinorum*, scanning electron micrographs of female from 7.5 km E PwintPhyu, Magway Division, Myanmar (CASENT 9019370, CAS). **A** tarsal organ indicated by arrow, left leg I **B** trichobothrium, left metatarsus I **C** tarsal claws, left legI retrolateral view **D** calamistrum, left metatarsus IV **E** detail of calamistrum setae **F** left female palp, retrolateral view.

**Figure 93. F93:**
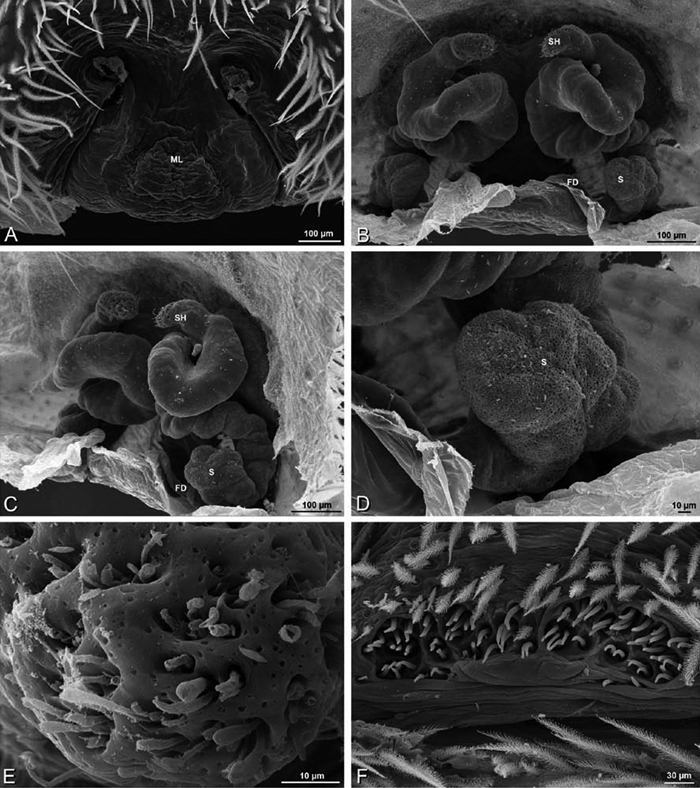
**A–F**, *Stegodyphus sarasinorum* from 7.5 km E PwintPhyu, Magway Division, Myanmar (CASENT 9019370, CAS), scanning electron micrographs. **A–E** female **F** male **A** epigynum, ventral view **B** vulva, dorsal view **C** vulva, dorsolateral view **D** detail of spermatheca **E** detail of spermathecal head **F** epiandrous gland spigots. **FD** fertilization duct **ML** median lobe **S** spermatheca **SH** spermathecal head.

**Figure 94. F94:**
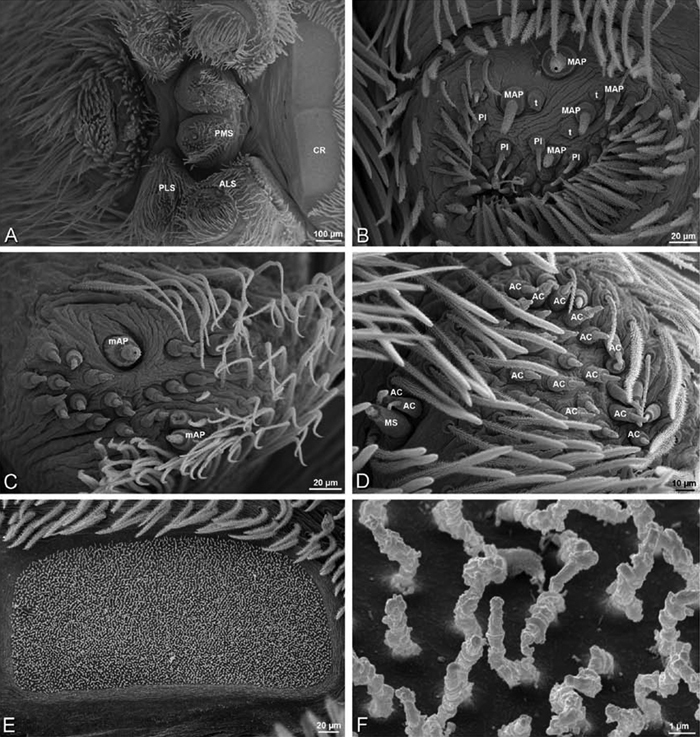
**A–F** *Stegodyphus sarasinorum*, scanning electron micrographs of spinnerets of female from 7.5 km E PwintPhyu, Magway Division, Myanmar (CASENT 9019370, CAS). **A** overview **B** right ALS **C** left PMS **D** right PLS **E** cribellum **F** cribellar spigots. Unlabeled spigots in **C** thought to be a mixture of aciniform gland spigots and cylindrical gland spigots. **AC** aciniform gland spigot **ALS** anterior lateral spinneret **CR** cribellum **MAP** major ampullate gland spigot **mAP** minor ampullate gland spigot **MS** modified spigot **n** nubbin **PI** piriform gland spigot **PLS** posterior lateral spinneret **PMS** posterior median spinneret **t** tartipore.

**Figure 95.**
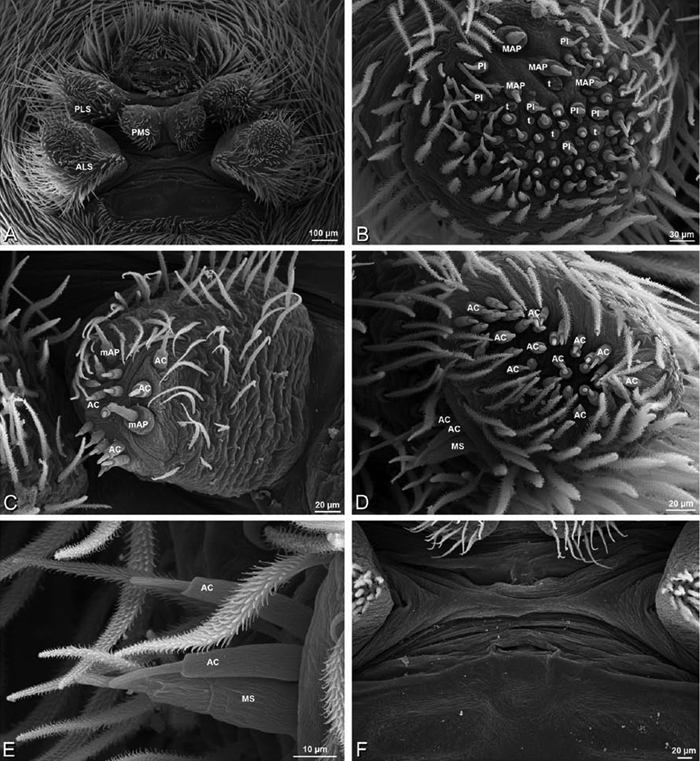
**A–F** *Stegodyphus sarasinorum*, spinnerets of male from 7.5 km E PwintPhyu, Magway Division, Myanmar (CASENT 9019370, CAS). **A** overview **B** right ALS **C** right PMS **D** right PLS **E** detail of modified spigot on right PLS **F** vestigial cribellum. **AC** aciniform gland spigot **ALS** anterior lateral spinneret **MAP** major ampullate gland spigot **mAP** minor ampullate gland spigot **MS** modified spigot **PI** piriform gland spigot **PLS** posterior lateral spinneret **PMS** posterior median spinneret **t** tartipore.

##### Spinneret spigot morphology.

Female ALS with at least 7 MAP within and on inner edge of spinning field of more the 90 PI and many small tartipores (Fig. 94B); male with at least 1 MAP on inner margin and 3 more within spinning field of about 50 PI (Fig. 95B). Female PMS with 1 central mAP spigot, a large anterior spigot (probably CY) flanked by 2 large tartipores, and 30 smaller spigots scattered from anterior to posterior (Fig. 94C); male PMS with 1 central mAP and about 9 AC, suggesting that the additional spigots on the female may comprise AC and CY spigots (Fig. 95C). Female PLS with anterobasal MS and 1 accompanying spigot and distal field of more than 35 AC (Fig. 94D); male MS with 2 flanking spigots, also with about 35 AC (Fig. 95D, E). Male cribellar plate with no sign of spigots (Fig. 95F); numerous epiandrous gland spigots present (Fig. 93F).

## Supplementary Material

XML Treatment for Eresidae

XML Treatment for Adonea

XML Treatment for Adonea fimbriata

XML Treatment for Dorceus

XML Treatment for Dorceus fastuosus

XML Treatment for Dresserus

XML Treatment for Dresserus

XML Treatment for Eresus

XML Treatment for Eresus walckenaeri

XML Treatment for Eresus kollari

XML Treatment for Gandanameno

XML Treatment for Gandanameno

XML Treatment for Loureedia

XML Treatment for Loureedia annulipes

XML Treatment for Paradonea

XML Treatment for Paradonea striatipes

XML Treatment for Paradonea splendens

XML Treatment for Paradonea variegata

XML Treatment for Paradonea parva

XML Treatment for Paradonea presleyi

XML Treatment for Seothyra

XML Treatment for Seothyra henscheli

XML Treatment for Stegodyphus

XML Treatment for Stegodyphus lineatus

XML Treatment for Stegodyphus mimosarum

XML Treatment for Stegodyphus sarasinorum

## Appendix 1

Specimen data.

All specimens examined unless otherwise indicated.

*Adonea fimbriata* Simon, 1873

Primary types

**Algeria** and **Tunisia**: ♂♂ (uncounted) [no date] (735, AR5439, MNHN, syntypes).

Additional specimens examined

**Algeria-Morocco:** 3 ♂ [no date] (MR012, MR). **Israel:** 1 ♀, Merhav Am village [30.88802°N, 34.82817°E], September 2007 (J. Kral, MR003, MR); 1 ♀, Wadi Mashash, 20 km S of Beer Sheva, 22 July 1993 (MR013, HUJ).

*Dorceus fastuosus* C. L. Koch, 1846

Primary types

*Dorceus fastuosus* C. L. Koch, 1846

**Senegal:** 1 ♂, Mian (Kat.-Nr. 1527, ZMHB, not examined). Note: As reported by El-Hennawy 2002, the holotype is a dry pinned specimen.

*Dorceus viberti* Simon, 1910

**Tunisia:** 1 ♂ [+ 1 misidentified ♀, see El-Hennawy 2002: 62] Nefzana [Nefza?], Vibert [no date] (9126, AR5404, NMHN, holotype).

Additional specimens examined

**Israel:** 5 ♂, Mashabin Sand Dunes, 23 August 2007 (J. Kral, MR006, HUJ); 1 ♀, Mashabim sand dunes, collected as juvenile 18 January 2009, matured in captivity 1 May 2009 (I. Hoffman, MR002, MR); 1 ♀, Negev, Mashabim sand dunes, 2008 (J. Kral, MR); 1 ♀, Negev desert, Mashabim Reserve, top of the sandy hill between the crop station of the Ben Gurion University and Wadi Bessor, Vysata [“sucked" - mother cannibalized by spiderlings], nest 1, 2008 (J. Kral, MR). **Senegal:** 3 ♂, Dakar [no date] (1237, AR 5405, NMNH).

*Dresserus* spp.

Specimens examined

**Republic of South Africa:** *KwaZulu-Natal Province*: 1 ♀ Drummond, 600 m, 29°44'S, 30°42'E, 16 December 1990 (V. D. & B. Roth, CASENT 9037024, CAS); 1 ♂, Hluhluwe Game Reserve, 18 November 1992 (Endrody-Younga, TM 19739, TMSA); *Mpumalanga*: 1 ♂, Witbank, 10 November 1991 (TM 19738, TMSA); *Northern [Limpopo] Province*: Klein Kariba, ca 7 km W Warmbad, 24°50'S, 28°20'E, 24–28 November 1996, 1140 m (C. E. Griswold, CASENT 9025745, CAS). **Tanzania:** 1 ♂, Tanga region, Muheza District, Manga Forest Reserve, 38°47'E, 5°02'S, October–December 1994 (Frontier Tanzania, ZMUC); 1 ♀, Tanga region, West Usambara Mts., Mazumbai station [Mazumbai Research Station], 4°48.5'S, 38°30'E, 1500m, 10–20 November 1995 (C. E. Griswold, N. Scharff, and D. Ubick, CASENT 9025747, CAS); 1 ♂, Tanga region, West Usambara Mts., Mazumbai station [Mazumbai Research Station], 4°48.5'S, 38°30'E, 1500m, 10–20 November 1995 (C. E. Griswold, N. Scharff, and D. Ubick, CASENT 9025746, CAS).

*Eresus walckenaeri* Brullé, 1832

Primary type

**Greece:** 4 ♀, Laconie near Sparta [37.075°N, 22.43333°E] (syntypes, presumed lost, not examined).

Specimens examined

**Bulgaria:** 2 ♂, Kresna, Skalni Stepi, 27 May 2008 (J. Chytil, MR). **Greece:** *Pieria*: 1 ♂, 1 ♀, Leptokaryas, 40°3.221'N, 22°32.917'E, 22 June 2008 (J. Maruch, MR020, MR); *Lakonia*: 1♀, 5 km south of Monemvasia, 6–20 April 1981 (Bjarne Skule leg., ZMUC 00012903, ZMUC).

*Eresus kollari* Rossi, 1846

Primary type

**Austria:** 1 ♀, Baden near Vienna [48.20833°N, 16.37166°], August 1841 (K. Kollar, NMW, syntype).

Additional specimens examined

**Czechia:** 18 ♀, Srbsko, res. Koda [49°56'0.896"N, 14°7'13.176"E] 29 October 2002, rocky steppe on limestone (M. Řezáč, MR016, MR); 6♂, Prague, res. Radotinske udoli [49°59'59.41"N, 14°19'6.579"E] 1998, rocky steppe on limestone (M. Řezáč, MR007, MR). **Hungary:** 1 ♂,Remete Mountain, close to Remeteszolos [Remeteszőlős], ~8 km NNW from Budapest, 18.93051°E, 47.56685°N, 3 October 2007 (G. Kovacs [Kovács], CASENT 9037134, CAS). See Řezáč et al. (2008) for additional specimen records.

*Eresus sandaliatus* (Martini & Goeze, 1778)

Specimens examined

**Denmark:** 1 ♂, 1 ♀ SE of Silkeborg, on road from Rye to Gl. Salten, 9°35'E, 56°05'N, 25 November 1994 (P. d. Place Bjørn, CASENT 9039243, CAS).

*Gandanameno* Lehtinen, 1967

Primary types

*Eresus depressus* Tucker, 1920

**South Africa:** *Eastern Cape*: 6 ♀, Hanover, E. Karoo [31.068°S, 24.440°E] September–October 1901, (S. C. Schreiner, SAM-ENW-X012852, SAM, syntypes); 5 ♀, Hanover, E. Karoo [31.068°S, 24.440°E] September–October 1901 (S. C. Schreiner, SAM-12852, SAM, syntypes).

*Eresus echinatus* Purcell, 1908

**Namibia:** 1 ♀, Rooibank, SW of Walvis Bay, S. Hereroland [23.174°S, 14.655°E] May 1905 (L. Schultze, SAM-ENW-X150519, SAM, holotype).

*Eresus purcelli* Tucker, 1920

**South Africa:** *Eastern Cape*: 1 ♀, East London [33.013°S, 27.903°E] 29 September–October 1905 (Mr. & Mrs. W. F. Purcell, SAM-ENW-B002435, SAM, holotype).

*Eresus spenceri* Pocock, 1900

**South Africa:** *Eastern Cape*: 1 ♀, Port Elizabeth [33°58'S, 25°36'E] 18 July 1890 (BMNH, holotype).

*Eresus inornatus* Pocock, 1898

**Nyasaland [Malawi]:** 1 ♀, Nyika Plateau [10.350°S, 33.600°E] 25 April 1897 (A. Whyte, BMNH, holotype).

*Eresus bubo* L. Koch, 1865

**South Africa:** *Eastern Cape*: ♀, Algoa Bay [33°50'S, 25°50'E] (not examined).

*Eresus namaquensis* Purcell, 1908

**South Africa:** *Northern Cape*: ♀, Namaqualand, Steinkopf [29°16'S, 17°44'E] July–August 1904 (L Schultze, not examined).

*Gandanameno* sp.

Additional specimens examined

**Namibia:** 1 ♀, 20 juveniles, Gobabeb, Kuiseb River Bed [23.6°S, 15°E], 7 February 1978, with an egg sac, O. Lomholdt (19780207, ZMUC); 1 ♀, Homeb, 23.638350°S, 15.18663333°E, October 2009, under the bark on the tree (M. Forman, F. Stahlavsky, MF collection #9, “adult 13 October 2009, died 29 January 2010, I breed few of their offspring", DNA sample RMNH.ARA.1452113, RMNH). **Republic of South Africa:** *Eastern Cape*: 1 ♂, 7 Park Road, Grahamstown, C.P. [33.3°S, 26.528°E] 25 March 1979, under dry brick in aviary (P. Hawkes, P. Croeser, AcAT 82/317, NCA); 1 ♀, Addo Bush, C. Col. [33°32'S, 25°50'E, SAM database] September 1919 (J. Drury, SAM-B4653, SAM); 1 ♀, Cradock [33.878°S, 18.574°E] 25 October 1989, pit trap (M. de Jager, AcAT 90/464, NCA); 1 ♂, Farm Hermanuskraal near Fort Brown [33.143°S, 26.621°E] 2 October 1993, on soil-pit trap (M. Burger, AcAT 96/34, M.B.-236, NCA); 1 ♂, 2 juveniles, Graaff-Reinet [32°15'S, 24°33'E, SAM database] September 1902 (J. Paynter, SAM 12571, SAM); 1 ♀, Grahamstown [33.310°S, 26.520°E] October 1979 (P. Croeser, AcAT 82/18, NCA); 4 ♀, Grahamstown [33.310°S, 26.520°E] 15 October 1938 (Tenther, 19381015, MHNG); 1 ♀, Grahamstown [33.310°S, 26.520°E] (Reimoser, MHNG); 1 ♀, Grahmstown, Gretna Farm [33.310°S, 26.520°E] 1 January 1980, in web in tree stump (P. Hawkes, AcAT 82/108, NCA); 1 ♀, Middleburg, 31°33.714'S, 24°48.229'E, 7 February 2006 (J. Miller, H. Wood, L. Lotz, M. de Jager, DNA sample 09-05, CASENT 9023623, CAS); 1 ♀, Port Elizabeth [33.958°S, 25.619°E] 5 April 1987, bark of tree in caravan park (G. Gelderblom, AcAT 89/40, NCA); 1 ♀, Port Elizabeth [33.9°S, 25.6°E] (S. Hansen, port-3325, ZMHB); 1 ♀, Port Elizabeth [33°58'S, 25°36'E, SAM database] 29 July 1899 (J. L. DrŠje, SAM-5398, SAM); 1 ♀, Port Elizabeth [33°58'S, 25°36'E, SAM database] November 1897 (J. L. DrŠje, SAM-2148, SAM); 1 ♀, Port Elizabeth [33°58'S, 25°36'E, SAM database] January 1900 (J. L. DrŠje, SAM-8461, SAM); 1 ♀, Tarkastad [32°1'S, 26°18'E, SAM database] 1903, (R. Brown [R. Pegler in SAM database], SAM-12887, SAM); 1 ♀, 1 juvenile, Uitenhage district, Dunbrody [33°28'S, 25°33'E, SAM database] 1903 (J. A. O'N,eil, SAM-12881, SAM); 1 ♀, Uitenhage district, Dunbrody [33°28'S, 25°33'E, SAM database] 1901 (J. A. O'N,eil, SAM-9215, SAM); 1 ♂, Uitenhage, Springs Resort, 33°42'S, 25°26'E, 19 January 2004, Night hunting (L. Lotz, NMBA 09196, BMSA, DNA sample 20-05); 1 ♀, 1 juvenile, Willowmore [33°18'S, 23°29'E, SAM database] 1903 (H. Brauns, SAM-12846, SAM); 1 ♀, Willowmore, Uitspan, 33°31'S, 23°42'E, 27–28 February 2008, in cave (L. Lotz, NMBA 11579, BMSA, DNA sample 19-06); *Free State Province*: Amanzi Game Reserve 11.64 km 344° N Brandfort, 28°35.438'S, 26°26.145'E, 1425 m, 25 October 2011, bushveld/ grassland (L. Almeida, C. Griswold, T. Meikle, C. Haddad, CAS); 1 ♀, Bloemfontein, 29°8'S, 26°10'E, 8 September 1991, dead on dogs nose (A. Truter, NMBA 08050, BMSA); 1 ♀, Bloemfontein, 29°8'S, 26°10'E, 13 January 1994 (A. Lombard, NMBA 07917, BMSA); 1 ♀, Bloemfontein, 29°8'S, 26°10'E, 9 May 1993 (A. Pretorius, NMBA 07950, BMSA); 1 ♀, Bloemfontein, 29°8'S, 26°10'E, 17 September 1989, in house (S. Louw, NMBA 02940, BMSA); 1 ♀, Bloemfontein, 29°8'S, 26°10'E, 25 February 1991, under stone (Museum Staff, NMBA 05379, BMSA); 1 ♂, Bloemfontein, 29°8'S, 26°10'E, 26 September 1990, in house (J. du Preez, NMBA 05322, BMSA); 1 ♀, Bloemfontein, 29°8'S, 26°10'E, 27 January 1997, in garden (L. Lotz, NMBA 07948, BMSA, DNA sample 19-03); 1 ♀, Bloemfontein, 29°8'S, 26°10'E, 19 January 1998, in garden (B. v/d Walt, NMBA 08705, BMSA); 2 ♀, Bloemfontein, 29°8'S, 26°10'E, 12 November 1998, in house (R. Nuttall, DNA sample 19-07, NMBA 08847, BMSA); 1 ♀, Bloemfontein [29°S, 26.100°E] 29 January 2005 (from Johannesen et al. 2007, M. Řezáč, 14-06); 1 ♀, Brandfort, 28°42'S, 26°27'E, 1974, by hand (S. van Ee, NMBA 05250, BMSA); 1 ♀, Jagersfontein, Klein Preezfontein, 29°49'S, 25°25'E, 15 August 1989, under stones (Students, NMBA 02925, BMSA); 1 ♀, Jacobsdal, Kimberley Rd., 29°8'S, 24°41'E, 4 May 1988, in channel (Museum Staff, NMBA 02568, BMSA); 1 ♂, nr Lindley, Doornkloof Nature Reserve, Overspruit Campsite [30.331°S, 24.987°E] 25 September 2007, in web under rocks (D. du Plessis, AcAT 2008/554, NCA); 1 ♀, Vaalpark [26.775°S, 27.849°E] 16 November 1993, in house, strong silk thread (E. Els, AcAT 94/64, NCA); *[Gauteng?]*: 1 ♀, Transvaal [26°S, 28°E] (Reimoser, MHNG); *Gauteng*: 1 ♀, Botha Plotte [26.200°S, 27.670°E] 8 April 1987, on tree trunk (C. Scheepers, AcAT 89/75, NCA); 1 ♀, Craighall, Johannesburg [26.115°S, 28.026°E] 11 May 1987, from tree in garden (Rod Wilson, AcAT 87/731, NCA); 1 ♀, Johannesburg [26°12'S, 28°5'E, SAM database] (SAM-12824, SAM); 1 ♀, Johannesburg, Melville Koppies [26.169°S, 28°E] 7 April 1988, web under rock (L. Prendini, AcAT 91/911, NCA); 1 ♀, Magalieoberg [Magaliesberg] district [26°S, 27°32'45"E] 13 March 1987, ander bas v boon (M. Vogt, AcAT 88/190, NCA); 1 ♀, Pretoria [25.742°S, 28.188°E] 5 April 1987, Op klipmuur in gat (H. Scheepers, AcAT 91/1104, NCA); 1 ♂, Pretoria, 25.450°S, 28.120°E, 15 October 1986 (L. Vrey, AcAT 86/578, NCA); 1 ♀, Pta [Pretoria], Hatfield, [25.750°S, 28.230°E] 14 February 1987, on tree (L. van Heerden, AcAT 90/322, NCA); 1 ♀, Pretoria, Leaufontein, Roodeplaat Dam [25°37'44.15"S, 28°21'2.08"E] 3 December 2002 (L. Prinsloo, TM21768, TMSA); 1 ♀, Rietfontein, Pretoria [25.707°S, 28.221°E] 13 November 1997, in garden (B. Sunkel, AcAT 98/48, NCA, DNA sample 20-04); 1 ♀, Roodepoort, Kloofendal [26.133841°S, 27.885662°E] 16 February 1989, under loose bark of gum trees (A. Leroy, AcAT 89/688, LR493, NCA); *KwaZulu-Natal*:1 ♀, Greytown [29.064°S, 30.389°E] 25 June 1983 (P. Reavell, AcAT 92/408, NCA); 1 ♀, Ophathe Game Reserve, Ophathe River crossing, 28°23.727'S, 31°23.643'E, 5 July 2007, active searching under logs (C. Haddad, DNA sample 13-10, NCA); 1 ♀, Ophathe Game Reserve, Ophathe River crossing, 28°23.727'S, 31°23.643'E, 5 July 2007, active searching under logs (C. Haddad, DNA sample 19-02, NCA); 1 ♀, 1 juvenile, Vernon Crookes Nature Reserve Nr. Park Rynie [30.300°S, 30.720°E] 25 March 1985 (R. Maartens, AcAT 86/565, NCA); *Northern Cape*: 1 ♂, Barkly West, Vaalbos National Park [28°26'S, 24°16'E] 17–21 October 1988, under stones (Ent. Staff, NMBA 02789, BMSA); 2 ♀, E Karroo, Hanover [31°4'S, 24°27'E, SAM database] December 1901 (S. C. Schreiner, SAM-12854, SAM); 1 ♀, E Karroo, Hanover [31°4'S, 24°27'E, SAM database] November 1901 (S. C. Schreiner, SAM-12853, SAM); 1 ♀, E Karroo, Hanover [31°4'S, 24°27'E, SAM database] January 1902 (S. C. Schreiner, SAM-11800, SAM); 4 ♂, E. Karroo, Hanover [31°4'S, 24°27'E, SAM database] September–October 1901 (S. C. Schreiner, SAM 9465, SAM); 3 ♀, Eierfontein, 8–9 miles west of Hanover [31°5'S, 24°18'E, SAM database] December 1901–February 1902 (S. C. Schreiner, SAM-12823, SAM); 1 ♀, Eierfontein, 8–9 miles west of Hanover [31°5'S, 24°18'E, SAM database] December 1901–February 1902 (S. C. Schreiner, SAM-11948, SAM); 1 ♀, Eierfontein, 8–9 miles west of Hanover [31°5'S, 24°18'E, SAM database] 1902 (S. C. Schreiner, SAM-12579, SAM); 1 ♀, Hanover [31.068°S, 24.440°E] (SAM-ENW-B006896/SAM-9958, SAM); 1 ♀, Hanover, E Karroo, C.P. [31°4'S, 24°27'E, SAM database] December 1901 (S. C. Schreiner, SAM-11813, SAM); 1 ♀, Kimberley [28.743°S, 24.762°E] December 1918 (J. H. Power, SAM-B4202/Aran 2248, SAM); 1 ♀, Kleinsee [29°40'S, 17°5'E, SAM database] March 1935 (A. J. Hesse, Thorne, R. F. Lawrence, SAM-B8885, SAM); 1 ♀, Namaqualand [29.350°S, 17.630°E] 1905 (G. Alston, SAM-14426/Aran 2246, SAM); 2 ♀, Namaqualand, Steinkopf [29°16'S, 17°44'E, SAM database] July–August 1904 (L. Schultze, SAM-150518/Aran 2165, SAM); 1 ♀, Namaqualand, Wolfberg, 29°37'S, 17°25'E, 16 September 1994 (J. Irish, NMBA 08112, BMSA); 1 ♂, Poortjiesfontein, 5–6 miles N of Hanover [31°S, 24°28'E, SAM database] 1905 (E. Neeser, SAM-14479, SAM); 1 ♀, Vlagkop, 5–6 miles north of Hanover [31°S, 24°28'E, SAM database] December 1901–January 1902 (SAM-11924, SAM); *North West Province*: 1 ♀, Barberspan [26.620°S, 25.570°E] 23 February 1991, under bark of blue gum tree (M. Filmer, AcAT 91/805, NCA); 1 ♂, Barberspan [26.620°S, 25.570°E] 25 September 1990, in house (K. Morgan, AcAT 91/758, NCA); 1 ♀, Naauwpoort [26.188°S, 25.577°E; for this and the following record, the SAM database gives alternative coordinates: 32°07'S, 23°00'E, the location of Nelspoort, Western Cape] 12 October 1905 (W. F. P., SAM-B1565, SAM); 1 ♂, Naauwpoort [26.188°S, 25.577°E] 16–17 September 1913 (W. F. Purcell, SAM 1600, SAM); 1 ♀, Vryburg [26°58'S, 22°44'E, SAM database] (SAM, SAM-14291); *Western Cape*: 1 ♂, Anysberg Nature Reserve [33.491550°S, 20.473876°E] September 2007 (C. Haddad & R. Lyle, MF collection #16, DNA sample RMNH.ARA.14515, RMNH); 1 ♀, Anysberg Nature Reserve [33.491550°S, 20.473876°E] September 2007 (C. Haddad & R. Lyle, MF collection #13, DNA sample RMNH.ARA.14519, RMNH); 1 ♀, Beaufort West [32°21'S, 22°35'E, SAM database] 24–29 October 1905 (W. F. Purcell, SAM-B1560, SAM); 1 ♂, Bergvliet [34°3'S, 18°27'E, SAM database] September 1904 (W. F. Purcell, SAM-14207, SAM); 2 ♀, Bergvliet flats, Constantia [34°3'S, 18°27'E, SAM database] December 1899 (W. F. Purcell, SAM-8700, SAM); 1 ♀, Bergvliet, Cape Province [34°3'S, 18°27'E, SAM database] September 1904 (W. F. Purcell, SAM-13896, SAM); 1 ♀, Buffels Bay [33°34'S, 18°21'E, SAM database] [no date] (R. Smithers, SAM-B9112, SAM); 1 ♀, Cape Peninsula, Clifton on Sea [33°56'S, 18°22'E, SAM database] 3 May 1918 (B. Wallace, SAM-B4182, SAM); 2 ♀, Cape Point [34.357°S, 18.497°E] 15 February 1990, caught under *Eucalyptus gomphocephala* bark with +- 40 dead adult beetles (G. Tribe, AcAT 91/499, NCA); 1 ♂, Cape Town [33.95°S, 18.45°E] (MF #11, “born in captivity, offspring of the female no 10 from Cape Town," DNA sample RMNH.ARA.14516, RMNH); 1 ♀, Cape Town surroundings [33.95°S, 18.45°E] (C. Haddad, MF collection #10, “I breed second generation from this female," DNA sample RMNH.ARA.14520, RMNH); 1 ♀, Cape Town [33°56'S, 18°28'E, SAM database] [no date] (SAM-832, SAM); 1 ♀, Cape Town, Cable Way, 33°59'S, 18°30'E, February 1986, tree bark (M. Filmer, AcAT 87/122, NCA); 1 ♀, Cape Town, Green Point [33°54'S, 18°24'E, SAM database] [no date] (H. Pitcher, SAM-B9285, SAM); 1 ♀, Cape Town, Oranjezicht [33°56'S, 18°25'E, SAM database] 30 June 1991 (N. Larsen, SAM-C2215, SAM); 1 ♀, Cape Town, Signal Hill [33°55'S, 18°24'E, SAM database] 1896–1898 (SAM-3539, SAM); 4 ♀, 1 juvenile, Cape Peninsula [34.15°S, 18.4°E] (SAM-B9113, SAM); 1 ♀, De Hoop Nat Reserve [34°28'45"S, 20°30'45"E, SAM database] January 1992 (N. Larsen, SAM-C2411, SAM); 1 ♀, De Hoop Nat Reserve, Bredasdorp [34°28'35"S, 20°30'45"E, SAM database] 17–20 April 1992, under stone (Norman Larsen, SAM-C2507, NL351, SAM); 1 ♀, De Hoop Nat Reserve, Potberg [34.422°S, 20.545°E] 7 April 2004, fynbos under rock (C. Haddad, DNA sample 18-01, AcAT 2006/112, NCA); 3 ♀, 1 juvenile, Devil'S, Peak (North slope) [33°57'S, 18°26'E, SAM database] May 1902 (W. F. Purcell, SAM-12102, SAM); 1 ♀, Devil'S, Peak (North slope) [33°57'S, 18°26'E, SAM database] January 1902 (W. F. P., SAM-12099, SAM); 1 ♀, Devil'S, Peak (North slope) [33°57'S, 18°26'E, SAM database] September 1901 (W. F. Purcell, SAM-9005, SAM); 1 ♂, Farm Tierberg, NE of Prince Albert [33.202°S, 22.069°E] 17 February 2007, Trap 19-1 (M. Burger, M. Fabricius, DNA sample 18-06, AcAT 2008/3804, NCA); 1 ♂, Farm Tierberg, NE of Prince Albert [33.202°S, 22.069°E] 17 February 2007, Trap 19-2 (M. Burger, M. Fabricius, DNA sample 18-08, AcAT 2008/3782, NCA); 1 ♀, Karoo National Park near Beaufort West [32.350°S, 22.580°E] 5 April 1989, web in grass (A. Leroy, AcAT 89/766, LR426, NCA); 1 ♀, Karoo National Park near Beaufort West [32.350°S, 22.580°E] 4 April 1989, web in grass (A. Leroy, AcAT 89/761, LR404, NCA); 1 ♀, Knysna, Southern Comfort, 34°2'S, 23°15'E, 10 April 2006, per hand (L. Lotz, DNA sample 20-02, NMBA 09686, BMSA); 1 ♀, 3 juveniles, Kommetjie, 30 air km S Cape Town, 34°9'S, 18°20'E, 5 April 2001, coastal strand (D. Ubick, S. Prinsloo and K. Muller, CASENT 9039241, CAS); 1 ♀, Leeukoppie, Hout Bay [34°1'S, 18°21'E, SAM database] [no date] (R. Smithers, SAM-B9295, SAM); 1 ♀, Maitland flats [33°55'S, 18°29'E, SAM database] September 1899 (Mrs. [W. F.] Purcell, SAM-6083, SAM); 1 ♀, Mossel Bay [34°11'S, 22°8'E, SAM database] 14 April 1899 (J. L. DrŠje, SAM-5366, SAM); 1 ♀, Mossel Bay [34°11'S, 22°8'E, SAM database] (J. H. Power, SAM-B4594, SAM); 3 ♀, 2 juveniles, Pacaltsdorp, George [34°1'S, 22°27'E, SAM database] 1899 (L. Leipoldt (Pattison), SAM-5126, SAM); 1 ♀, Poortjiesfontein, 5–6 miles N of Hanover [31°S, 24°28'E, SAM database] 1905 (E. Neeser, SAM-14475, SAM); 9 ♀, 8 juveniles, Robin Island [33°48'S, 18°22'E, SAM database] December 1896 (Lightfoot & Purcell, SAM-947, SAM); 1 ♂, route N7, 32.4649°S, 18.96103333°E, 7 October 2009, under the bark on the tree (M. Forman, F. Stahlavsky, MF collection #4, DNA sample RMNH.ARA.14514, RMNH); 1 ♂, route N7, 32.4492°S, 18.95845°E, 7 October 2009, under the bark on the tree (M. Forman, F. Stahlavsky, MF collection #8, “I breed few of their offspring" DNA sample RMNH.ARA.14517, RMNH); 1 ♂, route N7, 32.4649°S, 18.96103333°E, October 2009, under the bark on the tree as juvenile (M. Forman, F. Stahlavsky, MF collection #5, DNA sample RMNH.ARA.14518, RMNH); 1 ♀, 1 juvenile, Signal Hill [33°55'S, 18°24'E, SAM database] [no date] (R. Smithers, Thorne, SAM-B9897, SAM); 1 ♀, Signal Hill [33°55'S, 18°24'E] June 1900 (W. F. Purcell, SAM-8542, SAM); 1 ♀, Somerset West [34°5'S, 18°51'E, SAM database] 17 April 1996 (Anne Gray, SAM-ENW-C003710, SAM); 1 ♀, Stellenbosch [33.930°S, 18.860°E] 2 May 1977, onder klippe (S. Neser, AcAT 77/966, NCA); 1 ♀, 2 juveniles, Swartberg, Nat. Res. Gamkaskloof [33.371°S, 21.843°E] 15 February 2000, on soil (Z. v. d. Walt, DNA sample 18-04, AcAT 2002/181, NCA); 1 ♀, 1 juvenile, Swellendam, Tradouw Pass [33°58'S, 20°42'E, SAM database] November 1925 (SAM-B6896, SAM); 1 ♂, Vanrhynsdorp, 31.611650°S, 18.73591667°E, 29 October 2009, under the bark on the tree (M. Forman, F. Stahlavsky, MF collection #7, DNA sample RMNH.ARA.14513, RMNH); 1 ♂, Van Riebeeck Park, 33°56.901'S, 18°25.182'E, 210 m, 28 February 2006, under rocks in pine forest and fynbos (J. Miller, H. Wood, N. Larsen, DNA sample 09-02, CASENT 9023763, CAS); 1 ♀, Van Wyks Rus [34.050°S, 21.200°E] 19 October 1987, under angle iron frame in web (I. Barone, AcAT 88/106, NCA); 1 ♀, Viljoenshof [farm?], Bredasdorp [34°40'S, 19°41'E, SAM database] 11 August 1939 (S. S. de Klerk, SAM-B9925, SAM); 1 ♀, Worcester Div., Goudini, Schlanghoek [Slanghoek] [33°38'S, 19°13'E] December 1896 (R. Francke, SAM-959, SAM). **Tanzania:** 9 ♀, 25 juveniles, Iringa Distr, Iringa Town, 7°44'46"S, 35°33'15.4"E, 1422 m, 30 May 1997, with one egg sac (ZMUC & SI Exped.1997, ZMUC 19970517, ZMUC); 24 ♀, 11 juveniles, Iringa Distr, Iringa Town [7°44'46"S, 35°33'14.4"E] 1422 m, 17–27 May 1997 (ZMUC & USNM Exp.1997, ZMUC 19970530, ZMUC). **Zimbabwe:** 1 ♂, Harare, 19 Walmer Drive, 17°15'S, 31°2'E, 15 January 2002, on soil, hand (M. Cummings, AcAT 2005/123, NCA, DNA sample 18-05).

*Loureedia annulipes* (Lucas, 1857)

Primary types

*Eresus annulipes*Lucas, 1857

Patria Ignota [unknown site] (AR5391, NMHN, holotype). The original description gives the locality as “environs de Rio-Janeiro “ (Lucas 1857: 21–22). This contradicts the label on the type specimen noted above and has led to the long held and erroneous notion that this species is from Brazil. Also, the original paper indicates that this species is described from the female, but the type specimen is male.

*Eresus semicanus* Simon, 1908

**Egypt:** 1 ♂, 1 ♀, Alexandrie [no date] (AR836, original code 471, original label written by Simon, NHNM, syntypes). **Tunisia:** 2 ♀, Djerba [no date] (Ltr. [Latreille], AR835, original code 12470, identified as *Eresus petagnae* Aud. [*Erigone petagnae* Audouin, 1826], original label written by Simon, NHNM, syntypes, same specimens are syntypes of *Erigone jerbae*).

*Eresus jerbae* El-Hennawy, 2005

**Tunisia:** 2 ♀, Djerba [no date] (Ltr. [Latrelie] AR835, original code 12470, identified as *Eresus petagnae* Aud. [*Erigone petagnae* Audouin, 1826], original label written by Simon, NHNM, syntypes, same specimens are syntypes of *Erigone semicanus*).

Additional specimens examined

**Israel:** 1 ♀, Negev, Wadi Mashash, 4 December 2004, J. Kral (MR019, MR); 1 ♀, Haluquim, army wadi, 18 November 2004 (PET03, MR); 1 ♂, Haluqim Ridge (n. Sede Boqer), 17 November 1990, Y. Lubin (MR008, HUJ); 1 ♂, Negev, Nitzana [Nitzanna] village, 1 October 2004 (MR018, HUJ).

*Paradonea striatipes* Lawrence 1968

Primary type

**Namibia:** 1 ♂, Runtu [Rundu] on the Okavango river, north-east border of S.W. Africa [17.93°S, 19.76°E], 24 April 1967 (J. L. Sheasby, No. T. 639, TMSA, holotype).

Additional specimens examined

**Namibia:** 1 ♂, Outjo, Kamandjab 19°37'S, 14°51'E, 20 March 1992, in town, on road (J. Irish & E. Marais, NMBA 6035, new nr. NMBA 05700, BMSA); 1 ♂, Otjivasandu [19°31'S, 14°48'E], 29 May 1966 (J. Meyer, NMN). **Republic of South Africa:***Northern Cape*: 1♂, Kathu [27.70°S, 23.05°E], 30 March 2010 (L. Fourie, AcAT 2010/1955, NCA); 1♂, Dithakong Tribal Land, NE of Kuruman, 24 March 2006, pit and funnel trap (M. Burger, AcAT 2009/3152, NCA).

*Paradonea splendens* Lawrence, 1936

Primary type

**Botswana:** 1♂, Gemsbok Pan [24.97°S, 21.84°E] [no date] (U. Fitz Simons, TM5913, TMSA, holotype).

Additional specimens examined

**Republic of South Africa:** *Gauteng?*: 1 ♂, Sunnyside [25.73°S, 28.21°E] (C1076, SAM); *Northern Cape*:2♂, Benfontein, Kimberley [28.72°S, 24.75°E], 15 March 1981, on soil, pit traps (S. Erasmus, AcAT 2008/159, NCA).

*Paradonea variegata* (Purcell, 1904)

Primary type

*Adonea variegata* Purcell, 1904

**Republic of South Africa:** 3 ♀, Cape Colony, Namaqualand Div., Great Bushmanland, Naroep [29.34°S, 21.14°E], March, 1989 (Max Schlecter, No. 3701, SAM, syntypes).

Additional specimens examined

**Botswana:** 1♀,nr Francistown, Selkirk Mine, 21.3222°S, 27.7062°E, November 2007, EIA: site 07: pitfall (D. H. Jacobs, AcAT 2009/177, NCA). **Namibia:** 1 ♂, app. 50 km SW Aus (on road C13), 27.0473°S, 16.391°E, 9 October 2009 (M. Forman, F. Stahlavsky, MF collection #2, "Nest with app. Ten juveniles under the stone, breed in captivity final moulting (11.1.2011) I still have few living juveniles from this nest", DNA sample RMNH.ARA.14512, RMNH); 1 ♀, app. 50 km SW Aus (on road C13), 27.0473°S, 16.391°E, 9 October 2009 (M. Forman, F. Stahlavsky, MF collection #3, "Nest with app. ten juveniles under the stone, breed in captivity final moulting (11.1.2011) I still have few living juveniles from this nest", DNA sample RMNH.ARA.14522, RMNH). **Republic of South Africa:** 1 ♂, Kenhart Div., Great Bushmanland, Namies [29.34°S, 21.14°E], March 1898 (Max Schlechter, SAM 3716, SAM); 1 ♀, mid plateau, Kruger National Park [23.24°S, 31.29°E], 7 April 1994, under fallen grass (A. Leroy, LR 1265, AcAT 98/278, NCA); *Northern Cape*: 1 ♂, Breekkierie Dunes, 30°07'S, 21°33'E, 4 May 1985 (M. Macpherson, C1062, shelf no: SAM/Aran 3147, SAM); 9 ♀, 25 juveniles, Komaggas [29.67°S, 17.54°E], July, 1904 (S. Schulze col. no. 673, ZMB 26963, ZMHB); 5 ♀, 39 juveniles, Komaggas [29.67°S, 17.54°E], July, 1904 (S. Schulze col. no. 676, ZMB 26969, ZMHB); 2 ♀, 4 juveniles, Komaggas [29.67°S, 17.54°E], July, 1904 (S. Schulze, ZMB 26970, ZMHB); 1 ♀, 4 juveniles, Steinkopf [29.26°S, 17.73°E], July, 1904 (S. Schulze col. no. 716, ZMB 26968, ZMHB); 22 ♀, 21 juveniles, Steinkopf [29.26°S, 17.73°E], July, 1904 (S. Schulze col. no. 717, ZMB 26964, ZMHB); 2 ♀, 3 juveniles, Steinkopf [29.26°S, 17.73°E], August, 1904 (S. Schulze col. no. 739, ZMB 26967, ZMHB); *Western Cape*: 1 ♀,1 juvenile, Dwars River nr. Cedarberg [32.31°S, 19.15°E], 4 September 1993, burrow under rocks (A. Le Roy, LR 1097, AcAT 94/554, NCA).

Additional records

**Republic of South Africa:** 1 ♀, Namaqualand Div., Great Bushmanland, Kykgat, March, 1898 (Max Schlechter, not examined); 2 ♀, Calvinia Div., Bokkeveld Mountains about Nieuwoudtville [31.38°S, 19.10°E] (M. Schlechter, August, 1897; C. L. Leipoldt, August 1897, not examined); 3 ♀, 1 juvenile, Worcester Div. Touws River [33.64°S, 19.43°E], August 1903 (R. M. Lightfoot and W. F. Purcell, not examined); 1 ♀, Worcester Div., Matjesfontein [33.55°S, 26.33°E], August 1903 (W. F. Purcell, not examined).

*Paradonea parva* Tucker, 1920

Primary type

**Republic of South Africa:** *Limpopo*:1♂, N.W. Transvaal, Junction of Marico and Crocodile Rivers [24.19°S, 26.87°E], January–February 1918 (B3701, SAM, holotype).

Additional specimens examined

**Botswana:** 1♂,nr Francistown, Selkirk Mine, 21.2897°S, 27.7631°E, 7 November 2007–29 February 2008, EIA: site 14: pitfall (D. H. Jacobs and M. Stiller, AcAT 2009/178, NCA). **Republic of South Africa:** *Northern Cape*:2♂, 4 km N of Hopetown [29.59°S, 24.08°E], December 1996–February 1997, mixed karooveld, pit traps (B. Chambers, AcAT 97/988, NCA); 2♂, Benfontein, 10k S Kimberley [28.82°S, 24.75°E], November–December 1980, pit traps (S. Erasmus, AcAT 81/1003, NCA).

*Paradonea presleyi* sp. n.

Primary type

**Zimbabwe:** 1 ♂, Falcon College, 20°18'S, 29°E, 10 November 1990 (V. [and] B. Roth, CASENT 9039236, CAS, holotype).

Additional specimens examined

**Republic of South Africa:** 1 ♂, Kruger National Park [23.82°S, 31.45°E], 29 November 2005, pit traps PCZ (K. Harris, AcAT 2008/4480, NCA, paratype).

*Seothyra henscheli* Dippenaar-Schoeman, 1991

Specimens examined

**Namibia:** 2 ♀, Gobabeb, Kuiseb River at 23°33'57.5"S, 15°02'31.0"E, 363 m, 10 June 2007 (T. L. & G. Bird & H. Vollebrecht, SMN 46627, NMN); 1 ♂, Gobabeb Station (inside house), 23°34'S, 15°02'E, 5 June 1988 (J. R. Henschel, CA0415, SMN 40828, NMN); 1 ♀, Sout Rivier, near Gobabeb [23.56°S, 15.04°E], 14 April 1993 (J. Henschel, CASENT 9039242, CAS).

*Stegodyphus lineatus* (Latreille, 1817)

Specimens examined

**Afghanistan:** 1 ♂, Nengrahar, 10 km OOS Jalalabad [34.17183°N, 70.62167°E], 19 February 1966, 620 m (Povolny A. Tenora, MR010, MR). **Israel:** 1 ♂, Negev [31°N, 34.75°E] [no date] (Y. Lubin, MR). **Turkey:** 2 ♀, Belkis, near Birecor, 30 June 2004, slopes on right bank of dam on Enfrat, steppe [36.92466°N, 31.14383°E] (M. Řezáč, MR015, MR).

*Stegodyphus mimosarum* Pavesi, 1883

Specimens examined

**Madagascar:** *Fianarantsoa*: 9 ♂, 93 ♀, 4 juveniles, Forêt d'Analalava, 29.6 km 280° W Ranohira, 22°35'30"S, 45°7'42"E, el 700 m, 1–5 February 2003, tropical dry forest EF37 general collecting day spiders (Fisher, Griswold et al., BLF07389, CASENT 9015950, CAS); 2 ♂, 22 ♀, Forêt d'Analalava, 29.6 km 280° W Ranohira, 22°35'30"S, 45°7'42"E, el 700 m, 1–5 February 2003, dry forest on sandy soil, general collecting, beating/puffing spiders (col. Fisher, Griswold, et al., BLF 7390, CASENT 9005869, CAS); *Toliara*: 2 ♂, 40 ♀, Réserve Spéciale de Cap Sainte Marie, 14.9 km 261° W Marovato, 25.59444°S, 45.14683°E, 160 m, 13–19 February 2002, spiny forest/thicket EH26 general collecting night spiders (Fisher-Griswold Arthropod Team, BLF05653, CASENT 9012844, CAS). **Malawi:** 2 ♂, 1 ♀, 31 mi. SE Ft. Hill [9.72°S, 33.27°E], elev. 1600 m, 20 February 1958 (E. Ross, CAS).

*Stegodyphus sarasinorum* Karsch, 1891

Specimens examined

**Myanmar:** 2 ♂, 16 ♀, 46 juveniles, Magway Division, 7.5 km E PwintPhyu (=Pwinbyu) 20°25'26.8"N, 94°40'11.2"E, el 79 m, 27 September 2003, from communal nests on *Acacia* trees (D. Ubick, C. Griswold, CASENT 9019370, CAS).

*Stegodyphus* sp.

Specimens examined

**Myanmar:** *Magway Division*: ShweSettaw Wildlife Reservation, 20°5'51.1"N, 94°33'24.5"E, elev. 450 ft., 28–29 September 2003, deciduous forest, general collecting (C. Griswold, P. Sierwald, D. Ubick, Aye Aye Cho and Tin Mya Soe, Dong Lin photo voucher, CAS; Fig. 3I).
